# Temporal Evolution and Associated Factors of Adherence to Mammography Screening among Women in Spain: Results from Two National Health Surveys (2017–2020)

**DOI:** 10.3390/healthcare11222934

**Published:** 2023-11-09

**Authors:** Silvia Portero de la Cruz, Luis M. Béjar, Jesús Cebrino

**Affiliations:** 1 Department of Nursing, Pharmacology and Physiotherapy, Faculty of Medicine and Nursing, University of Córdoba, Avda. Menéndez Pidal, S/N, 14071 Córdoba, Spain; 2 Research Group GE10 Clinical and Epidemiological Research in Primary Care, Instituto Maimónides de Investigación Biomédica de Córdoba (IMIBIC), Hospital Universitario Reina Sofía, 14071 Córdoba, Spain; 3 Department of Preventive Medicine and Public Health, Faculty of Medicine, University of Seville, Avda. Doctor Fedriani, S/N, 41009 Seville, Spain; lmbprado@us.es (L.M.B.); jcebrino@us.es (J.C.)

**Keywords:** breast neoplasms, guideline adherence, healthcare disparities, mammography, mass screening

## Abstract

Ensuring equity in cancer screening is recommended by the European Commission. Despite the fact that breast cancer screening is a free population-based program in Spain, there remains considerable variation in the adherence to screening rate among population groups. This study was designed to describe the adherence of breast cancer screening in women in Spain, to evaluate the evolution in the period from 2017 to 2020, and to determine the variables that influence choosing to undergo breast cancer screening. A nationwide cross-sectional study with 7220 females aged 50–69 years from the 2017 Spanish National Health Survey and the 2020 European Health Survey for Spain was performed. We investigated mammography uptake rates, with socio-demographic factors, lifestyle habits, and health-related characteristics as independent variables. Multivariable logistic regression was used to identify the associated factors of mammography adherence. Of the women, 78% had undergone mammography in the previous two years, and there was a significant decrease in the uptake rate for breast cancer screening from 2017 (81.23%) to 2020 (74.68%) (*p* < 0.001). Educational level, marital status, residential location, nationality, smoking status, alcohol consumption, and leisure-time physical activity were all associated factors of mammography uptake.

## 1. Introduction

Breast cancer is the most frequent disease and the leading cause of cancer mortality globally. By 2040, the number of newly diagnosed cases is projected to increase by over 40%, while deaths are expected to rise by more than 50% [1]. In Europe, an estimated 384,800 women were diagnosed with breast cancer, and 98,500 died from this cause in 2022 [2]. Surprisingly, the death rates for breast cancer in Spain are among the lowest among European Union member countries [3].

Nowadays, despite the decreased sensitivity of mammography shown in women with high breast density (which varies from 43 to 60%) [4], mammography is the only method used for screening [5], which has been shown to reduce breast cancer-related mortality [6,7,8], enabling early breast cancer therapy and limiting the need for severe treatment such as mastectomy [9]. In Spain, a population screening program for breast cancer based on biennial mammography is available to females aged 50 to 69 who have had no family history of the disease [10]. At the age of 40, women with risk indicators are asked to join the program. Nevertheless, several private healthcare centers provide yearly mammograms from the age of 40 [11]. Currently, all women in the target group are individually invited every two years (by post and/or phone) to have a mammogram [12].

Previous studies have found that population-based cancer screening programs have led to a reduction in breast cancer mortality [13,14]. Nevertheless, the success of cancer screening programs is determined by the uptake rate among target groups [15]. As a result, it is important to research screening adherence and discover the factors that influence having breast cancer screening performed. Various Spanish research projects in this area have found varying adherence rates for mammography screening (from 72% to 80%) [11,16], with increased (82.6%) [16] and decreased (62%) [14] involvement in recent years. On the other hand, some determinants of mammography screening attendance are suggested by the scientific literature, including women’s socioeconomic level and certain lifestyle choices [17]. The lower involvement of less educated women is especially striking [18]. Similarly, ethnic minorities [19], rural living [20], and less healthy lifestyle behaviors [21] are linked to lower rates of screening attendance. Nonetheless, at this time, it is critical to understand the positive and negative characteristics related to breast cancer screening adherence as this will provide guidance for local health care providers and health departments in developing and implementing steps to reduce this imbalance.

The Spanish population may be regarded as a suitable target group for examining the variables related to the screening program adoption rate [14]. The current study’s main goals were to describe the adherence of breast cancer screening in women in Spain, to evaluate the evolution in the period from 2017 to 2020, and to determine the variables that influence having breast cancer screening performed.

## 2. Materials and Methods

Data from the Spanish National Health Survey (SNHS, 2017) [22] and the European Health Survey for Spain (EHSS, 2020) [23] were used to carry out a cross-sectional study. The data for the SNHS 2017 were gathered from October 2016 to October 2017, and the data for the EHSS 2020 were gathered from July 2019 to July 2020. Both surveys were conducted by the National Statistics Institute, under the auspices of the Spanish Ministry of Health and Social Affairs, and both were home-based computer-assisted personal interviews that obtained a representative sample of non-institutionalized persons (aged 15 and above) who lived in family homes (households) in Spain. The sampling design was multistage probabilistic, stratified by census areas (first stage), family homes (second stage), and individuals (third stage). An adult from each household was randomly chosen to complete the survey and was mailed the rationale behind the questionnaire, as well as the characteristics and anonymous nature of their participation.

For the current research, we selected 7220 women aged 50–69 years for mammography update evaluation (SNHS 2017: n = 3709; EHSS 2020: n = 3511) based on screening guideline age groups (18). The sample initially included 7902 women aged 50–69 years, of whom 682 respondents (9.45%) were later excluded owing to their reluctance to answer the questions (SNHS 2017: n = 320; EHSS 2020: n = 362), despite having identical characteristics to the other women.

The variables were determined by the questions included in the questionnaires, which were the same in all the surveys.

The dependent variable was breast cancer screening uptake, which was measured by asking, “Have you ever had a mammogram?” Those who said yes were then asked, “When was the last time you had a mammogram?” According to the classification of women who complied with the recommended screening period [24], those who reported having their most recent mammography within the past two years were called “uptakers”. The remaining participants were labeled “non-uptakers”.

The independent variables were as follows:

– Socio-demographic: age group (50–56/57–63/64–69 years), educational level (without studies/primary/secondary/university), marital status (single/married/widowed/separated or divorced), residential location (rural/urban) [25], nationality (Spanish/foreigner), and social class. The social class was determined based on the neo-Weberian classification, the origins of which lie in the occupation of the primary breadwinner as developed by the Working Group on Determinants of the Spanish Society of Epidemiology [26]: upper social class (directors and managers of companies with 10 or more employees and professionals normally qualified with university degrees, directors and managers of companies with fewer than 10 salaried employees, professionals normally qualified with university degrees, other technical support professionals, athletes, and artists), middle social class (intermediate professions and self-employed workers, supervisors and workers in skilled technical work), and lower social class (skilled workers in the primary sector and other semi-skilled workers and unskilled workers).
– Health status: number of chronic conditions (0/1/≥2), self-perceived health status (very good/good, fair, poor, very poor), type of healthcare insurance (public/private), and visits to the primary care physician in the previous four weeks (yes/no). The existence of physician-diagnosed mental disorders, such as chronic anxiety, chronic depression, and other mental diseases, was assessed based on self-reported occurrence. Any woman diagnosed with one or more of these three disorders was labeled as “suffering a mental illness”.
– Lifestyle behaviors: body mass index (underweight/normal weight/overweight/obesity) [27], smoking status (never smoker/former smoker/current smoker), alcohol use (never/former/current), and leisure-time physical activity (non/lower [occasional–several times a month]/higher [several times a week]).

The anonymized data are available to the general public on the website of the Ministry of Health, Consumer Affairs and Social Welfare [22,23]. The study was evaluated by the Research Ethics Committee of Córdoba (Spain), and it was determined that ethical approval was not necessary because secondary data were used, and the database was obtained from the website of Spanish Ministry of Health, which is accessible to the public.

The categorical variables were expressed as frequencies and percentages. Chi-squared test was used to make comparisons in the variable distributions between 2017 and 2020 and to detect significant changes in mammography adherence from 2017 to 2020. Last, a binary logistic regression was performed. The dependent variable was adherence to mammography screening, with two possible values: uptakers (yes) and non-uptakers (no). The independent variables were socio-demographic factors, health status, and lifestyle behaviors. Those factors with >2 categories were incorporated into the model via dummy variables.

The independent variables were introduced one by one into the crude model. Those with a potential relationship with dependent variable (*p* ≤ 0.15) were included in the multivariable model, and non-significant variables were excluded, using backward selection based on the likelihood of the Wald statistic. All possible interaction terms between variables in the logistic regression model were taken into consideration. The crude and adjusted odds ratios (ORs) with their 95% confidence intervals were used to measure the strength of association. The Nagelkerke R^2^, Hosmer–Lemeshow statistics, and −2 log likelihood (−2 LL) change in deviance were calculated as measures of model fit. The level of statistical significance was fixed at α = 0.05. All analyses in this study were based on the unweighted data due to the nature of our research question and also following the recommendations of other authors [28]. SPSS 25.0 software, licensed to the University of Córdoba (Spain), was used to conduct the statistical analysis.

## 3. Results

The records of 7220 women residing in Spain over 50 years of age were analyzed, resulting in a mean age of 59.15 (SD ± 5.69) years old. In 2017, and compared with 2020, women were more frequently married (2017: 63.39%, 2020: 58.93%, *p* < 0.001), belonged to the lower social class (2017: 48.58%, 2020: 45.54%, *p* < 0.01), had primary studies (2017: 22.54%, 2020: 19.11%, *p* < 0.001), lived in rural settings (2017: 42.68%, 2020: 45.00%, *p* = 0.04), perceived a better health status (2017: 8.41%, 2020: 6.58%, *p* < 0.01), had ≥2 chronic conditions (2017: 31.63%, 2020: 26.92%, *p* < 0.001), visited a general practitioner (2017: 34.92%, 2020: 26.55%, *p* < 0.001), and were overweight (2017: 36.07%, 2020: 35.63%, *p* = 0.02). Table 1 shows the uptake rates for mammography based on socio-demographic characteristics, health-related status, and lifestyle behaviors. Compliance with mammography practice was higher in women who were married, were born in Spain, had a university education, belonged to the upper class, and lived in rural settings. Moreover, a higher mammography adherence was found among women with private health insurance, those who visited a general practitioner in the four weeks preceding the survey completion, and those who had two or more chronic diseases. However, lower uptake was associated with former alcohol use, being underweight, being a current smoker, and not doing physical activity during leisure time.

**Figure 1:**
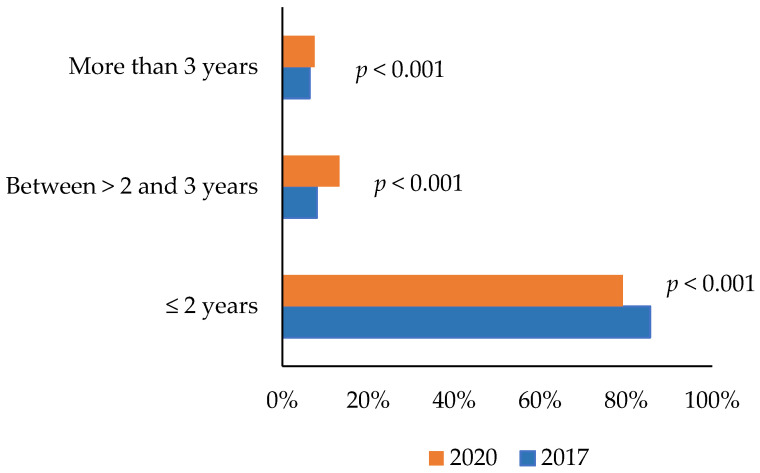
Frequency of mammography adherence in the period 2017–2020 in Spain.

Of the total of women aged 50–69 years, 78% had received breast cancer screening in the previous two years. There was a significant decrease in mammography adherence during the previous two years in 2020 (79.22%) with respect to 2017 (85.59%) (*p* < 0.001). Moreover, the percentage of women who attended the screening between >2–3 years (2017: 8.04%, 2020: 13.29%, *p* < 0.001) and more than 3 years (2017: 6.36%, 2020: 7.49%, *p* < 0.001) was lower in 2017 than in 2020 (Figure 1).

In general, the mammography uptake rate decreased from 2017 to 2020 in each group of analyzed variables, except for people with university studies and those who were single, were foreigners, had public insurance, belonged to the upper social class, and had a self-perceived very poor health status, in which the mammography uptake rate did not vary over time. On the contrary, the compliance increased from 2017 to 2020 among women who had private insurance (Table 2).

A number of determinants were associated with mammography adherence in 2017 and 2020 (Table 3). In 2017, women with secondary and university studies had 37% and 45% higher odds of compliance (OR = 1.37, 95% CI 1.01–1.85 and OR = 1.45, 95% CI 1.02–2.07, respectively). Being married was associated with 86% higher odds of adherence (OR = 1.86 95% CI 1.46–2.38). Similarly, current alcohol consumption and higher levels of physical activity during leisure time were both associated with 30% and 46% higher odds of mammography uptake (OR = 1.30, 95% CI 1.19–1.75 and OR = 1.46, 95% CI 1.16–1.82, respectively). On the contrary, the odds of adherence decreased 63% among foreigner women (OR = 0.37, 95% CI 0.26–0.52), 27% in the case of current smokers (OR = 0.73, 95% CI 0.59–0.89), and 16% in women who lived in rural settings (OR = 0.84, 95% CI 0.71–0.93). The probability of adherence to mammography screening is given by the following equation:

P (adherence) = 1/(1 + e^−z^)

Z = logit (P) = 0.508 + 0.17X_1_ + 0.32X_2_ + 0.37X_3_ + 0.62X_4_ + 0.75X_5_ + 0.27X_6_ − 0.17X_7_ − 0.99X_8_ + 0.02X_9_ − 0.32X_10_ − 0.33X_11_ + 0.26X_12_ + 0.19X_13_ + 0.38X_14_

In 2020, while most of the factors associated to adherence remained consistent with those identified in 2017, certain determinants, such as the presence of 1 or ≥2 chronic conditions and having private insurance showed an increase in the odds of compliance with mammography screening of 35%, 39%, and 56% (OR = 1.35, 95% CI 1.13–1.63; OR = 1.39, 95% CI 1.14–1.70; OR = 1.56, 95% CI 1.05–2.34, respectively). The following equation shows the probability of adherence to breast cancer screening:

P (adherence) = 1/(1 + e^−z^)

Z = logit (P) = −0.004 + 0.38X_1_ + 0.60X_2_ + 0.75X_3_ + 0.34X_4_ + 0.15X_5_ + 0.05X_6_ − 0.22X_7_ − 0.91X_8_ + 0.30X_9_ + 0.33X_10_ + 0.33X_11_ + 0.45X_12_ + 0.22X_13_ − 0.11X_14_ − 0.19X_15_ + 0.16X_16_ + 0.18X_17_ + 0.32X_18_

In none of the logistic regression models were the interaction terms statistically significant (*p* > 0.05).

Table 4 shows the logistic regression model for independent associated factors of mammography screening adherence in both years. Having completed secondary and university studies were associated with 57% and 84% higher odds of mammography uptake (OR = 1.57, 95% CI 1.26–1.89 and OR = 1.84, 95% CI 1.37–2.27, respectively). Also, married participants had 60% higher odds of compliance (OR = 1.60, 95% CI 1.36–1.90). Likewise, the year 2017 and having visited a general practitioner were associated with 48% and 13% higher odds of mammography adherence (OR = 1.48, 95% IC 1.32–1.67 and OR = 1.13, 95% CI 1.05–1.35, respectively). Moreover, the odds of adherence increased 20% and 30% among women who had 1 or ≥2 chronic diseases (OR = 1.20, 95% CI 1.04–1.37 and OR = 1.30, 95% CI 1.13–1.50, respectively). Also, current alcohol consumption and higher levels of physical activity during leisure time were both associated with 34% higher odds of mammography uptake (OR = 1.34, 95% CI 1.19–1.51 and OR = 1.34, 95% CI 1.15–1.56, respectively). In contrast, the odds of adherence decreased 18% among women who lived in rural settings or were current smokers (OR = 0.82, 95% CI 0.73–0.92 and OR = 0.82, 95% CI 0.73–0.92, respectively) and 62% among foreigners (OR = 0.38, 95% CI 0.30–0.49). The probability of adherence to screening is depicted in the following equation:

P (adherence) = 1/(1 + e^−z^)

Z = logit (P) = −0.025 + 0.39X_1_ + 0.26X_2_ + 0.45X_3_ + 0.61X_4_ + 0.47X_5_ + 0.42X_6_ + 0.14X_7_ − 0.20X_8_ − 0.97X_9_ + 0.18X_10_ + 0.26X_11_ + 0.12X_12_ + 0.12X_13_ − 0.20X_14_ − 0.02X_15_ + 0.29X_16_ + 0.27X_17_ + 0.29X_18_

None of the interaction terms were statistically significant (*p* > 0.05).

## 4. Discussion

### 4.1. Main Findings

The present population-based study performed in Spain describes the uptake of mammography and identifies the factors associated with breast cancer screening compliance in a sample of women from 2017 to 2020.

The data analyses showed that 78% of women aged 50–69 years had undergone breast cancer screening in the previous two years. This adherence is higher than the European mean participation rate (60.2%) and is considered desirable, according to the European recommendation on breast cancer screening [29]. Although the total examination coverage varies from 49% in Eastern Europe to 69% in Southern Europe [14], it is difficult to establish a comparison of breast cancer screening adherence among European Union countries due to the considerable differences in the target populations and screening strategies. The highly extended screening as is observed in Spain and the improvements in access to healthcare services have a favorable impact on the early diagnosis of breast cancer at more localized stages, all of which lead to a reduction in mortality [14,30]. On the other hand, the adherence to mammography screening decreased by 6.37% from 2017 (85.59%) to 2020 (79.22%) in the current study. Given that the last five months of the 2020 data collection occurred during the COVID-19 pandemic, this result is consistent with similar studies in which reductions in screening mammography rates ranged from 2.7% to 100% during the pandemic period [31,32]. Particularly, screening uptake reduced by 35–100% during the pandemic peak in March–May 2020 [31,33,34]. The findings mentioned above illustrate the impact of the pandemic on populations and the global health system’s response. This response included the suspension of screening programs, the closure of non-urgent healthcare services, and the implementation of regional lockdowns, all of which contributed to some of the initial reductions. While these response measures increased health system capacity for improved COVID-19 containment and mitigation, they also had some negative consequences on the provision of healthcare services to the general population, especially cancer prevention and control initiatives [35]. Additional factors that could have played a significant role in the decrease, apart from the healthcare system’s response, include anxiety and the fear of COVID-19 infection [36], which also likely explains the observed reductions in early diagnosis rates [37]. Another possible reason for this reduction could be the greater effort put forth to develop and implement colorectal screening programs by the National Health System. Colorectal screening is being progressively implemented in Spain to cover the whole population and takes up considerable resources, which might otherwise be used to maintain the quality standards of breast cancer screening programs [38]. Whatever the reason, it is a high priority to identify the reasons for this decreased participation in order to act accordingly.

This study enabled us to investigate the variables that influenced breast cancer screening uptake. We observed that women with higher education reported greater screening participation. Although prior research did not discover a link between educational attainment and breast cancer screening uptake [39], the results obtained in the current study agreed with previous evidence [39,40,41]. People with a higher level of education perceived good financial status and had a better ability to learn about preventive practices [42]. According to Willems and Bracke [43], in countries with nationally organized screening programs for breast cancer, such as Spain, women with higher levels of education are more likely to follow their general practitioner’s advice rather than a screening program, when compared with their less educated counterparts. To support this, and consistent with our findings, past research has also demonstrated that increased engagement with the primary care physician promotes breast cancer screening participation [44]. This could represent an indirect result of general practitioners recalling, educating, and encouraging screening to the women [45]. These findings underscore the relevance of primary care for overall screening efforts, as well as the relevance of radiology departments working closely with primary care departments to enhance accessibility and promote the use of mammography.

Regarding the place of residence, research on breast cancer screening utilization among urban and rural women is variable and dissimilar. Our findings showed a reduced utilization of breast screening among rural women. This was consistent with previous findings in mammography in urban versus rural populations [46,47]. Numerous obstacles to cancer screenings are prevalent in rural communities. The distance to mammography facilities can be a significant factor, as shown by Chandak et al. [48], who revealed that certain rural regions had greater distances to mammography centers, leading to increased rates of late-stage cancer diagnoses. On the other hand, although rural women have fewer mammography facilities close to their homes and longer driving times to travel to the facilities, which decrease breast cancer screening frequency [47], Jewett et al. [49] reported that the presence of one or two mammography facilities near women’s homes may enhance mammography uptake but that the benefits decrease with more than two nearby facilities. Another possible explication for the lower mammography adherence found in the current research among rural women is the higher frequency of fatalistic ideas regarding cancer prevention in rural regions compared with urban areas [50].

When considering the civil status, married participants had 60% higher odds of compliance, which was consistent with previous studies across different countries [51,52,53]. It is thought that married persons tend to have a wider social network, which provides them with more emotional and practical support to seek preventive testing, as well as assisting them in adopting healthier behaviors [15,54].

Our study revealed that the greatest comorbidity was linked with increased involvement in mammography screening. Nevertheless, some authors demonstrate that women with two to four coexisting health disorders are less likely to receive a mammography screening than those who do not have comorbidities and suggest that higher workload, administrative demands, and the difficulty of managing numerous comorbidities might result in physician exhaustion, thereby compromising the screening implementation [55]. The observed outcome in our study might be explained in a variety of ways. On one hand, because healthy women have fewer overall healthcare visits, they have fewer chances of hearing advice about the advantages of mammography compared with women with comorbid diseases. On the other hand, healthy women who have less contact with the health sector may depend more on mass culture, where messages about the advantages and dangers of mammography are frequently inconsistent [56]. Another noteworthy feature is that even women with chronic conditions that are well managed may not survive long enough to benefit from early identification [57]. This point emphasizes the importance of considering tailored screening that takes into consideration the severity of chronic conditions, as well as the women’s age and interests, in order to maximize the benefit gained from screening and optimize healthcare services.

When analyzing nationality, it is crucial to consider that immigrants make up around 11.25% of Spain’s overall population [58], and evidence suggests that immigrant women continue to have lower rates of mammographic screening [59], despite the significance of breast cancer screening in the early identification and treatment of breast cancer. This is in line with our findings. Several variables impact this condition, including socio-economic uncertainty, lack of understanding about the Spanish National Health System, and language issues [60].

Regarding the type of healthcare insurance, having private insurance increased 55% the odds of adherence to mammography screening in 2020. The COVID-19 pandemic and, consequently, the collapse in public healthcare led to a 5.29% increase in private insurance usage among women in 2020 compared with 2017 [61,62]. Individuals with private insurance typically choose this coverage to avoid waiting times and gain direct access to specialists [63]. Our finding possibly suggests that economic position is one of the factors influencing the decision to undergo mammography [64].

In the present study, certain lifestyle behaviors were associated with adherence to breast cancer screening. For example, our results revealed that physical activity was linked to a greater uptake rate for mammography screening, in line with past studies [65,66]. This might be explained by the fact that women who are concerned about their physical appearance and health are more likely to engage in physical activity. Previous research has found a link between mammography utilization and smoking status [67,68]. We found that the odds of adherence decreased 18% among women who were current smokers. In that sense, smokers’ perceptions of large obstacles to care may be associated to lower participation in cancer prevention services [69]. Also, women who smoke might be less motivated to seek further health screenings while managing their tobacco addition; therefore, their smoking habits could impact their limited participation in preventative programs [70].

Additionally, we observed that current alcohol users were associated with 34% higher odds of mammography uptake. At first glance, this may appear contradictory; nonetheless, moderate alcohol intake is widespread among women with a high socioeconomic position in Spain, which in turn is connected with increased cancer screening uptake [15]. Although our results support previous findings [71], the data about the effect of alcohol consumption on breast cancer screening adherence are inconsistent. Some authors did not find any association [72,73], while others revealed a negative association [20,74]. This suggests that more research is needed to clarify the relationship between alcohol consumption and screening participation.

### 4.2. Strengths and Limitations

Our findings were susceptible to several limitations, which should be highlighted. First, because a cross-sectional design was used, causality could not be inferred. Second, the use of self-reported variables caused the presence of memory and social desirability biases to be more likely. Third, the representativeness of sample may have been compromised because a multistage stratified sampling design was applied and unweighted data were used. Fourth, analyzing the complete cases as a method to handle missing data could have produced biases in the point estimation and larger sampling variances. Thus, these findings should be considered only as hypothesis-generating results. Fifth, there was a lack of information in the surveys about the screening invitation coverage among non-uptakers, which may have affected the uptake rate result. Finally, since mammography can be used for screening or diagnostic purposes, it was not possible to determine from the used surveys why these women received a mammogram. Nonetheless, the use of a national sample of the Spanish population to measure breast cancer screening adherence and the examination of a significant number of socio-demographic variables, lifestyle behaviors, and health-related factors not found in other health care records were strengths of this study.

### 4.3. Implications for Research and Practice

The findings could have important clinical implications. Although mammography adherence in Spain is satisfactory, there was a decline in participation in 2020 compared with 2017. The most concerning possible negative consequence of the decline in participation would be a rise in cancer morbidity and death in the future. Disruptions in cancer screening programs might delay tumor diagnoses, resulting in a higher number of cancer cases detected at more advanced stages in the following screening round for women who missed the pandemic round, which could lead to a less favorable prognosis. Furthermore, this could probably raise the number of avoidable deaths from cancer and healthcare costs. It remains uncertain if the proposed initiatives to minimize the backlog of women who missed screening during the pandemic, such as calling them to make an appointment, will aid in the detection of tumors missed during screening disruption. Given this situation, it is crucial to take actions to reestablish cancer screening as a fundamental component of preventive healthcare, particularly among people with lower socioeconomic status, such as removing geographical obstacles, enhancing the participation of primary care physicians, and implementing personalized proactive communication. Since there is an unfortunate potential for future pandemics produced by viral or other outbreaks, the prioritization of eligible individuals appears to be a particularly valuable strategy, especially in situations with limited resources, where maximal efficiency is essential. The objective would be to give preference to individuals who have a higher probability of developing cancer and, consequently, receiving positive screening results: women who have not had a checkup in many years or those from disadvantaged social and economic backgrounds facing substantial barriers to healthcare access and, as a result, having a reduced inclination to seek screening on their own.

## 5. Conclusions

Despite the fact that breast cancer screening is a free population-based program in Spain, our results suggest that there was a decline in participation in 2020 compared with 2017 and the influence of sociodemographic, health status, and lifestyle factors in compliance, suggesting that there may still be social differences in screening adherence. These findings have important implications for public health efforts on how to enhance compliance since they provide information about which populations are likely to have less breast cancer screening adherence. Periodic reviews of the participation of women of diverse origins and socioeconomic groups in preventative programs should be conducted by public health services. Moreover, it would be better if awareness was raised among this population of the need for disease prevention by extra reminders, such as mail or phone. Finally, public health agencies should examine ways to increase access to mammography screening, including the use of mobile screening units [75].

## Figures and Tables

**Table 1:** Uptake of mammography according to socio-demographic characteristics, health-related status, and lifestyle behaviors (N = 7220).

Variables	Uptake of Mammography			
	Total n (%)	Yes n = 5635 (%)	No n = 1585 (%)	*p*-Value
Age group				0.22
50–56 years old	2667 (36.94)	2083 (78.10)	584 (21.90)	
57–63 years old	2570 (35.60)	2029 (78.95)	541 (21.05)	
64–69 years old	1983 (27.47)	1523 (76.80)	460 (23.20)	
Educational level				<0.001 ***
Without studies	495 (6.86)	352 (71.11)	143 (28.89)	
Primary	1507 (20.87)	1145 (75.98)	362 (24.02)	
Secondary	3808 (52.74)	3000 (78.78)	808 (21.22)	
University	1410 (19.53)	1138 (80.71)	272 (19.29)	
Marital status				<0.001 ***
Single	922 (12.77)	658 (71.37)	264 (28.63)	
Married	4420 (61.22)	3554 (80.41)	866 (19.59)	
Widowed	875 (12.12)	685 (78.29)	190 (21.71)	
Separated or divorced	1003 (13.89)	738 (73.58)	265 (26.42)	
Social class				<0.01 **
Lower	3401 (47.11)	2600 (76.45)	801 (23.55)	
Middle	2441 (33.81)	1927 (78.94)	514 (21.06)	
Upper	1378 (19.09)	1108 (80.41)	270 (19.59)	
Residential location				<0.001 ***
Urban	3163 (43.81)	2403 (75.97)	760 (24.03)	
Rural	4057 (56.19)	3232 (79.66)	825 (20.34)	
Nationality				<0.001 ***
Spanish	6929 (95.97)	5486 (78.91)	124 (42.61)	
Foreigner	291 (4.03)	167 (57.39)	1461 (21.09)	
Number of chronic conditions				<0.01 **
0	2815 (38.99)	2142 (76.09)	673 (23.91)	
1	2287 (31.68)	1806 (78.97)	481 (21.03)	
≥2	2118 (29.34)	1687 (79.65)	431 (20.35)	
Mental illness				0.14
No	5666 (78.48)	4401 (77.67)	1265 (22.33)	
Yes	1554 (21.52)	1234 (79.41)	320 (20.59)	
Self-perceived health status				0.53
Very good	925 (12.81)	719 (77.73)	206 (22.27)	
Good	3693 (51.15)	2896 (78.42)	797 (21.58)	
Fair	1892 (26.20)	1482 (78.33)	410 (21.67)	
Poor	543 (7.52)	415 (76.43)	128 (23.57)	
Very poor	167 (2.31)	123 (73.65)	44 (26.35)	
Type of healthcare insurance				0.03 *
Public	6840 (5.26)	5321 (77.79)	1519 (22.21)	
Private	380 (5.26)	314 (82.63)	66 (17.37)	
Visits to the primary care physician in the previous 4 weeks				0.01 *
No	4993 (69.16)	3856 (77.23)	1137 (22.77)	
Yes	2227 (30.84)	1779 (79.88)	448 (20.12)	
Body mass index				0.03 *
Normal weight	3146 (43.57)	2476 (78.70)	670 (21.30)	
Underweight	131 (1.81)	89 (67.94)	42 (32.06)	
Overweight	2589 (35.86)	2021 (78.06)	568 (21.94)	
Obesity	1354 (18.75)	1049 (77.47)	305 (22.53)	
Smoking status				<0.001 ***
Never smoker	3695 (51.18)	2871 (77.70)	824 (22.30)	
Former smoker	1831 (25.36)	1497 (81.76)	334 (18.24)	
Current smoker	1694 (23.46)	1267 (74.79)	427 (25.21)	
Alcohol use				<0.001 ***
Never	1732 (23.99)	1307 (75.46)	425 (24.54)	
Former	1128 (15.62)	823 (72.96)	305 (27.04)	
Current	4360 (60.39)	3505 (80.39)	855 (19.61)	
Leisure-time physical activity				<0.001 ***
Non	2446 (33.88)	1802 (73.67)	644 (26.33)	
Lower	2937 (40.68)	2353 (80.12)	584 (19.88)	
Higher	1837 (25.44)	1480 (80.57)	357 (19.43)	

Statistical test used: Chi-squared test. Significance at the level: * *p* < 0.05; ** *p* < 0.01; *** *p* < 0.001.

**Table 2:** Distribution of uptakers of mammography, according to sociodemographic, health, and lifestyle variables from 2017 to 2020 (n = 5635).

Variables	Mammography Screening (n = 5635)		
	2017 n = 3013 n (%)	2020 n = 2622 n (%)	*p*-Value
Age group			
50–56 years old	1140 (80.91)	943 (74.96)	<0.001 ***
57–63 years old	1061 (81.99)	968 (75.86)	<0.001 ***
64–69 years old	812 (80.72)	711 (72.77)	<0.001 ***
Educational level			
Without studies	246 (77.12)	106 (60.23)	<0.001 ***
Primary	671 (80.26)	474 (70.64)	<0.001 ***
Secondary	1554 (81.96)	1446 (75.63)	<0.001 ***
University	542 (82.37)	596 (79.26)	0.14
Marital status			
Single	313 (72.29)	345 (70.55)	0.61
Married	1959 (83.33)	1595 (77.09)	<0.001 ***
Widowed	392 (83.94)	293 (71.81)	<0.001 ***
Separated or divorced	349 (76.20)	389 (71.38)	0.09
Social class			
Lower	1453 (80.63)	1147 (71.73)	<0.001 ***
Middle	1022 (81.89)	905 (75.86)	<0.001 ***
Upper	538 (81.64)	570 (79.28)	0.27
Residential location			
Urban	1257 (79.41)	1146 (72.53)	<0.001 ***
Rural	1756 (82.60)	1476 (76.44)	<0.001 ***
Nationality			
Spanish	2914 (82.11)	2554 (75.56)	<0.001 ***
Foreigner	99 (61.88)	68 (51.91)	0.10
Number of chronic conditions			
0	1108 (80.23)	1034 (72.11)	<0.001 ***
1	934 (80.87)	872 (77.03)	0.02 *
≥2	971 (82.78)	716 (75.77)	<0.001 ***
Mental illness			
No	2280 (81.02)	2121 (74.37)	<0.001 ***
Yes	733 (81.90)	501 (76.02)	<0.01 **
Self-perceived health status			
Very good	339 (78.47)	380 (77.08)	<0.001 ***
Good	1505 (82.24)	1391 (74.66)	<0.001 ***
Fair	845 (81.72)	637 (74.24)	<0.001 ***
Poor	249 (79.81)	166 (71.86)	0.03 *
Very poor	75 (74.26)	48 (72.73)	0.83
Type of healthcare insurance			
Public	2864 (81.23)	2457 (74.14)	<0.001 ***
Private	149 (81.42)	165 (83.76)	<0.001 ***
Visits to the primary care physician in the previous 4 weeks			
No	1945 (80.57)	1911 (74.10)	<0.001 ***
Yes	1068 (82.47)	711 (76.29)	<0.001 ***
Body mass index			
Normal weight	1277 (81.65)	1199 (75.79)	<0.001 ***
Underweight	46 (68.66)	43 (67.19)	0.86
Overweight	1101 (82.29)	920 (73.54)	<0.001 ***
Obesity	589 (79.59)	460 (74.92)	0.04 *
Smoking status			
Never smoker	1556 (81.98)	1315 (73.18)	<0.001 ***
Former smoker	771 (83.53)	726 (79.96)	0.04 *
Current smoker	686 (77.25)	581 (72.08)	0.01 *
Alcohol use			
Never	851 (77.36)	456 (72.15)	<0.001 ***
Former	317 (81.28)	506 (68.56)	<0.001 ***
Current	1845 (83.15)	1660 (77.53)	<0.001 ***
Leisure-time physical activity			
Non	986 (77.33)	816 (69.68)	<0.001 ***
Lower	1209 (82.41)	1806 (77.82)	<0.001 ***
Higher	818 (84.59)	662 (76.09)	<0.001 ***

Statistical test used: Chi-squared test. Significance at the level: * *p* < 0.05; ** *p* < 0.01; *** *p* < 0.001.

**Table 3:** Variables associated with adherence to mammography screening among women residing in Spain stratified by year (2017 and 2020).

Variables		2017				2020		
	OR (CI 95%)	B	ORa (95% CI)	*p*-Value	OR (CI 95%)	B	ORa (95% CI)	*p*-Value
Age group								
50–56 years old	Reference				Reference			
57–63 years old	1.08 (0.89–1.31)				1.05 (0.88–1.26)			
64–69 years old	0.91 (0.80–1.21)				0.89 (0.74–1.08)			
Educational level								
Without studies	Reference		Reference		Reference		Reference	
Primary	1.21 (0.88–1.65)	0.17	1.18 (0.86–1.63)	0.30	1.59 (1.13–2.24)	0.38	1.46 (0.48–2.07)	0.31
Secondary	1.35 (1.01–1.80)	0.32	1.37 (1.01–1.85)	0.04	2.05 (1.49–2.82)	0.60	1.82 (1.29–2.54)	<0.01
University	1.39 (0.99–1.93)	0.37	1.45 (1.02–2.07)	0.04	2.52 (1.78–3.58)	0.75	2.12 (1.44–3.09)	<0.001
Marital status								
Single	Reference		Reference		Reference		Reference	
Married	1.92 (1.51–2.43)	0.62	1.86 (1.46–2.38)	<0.001	1.41 (1.13–1.75)	0.34	1.41 (1.12–1.77)	<0.01
Widowed	2.00 (1.45–2.77)	0.75	2.11 (0.89–2.96)	0.26	1.06 (0.80–1.42)	0.15	1.16 (0.85–1.57)	0.17
Separated or divorced	1.23 (0.91–1.66)	0.27	1.31 (0.96–1.77)	0.10	1.04 (0.80–1.36)	0.05	1.05 (0.80–1.39)	0.74
Social class								
Lower	Reference				Reference			
Middle	1.09 (0.90–1.31)				1.24 (1.04–1.47)			
Upper	1.07 (0.85–1.34)				1.51 (1.22–1.86)			
Residential location								
Urban	Reference		Reference		Reference		Reference	
Rural	0.81 (0.69–0.96)	−0.17	0.84 (0.71–0.93)	<0.01	0.81 (0.70–0.95)	−0.22	0.80 (0.68–0.94)	<0.01
Nationality								
Spanish	Reference		Reference		Reference		Reference	
Foreigner	0.35 (0.25–0.49)	−0.99	0.37 (0.26–0.52)	<0.001	0.35 (0.25–0.50)	−0.91	0.40 (0.28–0.57)	<0.001
Number of chronic conditions								
0	Reference				Reference		Reference	
1	1.04 (0.86–1.27)				1.30 (1.08–1.55)	0.30	1.35 (1.13–1.63)	<0.01
≥2	1.18 (0.97–1.45)				2.21 (1.01–1.46)	0.33	1.39 (1.14–1.70)	<0.01
Mental illness								
No	Reference				Reference			
Yes	1.06 (0.87–1.29)				1.09 (0.90–1.33)			
Self-perceived health status								
Very good	Reference				Reference			
Good	1.27 (0.98–1.65)				1.26 (0.71–2.26)			
Fair	1.23 (0.93–1.62)				1.11 (0.64–1.92)			
Poor	1.08 (0.76–1.55)				1.08 (0.62–1.90)			
Very poor	0.79 (0.48–1.319				0.96 (0.52–1.77)			
Type of healthcare insurance								
Public	Reference				Reference		Reference	
Private	1.01 (0.69–1.48)				1.80 (1.22–2.65)	0.45	1.56 (1.05–2.34)	0.03
Visits to the primary care physician in the previous 4 weeks								
No	Reference				Reference			
Yes	1.13 (0.95–1.35)				1.13 (0.94–1.34)			
Body mass index								
Normal weight	Reference				Reference			
Underweight	0.49 (0.29–0.84)				0.65 (0.38–1.12)			
Overweight	1.04 (0.86–1.26)				0.89 (0.75–1.05)			
Obesity	0.88 (0.70–1.10)				0.95 (0.77–1.18)			
Smoking status								
Never smoker	Reference		Reference		Reference		Reference	
Former smoker	1.12 (0.90–1.38)	0.02	1.02 (0.81–1.26)	0.81	1.46 (0.80–1.77)	0.22	1.25 (0.73–1.53)	0.12
Current smoker	0.75 (0.61–0.91)	−0.32	0.73 (0.59–0.89)	<0.01	0.95 (0.79–1.14)	−0.11	0.90 (0.74–0.93)	0.03
Alcohol use								
Never	Reference		Reference		Reference		Reference	
Former	0.68 (0.41–1.11)	−0.33	0.72 (0.59–1.13)	0.11	0.84 (0.67–1.06)	−0.19	0.83 (0.65–1.07)	0.14
Current	1.27 (1.14–1.64)	0.26	1.30 (1.19–1.75)	<0.01	1.29 (1.08–1.63)	0.16	1.18 (1.10–1.52)	<0.01
Leisure-time physical activity								
Non	Reference		Reference		Reference		Reference	
Lower	1.37 (1.14–1.66)	0.19	1.20 (1.10–1.58)	<0.01	1.39 (1.13–1.69)	0.18	1.20 (1.08–1.54)	<0.01
Higher	1.61 (1.29–2.01)	0.38	1.46 (1.16–1.82)	<0.01	1.53 (1.28–1.82)	0.32	1.38 (1.15–1.65)	<0.01

OR, odds ratio; ORa, odds ratio adjusted for all socio-demographic characteristics, health-related status, and lifestyle behaviors; 95% CI, 95% confidence interval. B, regression coefficient. 2017: Constant = 0.508; Hosmer–Lemeshow test χ^2^ = 13.67, *p* = 0.09; −2 log likelihood for the intercept = 3581.409, −2 log likelihood for the final model = 3461.485, −2 log likelihood χ^2^ = 119.925, *p*-value < 0.001; Nagelkerke’s R^2^ square = 0.38; *p*-value < 0.001. 2020: Constant = −0.004; Hosmer–Lemeshow test χ^2^ = 7.68, *p* = 0.47; −2 log likelihood for the intercept = 3973.244, −2 log likelihood for the final model = 3831.468, −2 log likelihood χ^2^ = 141.777, *p*-value < 0.001; Nagelkerke’s R^2^ square = 0.43; *p*-value < 0.001.

**Table 4:** Variables associated with adherence to mammography screening among women residing in Spain during 2017 and 2020.

Variables	OR (95%CI)	B	ORa (95% CI)	*p*-Value
Year				
2020	Reference		Reference	
2017	1.47 (1.31–1.64)	0.39	1.48 (1.32–1.67)	<0.001
Age group				
50–56 years old	Reference			
57–63 years old	1.05 (0.92–1.20)			
64–69 years old	0.93 (0.81–1.07)			
Educational level				
Without studies	Reference		Reference	
Primary	1.29 (1.02–1.61)	0.26	1.29 (0.98–1.63)	0.06
Secondary	1.51 (1.22–1.86)	0.45	1.57 (1.26–1.89)	<0.001
University	1.70 (1.34–2.15)	0.61	1.84 (1.37–2.27)	<0.001
Marital status				
Single	Reference		Reference	
Married	1.65 (1.40–1.93)	0.47	1.60 (1.36–1.90)	<0.001
Widowed	1.45 (0.89–1.33)	0.42	1.52 (0.93–1.42)	0.24
Separated or divorced	1.12 (0.92–1.37)	0.14	1.15 (0.73–1.14)	0.17
Social class				
Lower	Reference			
Middle	1.16 (1.02–1.31)			
Upper	1.26 (1.08–1.48)			
Residential location				
Urban	Reference		Reference	
Rural	0.81 (0.72–0.90)	−0.20	0.82 (0.73–0.92)	<0.01
Nationality				
Spanish	Reference		Reference	
Foreigner	0.36 (0.28–0.46)	−0.97	0.38 (0.30–0.49)	<0.001
Number of chronic conditions				
0	Reference		Reference	
1	1.18 (1.03–1.35)	0.18	1.20 (1.04–1.37)	0.01
≥2	1.23 (1.07–1.41)	0.26	1.30 (1.13–1.50)	<0.01
Mental illness				
No	Reference			
Yes	1.11 (0.97–1.28)			
Self-perceived health status				
Very good	Reference			
Good	1.04 (0.88–1.24)			
Fair	1.04 (0.86–1.25)			
Poor	0.93 (0.72–1.20)			
Very poor	0.80 (0.55–1.17)			
Type of healthcare insurance				
Public	Reference			
Private	1.36 (1.04–1.78)			
Visits to the primary care physician in the previous 4 weeks				
No	Reference		Reference	
Yes	1.17 (1.04–1.32)	0.12	1.13 (1.05–1.35)	0.02
Body mass index				
Normal weight	Reference			
Underweight	0.57 (0.39–0.84)			
Overweight	0.96 (0.85–1.09)			
Obesity	0.93 (0.80–1.09)			
Smoking status				
Never smoker	Reference		Reference	
Former smoker	1.15 (0.82–1.26)	0.12	1.13 (0.97–1.30)	0.12
Current smoker	0.79 (0.69–0.89)	−0.20	0.82 (0.73–0.92)	0.02
Alcohol use				
Never	Reference		Reference	
Former	0.88 (0.74–1.04)	−0.02	0.98 (0.82–1.18)	0.13
Current	1.33 (1.17–1.52)	0.29	1.34 (1.19–1.51)	<0.001
Leisure-time physical activity				
Non	Reference		Reference	
Lower	1.44 (1.27–1.64)	0.27	1.31 (1.15–1.50)	<0.001
Higher	1.48 (1.28–1.72)	0.29	1.34 (1.15–1.56)	<0.001

OR, odds ratio; ORa, odds ratio adjusted for all socio-demographic characteristics, health-related status, and lifestyle behaviors; 95% IC, 95% confidence interval; B, regression coefficient. Constant = −0.025; Hosmer–Lemeshow test χ^2^ = 2.21, *p* = 0.11; −2 log likelihood for the intercept = 7599.935, −2 log likelihood for the final model = 7314.203, −2 log likelihood χ^2^ = 285.732, *p*-value < 0.001; Nagelkerke’s R^2^ square = 0.33; *p*-value < 0.001.

## Data Availability

The data presented in this study are available as Supplementary Material (File S1: Research data).

## Supplementary Materials

The following supporting information can be downloaded at: https://www.mdpi.com/article/10.3390/healthcare11222934/s1, File S1: Research data.

## References

[1] Arnold M., Morgan E., Rumgay H., Mafra A., Singh D., Laversanne M., Vignat J., Gralow J.R., Cardoso F., Siesling S. (2022). Current and future burden of breast cancer: Global statistics for 2020 and 2040. Breast.

[2] European Commission (2023). Breast Cancer in the EU.

[3] European Commission (2022). Cancer Information System.

[4] Hadadi I., Rae W., Clarke J., McEntee M., Ekpo E. (2021). Breast Cancer Detection: Comparison of Digital Mammography and Digital Breast Tomosynthesis across Non-Dense and Dense Breasts. Radiography.

[5] Taylor R., Gregory M., Sexton K., Wharton J., Sharma N., Amoyal G., Morrell S. (2019). Breast cancer mortality and screening mammography in New Zealand: Incidence-based and aggregate analyses. J. Med. Screen..

[6] Bevers T.B., Helvie M., Bonaccio E., Calhoun K.E., Daly M.B., Farrar W.B., Garber J.E., Gray R., Greenberg C.C., Greenup R. (2018). Breast Cancer Screening and Diagnosis, Version 3.2018, NCCN Clinical Practice Guidelines in Oncology. J. Natl. Compr. Canc. Netw..

[7] Moller M.H., Lousdal M.L., Kristiansen I.S., Stovring H. (2019). Effect of organized mammography screening on breast cancer mortality: A population-based cohort study in Norway. Int. J. Cancer.

[8] Dibden A., Offman J., Duffy S.W., Gabe R. (2020). Worldwide Review and Meta-Analysis of Cohort Studies Measuring the Effect of Mammography Screening Programmes on Incidence-Based Breast Cancer Mortality. Cancers.

[9] Sardanelli F., Fallenberg E.M., Clauser P., Trimboli R.M., Camps-Herrero J., Helbich T.H., Forrai G., European Society of Breast Imaging (EUSOBI) (2017). The European Breast Cancer Coalition Mammography: An Update of the EUSOBI recommendations on information for women. Insights Imaging.

[10] Peintinger F. (2019). National breast screening programs across Europe. Breast Care.

[11] Martín-López R., Jiménez-García R., Lopez-de-Andres A., Hernández-Barrera V., Jiménez-Trujillo I., Gil-de-Miguel A., Carrasco-Garrido P. (2013). Inequalities in uptake of breast cancer screening in Spain: Analysis of a Cross-Sectional National Survey. Public Health.

[12] Saz-Parkinson Z., Monteagudo-Piqueras O., Granados Ortega J., Martínez Mondéjar E., Labrador Cañadas M.V. (2020). “European Commission Initiative on Breast Cancer”: Selected breast cancer screening recommendations from the European Guidelines. Rev. Esp. Salud Publica.

[13] Schünemann H.J., Lerda D., Quinn C., Follmann M., Alonso-Coello P., Rossi P.G., Lebeau A., Nyström L., Broeders M., Ioannidou-Mouzaka L. (2020). Breast cancer screening and diagnosis: A Synopsis of the European Breast Guidelines. Ann. Intern. Med..

[14] Zielonke N., Kregting L.M., Heijnsdijk E.A.M., Veerus P., Heinävaara S., McKee M., de Kok I.M.C.M., de Koning H.J., van Ravesteyn N.T., EU-TOPIA collaborators (2021). The potential of breast cancer screening in Europe. Int. J. Cancer.

[15] Zamorano-Leon J.J., López-de-Andres A., Álvarez-González A., Astasio-Arbiza P., López-Farré A.J., de-Miguel-Diez J., Jiménez-García R. (2020). Reduction from 2011 to 2017 in adherence to breast cancer screening and non-improvement in the uptake of cervical cancer screening among women living in Spain. Maturitas.

[16] Carmona-Torres J.M., Cobo-Cuenca A.I., Martín-Espinosa N.M., Piriz-Campos R.M., Laredo-Aguilera J.A., Rodríguez-Borrego M.A. (2018). Prevalence in the performance of mammographies in Spain: Analysis by Communities 2006-2014 and influencing factors. Aten. Primaria.

[17] Moreira C.B., Dahinten V.S., Howard A.F., Fernandes A.F.C., Schirmer J. (2021). Factors related to mammography adherence among women in Brazil: A Scoping review. Nurs. Open.

[18] Willems B., Bracke P. (2018). The education gradient in cancer screening participation: A consistent phenomenon across Europe?. Int. J. Public Health.

[19] Bhargava S., Moen K., Qureshi S.A., Hofvind S. (2018). Mammographic screening attendance among immigrant and minority women: A systematic review and meta-analysis. Acta Radiol..

[20] Satoh M., Sato N. (2021). Relationship of attitudes toward uncertainty and preventive health behaviors with breast cancer screening participation. BMC Womens Health.

[21] Anwar S.L., Tampubolon G., Van Hemelrijck M., Hutajulu S.H., Watkins J., Wulaningsih W. (2018). Determinants of cancer screening awareness and participation among Indonesian women. BMC Cancer.

[22] Ministry of Health, Consumer Affairs and Social Welfare, National Institute of Statistics (2017). Encuesta Nacional de Salud España ENSE 2017.

[23] Ministry of Health, Consumer Affairs and Social Welfare, National Institute of Statistics (2020). Encuesta Europea de Salud en España 2020.

[24] Ministry of Health, Consumer Affairs and Social Welfare (2022). Cribado Poblacional del Cáncer de Mama.

[25] International Labour Office (2018). Rural-Urban Labour Statistics.

[26] Domingo-Salvany A., Bacigalupe A., Carrasco J.M., Espelt A., Ferrando J., Borrell C. (2013). Proposals for social class classification based on the Spanish National Classification of Occupations 2011 using neo-Weberian and neo-Marxist approaches 2011. Gac. Sanit..

[27] World Health Organization (WHO) Body Mass Index (BMI). http://www.euro.who.int/en/health-topics/disease-prevention/nutrition/a-healthy-lifestyle/body-mass-index-bmi.

[28] Winship C., Radbill L. (1994). Sampling weights and regression analysis. Sociol. Methods. Res..

[29] International Agency for Research on Cancer (2017). Cancer Screening in the European Union, Report on the Implementation of the Council Recommendation on Cancer Screening.

[30] Jani C., Salcicciol I., Rupal A., Al Omari O., Goodall R., Salciccioli J.D., Marshall D.C., Hanbury G., Singh H., Weissmann L. (2021). Trends in Breast Cancer Mortality between 2001 and 2017: An Observational Study in the European Union and the United Kingdom. JCO Glob. Oncol..

[31] Brugel M., Carlier C., Essner C., Debreuve-Theresette A., Beck M.-F., Merrouche Y., Bouché O. (2021). Dramatic changes in oncology care pathways during the COVID-19 pandemic: The French ONCOCARE-COV Study. Oncologist.

[32] Fedewa S.A., Cotter M.M., Wehling K.A., Wysocki K., Killewald R., Makaroff L. (2021). Changes in breast cancer screening rates among 32 community health centers during the COVID-19 pandemic. Cancer.

[33] Peng S.-M., Yang K.-C., Chan W.P., Wang Y.-W., Lin L.-J., Yen A.M.-F., Smith R.A., Chen T.H.-H. (2020). Impact of the COVID-19 pandemic on a population-based breast cancer screening program: COVID-19 and mammography screening. Cancer.

[34] London J.W., Fazio-Eynullayeva E., Palchuk M.B., McNair C. (2022). Evolving effect of the COVID-19 pandemic on cancer-related encounters. JCO Clin. Cancer Inform..

[35] Li T., Nickel B., Ngo P., McFadden K., Brennan M., Marinovich M.L., Houssami N. (2023). A systematic review of the impact of the COVID-19 pandemic on breast cancer screening and diagnosis. Breast.

[36] Vanni G., Materazzo M., Pellicciaro M., Ingallinella S., Rho M., Santori F., Cotesta M., Caspi J., Makarova A., Pistolese C.A. (2020). Breast cancer and COVID-19: The effect of fear on patients’ decision-making process. In Vivo.

[37] Bosch G., Posso M., Louro J., Roman M., Porta M., Castells X., Macià F. (2022). Impact of the COVID-19 Pandemic on Breast Cancer Screening Indicators in a Spanish Population-Based Program: A Cohort Study. Elife.

[38] Molina-Barceló A., Moreno Salas J., Peiró-Pérez R., Arroyo G., Ibáñez Cabanell J., Vanaclocha Espí M., Binefa G., García M., Salas Trejo D. (2021). Inequalities in access to cancer screening programmes in Spain and how to reduce them: Data from 2013 and 2020. Rev. Esp. Salud Publica.

[39] Unim B., Boggi R., Napoli M., Fulgenzi R., Landi A., La Torre G. (2020). Predictors of mammography uptake among Italian women aged 50-69: A cross-sectional study. J. Cancer Educ..

[40] Okui T. (2021). Analysis of predictors of breast cancer screening among Japanese women using Nationally Representative Survey Data, 2001–2013. Asian Pac. J. Cancer Prev..

[41] Petrelli A., Giorgi Rossi P., Francovich L., Giordani B., Di Napoli A., Zappa M., Mirisola C., Gargiulo L. (2018). Geographical and socioeconomic differences in uptake of pap test and mammography in Italy: Results from the National Health Interview Survey. BMJ Open.

[42] Pataki J., Dombrádi V., Sárváry A., Szőllősi G.J. (2023). Breast cancer screening and its associating factors among hungarian women aged 45–65: A cross-sectional study based on the European Health Interview Surveys from 2009 to 2019. BMC Public Health.

[43] Willems B., Bracke P. (2018). Participants, physicians or programmes: Participants’ educational level and initiative in cancer screening. Health Policy.

[44] Flores E.J., López D., Miles R.C., Glover M., Lehman C.D., Harvey H.B., Narayan A.K. (2019). Impact of primary care physician interaction on longitudinal adherence to screening mammography across different racial/ethnic groups. J. Am. Coll. Radiol..

[45] Ouanhnon L., Bugat M.-E.R., Druel V., Grosclaude P., Delpierre C. (2023). Link between the referring physician and breast and cervical cancers screening: A cross-sectional study in France. BMC Prim. Care.

[46] Tran L., Tran P. (2019). US urban-rural disparities in breast cancer screening practices at the national, regional, and state level, 2012-2016. Cancer Causes Control.

[47] Theodoropoulos N., Xie H., Wang Q., Wen C., Li Y. (2022). Rural-urban differences in breast and colorectal cancer screening among US women, 2014–2019. Rural Remote Health.

[48] Chandak A., Nayar P., Lin G. (2019). Rural-urban disparities in access to breast cancer screening: A spatial clustering analysis: Disparities in breast cancer screening. J. Rural Health.

[49] Jewett P.I., Gangnon R.E., Elkin E., Hampton J.M., Jacobs E.A., Malecki K., LaGro J., Newcomb P.A., Trentham-Dietz A. (2018). Geographic access to mammography facilities and frequency of mammography screening. Ann. Epidemiol..

[50] Sprague B.L., Ahern T.P., Herschorn S.D., Sowden M., Weaver D.L., Wood M.E. (2021). Identifying key barriers to effective breast cancer control in rural settings. Prev. Med..

[51] Katz D., Tengekyon A.J., Kahan N.R., Calderon-Margalit R. (2018). Patient and physician characteristics affect adherence to screening mammography: A population-based cohort study. PLoS ONE.

[52] Moreira C.B., Fernandes A.F.C., Castro R.C.M.B., de Oliveira R.D.P., Pinheiro A.K.B. (2018). Social determinants of health related to adhesion to mammography screening. Rev. Bras. Enferm..

[53] Mondragón L.I., Domínguez D.L., González L.M., Liu J.J. (2023). Associations between sociodemographic factors and breast, cervical, and colorectal cancer screening in the United States. Cancer Causes Control.

[54] Sakellariou D., Rotarou E.S. (2019). Utilisation of mammography by women with mobility impairment in the UK: Secondary analysis of Cross-Sectional Data. BMJ Open.

[55] Gatewood A., Enmeier M., Stephenson E., Austin J., Greiner B., Hartwell M. (2022). Breast cancer screening among women with comorbidities: A cross-sectional examination of disparities from the behavioral risk factor surveillance system. J. Oncol. Navig. Surviv..

[56] Yuan C., Kulkarni K., Dashevsky B.Z. (2020). Preventive Care: How mammography utilization changes as women age. J. Am. Coll. Radiol..

[57] Beau A.-B., Napolitano G.M., Ewertz M., Vejborg I., Schwartz W., Andersen P.K., Lynge E. (2020). Impact of chronic diseases on effect of breast cancer screening. Cancer Med..

[58] National Institute of Statistics (2022). Cifras de Población. Últimos Datos.

[59] Ferdous M., Goopy S., Yang H., Rumana N., Abedin T., Turin T.C. (2020). Barriers to breast cancer screening among immigrant populations in Canada. J. Immigr. Minor. Health.

[60] March S., Villalonga B., Sanchez-Contador C., Vidal C., Mascaro A., de Lluc Bennasar M., Esteva M. (2018). Barriers to and discourses about breast cancer prevention among immigrant women in Spain: A qualitative study. BMJ Open.

[61] National Institute of Statistics (2020). Encuesta Europea de Salud en España 2020, Asistencia Sanitaria: Cifras Relativas.

[62] National Institute of Statistics (2017). Encuesta Nacional de Salud en España 2017, Asistencia Sanitaria: Cifras Relativas.

[63] García A. (2011). Is Spanish public health sinking?. BMJ.

[64] Tur-Sinai A., Shahrabani S. (2020). Determinants of women’s decision to undergo early mammography: A survey study. Nurs. Health. Sci..

[65] Patrão A.L., de Almeida M.D.C.C., Matos S.M.A., Menezes G., Gabrielli L., Goes E.F., Aquino E.M. (2021). Healthy lifestyle behaviors and the periodicity of mammography screening in Brazilian women. Womens Health.

[66] Lu N., Zhang C., You H., Ma Z., Zhu P., Cheng F. (2022). Factors affecting breast screening behavior of first-degree relatives of breast cancer patients in China: A cross-sectional study. Cancer Nurs..

[67] Eng V.A., David S.P., Li S., Ally M.S., Stefanick M., Tang J.Y. (2020). the association between cigarette smoking, cancer screening, and cancer stage: A prospective study of the women’s health initiative observational cohort. BMJ Open.

[68] Wu S., Liang D., Shi J., Li D., Liu Y., Hao Y., Shi M., Du X., He Y. (2023). Evaluation of a population-based breast cancer screening in North China. J. Cancer Res. Clin. Oncol..

[69] Bui N.C., Lee Y.Y., Suh M., Park B., Cho H., Kim Y., Choi K.S. (2018). Beliefs and intentions to undergo lung cancer screening among Korean males. Cancer Res. Treat..

[70] Baumeister R.F. (2017). Addiction, Cigarette smoking, and voluntary control of action: Do cigarette smokers lose their free will?. Addict. Behav. Rep..

[71] Mu L., Mukamal K.J. (2016). Alcohol consumption and rates of cancer screening: Is cancer risk overestimated?. Cancer Causes Control.

[72] Carey R.N., El-Zaemey S. (2020). Lifestyle and occupational factors associated with participation in breast mammography screening among Western Australian women. J. Med. Screen..

[73] Huang C.-H., Lo Y.-J., Kuo K.-M., Lu I.-C., Wu H., Hsieh M.-T., Liu I.-T., Lin Y.-C., Lai Y.-C., Huang R.-Y. (2021). Health literacy and cancer screening behaviors among community-dwelling female adults in Taiwan. Women Health.

[74] Kriaucioniene V., Petkeviciene J. (2019). Predictors and trend in attendance for breast cancer screening in Lithuania, 2006–2014. Int. J. Environ. Res. Public Health.

[75] Agide F.D., Sadeghi R., Garmaroudi G., Tigabu B.M. (2018). A systematic review of health promotion interventions to increase breast cancer screening uptake: From the last 12 Years. Eur. J. Public Health.

