# Supplementary material for: Temporal Evolution and Associated Factors of Adherence to Mammography Screening among Women in Spain: Results from Two National Health Surveys (2017–2020)
Source: Healthcare (Basel). 2023 Nov 9;11(22):2934. doi: 10.3390/healthcare11222934 (PMC10671473; doi:10.3390/healthcare11222934)
Supplement: Supplementary file 1 [file healthcare-11-02934-s001.zip › Questionnaire European Health Survey for Spain 2020.pdf]

ENCUESTA EUROPEA DE SALUD EN ESPAÑA 2020

CUESTIONARIO INDIVIDUAL

Proxy_0. ¿El informante es la persona seleccionada?

Sí ▢ 1 → Ir a Introducción P.1
No ▢ 2

Proxy_1. ¿Cuál es el motivo por el que la persona seleccionada no puede facilitar personalmente sus datos?

La persona seleccionada está ingresada en un sanatorio, hospital, etc., a causa de una enfermedad ___ ▢ 1
La persona seleccionada está incapacitada para contestar por discapacidad, enfermedad grave, etc. ___ ▢ 2
La persona seleccionada está incapacitada para contestar por causa del idioma ___ ▢ 3

Proxy_2. ¿Es miembro del hogar el informante?

Sí ___ ▢ 1 → Listado de miembros del hogar: | _ | (proxy_2b) → Ir a Proxy_5
No ___ ▢ 2

Proxy_3. Nombre del informante: ___

Proxy_3b. Sexo del informante: ▢ 1 Hombre ▢ 2 Mujer

Proxy_4. Edad del informante: |_|_|

Proxy_5. ¿Cuál es la relación del informante con la persona seleccionada?

Cónyuge o pareja ______________________ ☐ 1
Hijo/Hija______________________________ ☐ 2
Padre/madre ___________________________ ☐ 3
Hermano/hermana _______________________ ☐ 4
Otros familiares_______________________ ☐ 5
Servicios sociales _____________________ ☐ 6
Voluntarios ___________________________ ☐ 7
Otra relación __________________________ ☐ 8

E.- CARACTERÍSTICAS DEMOGRÁFICAS DE LA PERSONA ADULTA SELECCIONADA

Introducción P.1: Entrevistador/a, léale al informante: **"A continuación voy a hacerle unas preguntas sobre su nacionalidad y su estado civil".**

1. ¿Cuál es su país de nacimiento?

España __________________ ☐ 1
Extranjero ________________ ☐ 2 → **1b-** Seleccionar país
No sabe _________________ ☐ 8
No contesta______________ ☐ 9

1c. ¿Cuál es el país de nacimiento de su madre?

España__________________ ☐ 1
Extranjero _______________ ☐ 2 → **1d-** Seleccionar país
No sabe _________________ ☐ 8
No contesta ______________ ☐ 9

1e. ¿Cuál es el país de nacimiento de su padre?

España__________________ ☐ 1
Extranjero _______________ ☐ 2 → **1f-** Seleccionar país
No sabe _________________ ☐ 8
No contesta ______________ ☐ 9

2. ¿Y cuál es su nacionalidad?

Española ☐
Extranjera ☐ → **2b-** Seleccionar país
No sabe ☐
No contesta ☐

Nota P.2: Se admite respuesta múltiple.

Si P.1 = 1 → Ir a P.4

3. ¿Cuántos años lleva residiendo en España?

Nº de años ⌴⌴ NS ☐ 98 NC ☐ 99

4. De las siguientes opciones, ¿cuál describe su situación actual?

Conviviendo con su cónyuge ☐ 1
Conviviendo con una pareja de hecho ☐ 2
No conviviendo en pareja ☐ 3
No sabe ☐ 8
No contesta ☐ 9

4b. ¿Cuál es su estado civil legal?

Soltero/a ☐ 1
Casado/a ☐ 2
Viudo/a ☐ 3
Separado/a legalmente ☐ 4
Divorciado/a ☐ 5
No sabe ☐ 8
No contesta ☐ 9

5. Por favor, detalle a continuación cuál es el mayor nivel de estudios que ha obtenido: ________________________

Entrevistador/a, codifique el nivel de estudios declarado por el adulto eleccionado en el literal anterior:

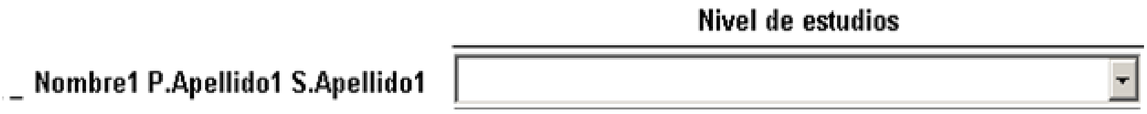

Las opciones que aparecen en el desplegable son:

No sabe leer o escribir (analfabetos)______ 01
Estudios primarios incompletos (ha asistido menos de 5 años a la escuela) 02
Educación primaria completa______ 10
Primera etapa de educación secundaria sin título de graduado en ESO y equivalentes (EGB, Bachillerato elemental)______ 21
Primera etapa de educación secundaria con título de graduado en ESO y similares (EGB, Bachillerato elemental)______ 22
Certificados de profesionalidad de nivel 1 y similares______ 23
Certificados de profesionalidad de nivel 2 y similares______ 24
Bachillerato y similares (BUP, COU, PREU)______ 32
Enseñanzas de formación profesional de grado medio (incluye artes plásticas, diseño, deportivas y similares)______33
Enseñanzas profesionales de música y danza y similares______ 34
Certificado de las escuelas oficiales de idiomas de nivel avanzado y similares______ 35
Formación profesional básica______ 38
Certificados de profesionalidad de nivel 3; programas cortos que requieren segunda etapa de secundaria y similares______ 41
Enseñanzas de formación profesional de grado superior (incluye artes plásticas, diseño, deportivas y equivalentes)______ 51
Títulos propios universitarios que precisan del título de bachiller, de duración igual o superior a 2 años______ 52
Grados universitarios de 240 créditos ECTS (plan Bolonia) y equivalentes___ 61
Diplomados universitarios y equivalentes______ 62
Títulos propios universitarios de experto o especialista de menos de 60 créditos ECTS (plan Bolonia) cuyo acceso requiera ser titulado universitario_ 63
Grados universitarios de más de 240 créditos ECTS (plan Bolonia) y equivalentes ______ 71
Licenciados universitarios y equivalentes______ 72
Másteres oficiales universitarios y equivalentes______ 73
Especialidades en Ciencias de la Salud por el sistema de residencia o similares (MIR y equivalentes)______ 74
Títulos propios universitarios de máster (maestrías) de 60 o más créditos ECTS (plan Bolonia) cuyo acceso requiera ser titulado universitario________ 75
Doctorado universitario______ 81
No contesta______ 99

F.- RELACIÓN DE LA PERSONA ADULTA SELECCIONADA CON LA ACTIVIDAD ECONÓMICA

Introducción P.6: Entrevistador/a, léale al informante: **“Ahora voy a hacerle unas preguntas sobre su relación con la actividad económica”.**

Los flujos de este módulo están condicionados a la respuesta de la pregunta 11 del Cuestionario de Hogar para la persona seleccionada.

Si P.11 (C. Hogar) = 1 → Ir a P.13
Si P.11 (C. Hogar) = 2 → Ir a P.11
Si P.11 (C. Hogar) = 4, 6 ó 7 → Ir a P.12
Si P.11 (C. Hogar) = 3 ó 5 → Ir a P.6
Si P.11 (C. Hogar) = 8 → Ir a Módulo G

6. ¿Percibe alguna pensión contributiva?

Sí, por cotización propia ________________ ☐ 1 → Ir a P.18b
Sí, por cotización de otra persona _________ ☐ 2 → Ir a P.8
Sí, por ambos tipos de cotización _________ ☐ 3 → Ir a P.18b
No ___________________________________ ☐ 4
No sabe ______________________________ ☐ 8
No contesta ___________________________ ☐ 9

7. ¿Ha trabajado antes?

Sí ______________ ☐ 1
No ______________ ☐ 2
No sabe ____________ ☐ 8
No contesta _________ ☐ 9

Si P.7 = 1 y P.6 = 4, 8 ó 9 → Ir a P.18b
Si P.7 = 2, 8 ó 9 y P.6 = 4, 8 ó 9 → Ir a Módulo G

8. ¿Cuál era la actividad de la empresa u organización en la que trabajó en su último empleo la persona que generó la pensión?

Actividad ---

Código de actividad └─┴─┴─┘

9. ¿Cuál era la ocupación, profesión u oficio que desempeñó en su último empleo la persona que generó la pensión?

Profesión ---

Código de ocupación └─┴─┴─┘

10. ¿Cuál era la situación profesional, en su último empleo, de la persona que generó la pensión?

Asalariado/a (a sueldo, comisión, jornal...) ________________ ☐ 1
Empresario/a o profesional con asalariados _______________ ☐ 2
Empresario/a sin asalariados o trabajador/a independiente __ ☐ 3
Ayuda familiar (sin remuneración reglamentada en la empresa o negocio de un familiar) __________________________________ ☐ 4
Miembro de una cooperativa ___________________________ ☐ 5
Otra situación ____ ☐ 6
No sabe ____ ☐ 8
No contesta ____ ☐ 9

Ir a Módulo G

11. ¿Cuánto tiempo lleva en desempleo?

No ha trabajado nunca ___________________ ☐ 1 → Ir a Módulo G
Menos de 6 meses ______________________ ☐ 2 → Ir a P.18b
De 6 meses a menos de 1 año ___________ ☐ 3 → Ir a P.18b
De 1 año a menos de 2 años ______________ ☐ 4 → Ir a P.18b
Más de 2 años _________________________ ☐ 5 → Ir a P.18b
No sabe _____________________________ ☐ 8 → Ir a P.18b
No contesta ___________________________ ☐ 9 → Ir a P.18b

12. Aunque actualmente no esté trabajando, ¿ha trabajado alguna vez anteriormente?

Sí ____________ ▢ 1 → Ir a P.18b
No ______________ ▢ 2 → Ir a Módulo G
No sabe ___________ ▢ 8 → Ir a Módulo G
No contesta _________ ▢ 9 → Ir a Módulo G

13. ¿Qué tipo de contrato o relación laboral tiene?

- Asalariado/a (a sueldo, comisión, jornal...) _________ ▢

Señale el tipo de contrato:
Funcionario/a ___________ ▢ 01 → Ir a P.15
Duración indefinida______ ▢ 02 → Ir a P.15
Temporal ______________ ▢ 03 → Ir a P.14a
Verbal o sin contrato _____ ▢ 04 → Ir a P.14b

- Empresario/a o profesional con asalariados _________ ▢ 05 → Ir a P.15
- Empresario/a sin asalariados o trabajador/a independiente ________________________________ ▢ 06 → Ir a P.15
- Ayuda familiar (sin remuneración reglamentada en la empresa o negocio de un familiar) ___________ ▢ 07 → Ir a P.15
- Miembro de una cooperativa ____________________ ▢ 08 → Ir a P.15
- Otra situación________________________________ ▢ 09 → Ir a P.15
- No sabe ____________________________________ ▢ 98 → Ir a P.15
- No contesta _________________________________ ▢ 99 → Ir a P.15

14a. ¿Cuál es la duración de su contrato?

Menor de 6 meses_____________________ ▢ 1→ Ir a P.15
De 6 meses a menos de 1 año ___________ ▢ 2→ Ir a P.15
De 1 año a menos de 2 años_____________ ▢ 3→ Ir a P.15
De 2 años o más _______________________ ▢ 4→ Ir a P.15
Sin duración definida ___________________ ▢ 5→ Ir a P.15
No sabe _____________________________ ▢ 8→ Ir a P.15
No contesta __________________________ ▢ 9→ Ir a P.15

14b. ¿Cuál es la duración de su relación laboral?

Menor de 6 meses ☐ 1
De 6 meses a menos de 1 año ☐ 2
De 1 año a menos de 2 años ☐ 3
De 2 años o más ☐ 4
Sin duración definida ☐ 5
No sabe ☐ 8
No contesta ☐ 9

15. ¿Tiene trabajadores a su cargo?

No ☐ 1
Sí, de 1 a 4 personas ☐ 2
Sí, de 5 a 10 personas ☐ 3
Sí, de 11 a 20 personas ☐ 4
Sí, más de 20 personas ☐ 5
No sabe ☐ 8
No contesta ☐ 9

16. Su trabajo principal actual, ¿es a tiempo completo o a tiempo parcial?

A tiempo completo ☐ 1
A tiempo parcial ☐ 2
No sabe ☐ 8
No contesta ☐ 9

17. ¿Qué tipo de jornada tiene habitualmente?

- Jornada partida ☐ 01
- Jornada continua ☐
  - Mañana ☐ 02
  - Tarde ☐ 03
  - Noche ☐ 04
- Jornada reducida ☐ 05
- Turnos ☐ 06
- Jornada irregular o variable según los días ☐ 07
- Otro tipo ☐ 08
- No sabe ☐ 98
- No contesta ☐ 99

18a. ¿Cuál es la actividad de la empresa u organización en la que trabaja actualmente?

Actividad --

Código de actividad └┴┴┘

Ir a P.19a

18b. ¿Cuál era la actividad de la empresa u organización en la que trabajó en su último empleo?

Actividad --

Código de actividad └┴┴┘

Ir a P.19b

19a. ¿Cuál es la ocupación, profesión u oficio que desempeña en su empleo?

Profesión --
Código de ocupación └┴┴┘ → Ir a Módulo G

19b. ¿Cuál era la ocupación, profesión u oficio que desempeñó en su último empleo?

Profesión --
Código de ocupación └┴┴┘

20. ¿Cuál era la situación profesional en la ocupación que desempeñó en su último empleo?

@Asalariado/a (a sueldo, comisión, jornal...)	▢ 1
@Empresario/a o profesional con asalariados	▢ 2
@Empresario/a sin asalariados o @trabajador/a independiente	▢ 3
Ayuda familiar (sin remuneración reglamentada en la empresa o negocio de un familiar)	▢ 4
Miembro de una cooperativa	▢ 5
Otra situación	▢ 6
No sabe	▢ 8
No contesta	▢ 9

MÓDULO DE ESTADO DE SALUD

G.- ESTADO DE SALUD

Introducción P.21: Entrevistador/a, léale al informante: “**A continuación le voy a preguntar sobre su salud”.**

21. En los últimos doce meses, ¿diría que su estado de salud ha sido muy bueno, bueno, regular, malo, muy malo

Muy bueno ______ ▢ 1
Bueno ______ ▢ 2
Regular ______ ▢ 3
Malo ______ ▢ 4
Muy malo ______ ▢ 5

22. ¿Tiene alguna enfermedad o problema de salud crónicos o de larga duración? (Entendemos por larga duración si el problema de salud o enfermedad ha durado o se espera que dure 6 meses o más)

Sí ▢ 1
No ▢ 2
No sabe ▢ 8
No contesta ▢ 9

23. Durante al menos los últimos 6 meses, ¿en qué medida se ha visto limitado/a debido a un problema de salud para realizar las actividades que la gente habitualmente hace?

Gravemente limitado/a ______ ▢ 1
Limitado/a, pero no gravemente ______ ▢ 2
Nada limitado/a ______ ▢ 3
No sabe ______ ▢ 8
No contesta ______ ▢ 9

Si P.23 = 3, 8 ó 9 → Ir a la introducción de la P24a

24. ¿Qué tipo de problema es la causa de su dificultad para realizar las actividades que la gente habitualmente hace?

Físico ____________ ▢ 1
Mental ____________ ▢ 2
Ambos ____________ ▢ 3
No sabe __________ ▢ 8
No contesta _______ ▢ 9

Introducción P.24A: Entrevistador/a, léale al informante: “**A continuación le voy a preguntar sobre su salud bucondental”.**

24A ¿Cómo describiría su estado de salud bucodental: muy bueno, bueno, regular, malo, muy malo?

Muy bueno ______________________________ ▢ 1
Bueno _________________________________ ▢ 2
Regular ________________________________ ▢ 3
Malo __________________________________ ▢ 4
Muy malo ______________________________ ▢ 5
No sabe ________________________________ ▢ 8
No contesta _____________________________ ▢ 9

25. A continuación le voy a leer una lista con una serie de enfermedades o problemas de salud. ¿Padece o ha padecido alguna vez alguna de ellas?

	25.a ¿Alguna vez ha padecido ...?		25.b ¿La ha padecido en los últimos 12 meses?		25.c ¿Le ha dicho un médico que la padece?	
Tensión alta	SI (1)<br>NO (2)	NS(8)<br>NC (9)	SI (1)<br>NO (2)	NS(8)<br>NC (9)	SI (1)<br>NO (2)	NS(8)<br>NC (9)
Infarto de miocardio	SI (1)<br>NO (2)	NS(8)<br>NC (9)	SI (1)<br>NO (2)	NS(8)<br>NC (9)	SI (1)<br>NO (2)	NS(8)<br>NC (9)
Angina de pecho, enfermedad coronaria	SI (1)<br>NO (2)	NS(8)<br>NC (9)	SI (1)<br>NO (2)	NS(8)<br>NC (9)	SI (1)<br>NO (2)	NS(8)<br>NC (9)
Otras enfermedades del corazón	SI (1)<br>NO (2)	NS(8)<br>NC (9)	SI (1)<br>NO (2)	NS(8)<br>NC (9)	SI (1)<br>NO (6)	NS(8)<br>NC (9)

Lista de Enfermedades que deben aparecer en la pregunta:

1. Tensión alta
2. Infarto de miocardio
3. Angina de pecho, enfermedad coronaria
4. Otras enfermedades del corazón
5. Varices en las piernas
6. Artrosis (excluyendo artritis)
7. Dolor de espalda crónico (cervical)
8. Dolor de espalda crónico (lumbar)
9. Alergia crónica, como rinitis, conjuntivitis o dermatitis alérgica, alergia alimentaria o de otro tipo (asma alérgica excluida)
10. Asma (incluida asma alérgica)
11. Bronquitis crónica, enfisema, enfermedad pulmonar obstructiva crónica (EPOC)
12. Diabetes
13. Úlcera de estómago o duodeno
14. Incontinencia urinaria o problemas de control de la orina
15. Colesterol alto
16. Cataratas
17. Problemas crónicos de piel
18. Estreñimiento crónico
19. Cirrosis, disfunción hepática
20. Depresión
21. Ansiedad crónica
22. Otros problemas mentales
23. Ictus (embolia, infarto cerebral, hemorragia cerebral)
24. Migraña o dolor de cabeza frecuente
25. Hemorroides
26. Tumores malignos
27. Osteoporosis
28. Problemas de tiroides
29. Problemas de riñón

Filtro: (para infarto)
Si P25.a_2 = 1 y Si P25.b_2 = 2,8,9 en la enfermedad 2. (infarto de miocardio) preguntar P25d_2 después de preguntar P25c_2

En otro caso no preguntar

P25.d._2 ¿Ha sufrido, en los últimos 12 meses ,secuelas del infarto de miocardio que padeció?

Sí ▢ 1 No ▢ 2 No sabe ▢ 8 No contesta ▢ 9

Filtro: (para ictus)
Si P25.a_23 = 1 y Si P25.b_23 = 2, 8, 9 en la enfermedad 23. (ioctus) preguntar P25d_23 después de preguntar P25c_23

En otro caso no preguntar

P25.d._23 ¿Ha sufrido,en los últimos 12 meses ,secuelas del ictus que padeció?

Sí ▢ 1 No ▢ 2 No sabe ▢ 8 No contesta ▢ 9

30. Problemas de próstata (solo hombres)
31. Problemas del periodo menopáusico (solo mujeres)
32. Lesiones o defectos permanentes causados por un accidente

H.- ACCIDENTALIDAD

26. Durante los últimos 12 meses, ¿ha tenido alguno de los siguientes tipos de accidente en el que haya resultado herido/a o lesionado/a?

Nota P.26: Se incluyen los casos de intoxicación, picaduras de insectos y las heridas provocadas por animales. Se excluyen los actos intencionados por parte de otra persona.

	Sí	No	No sabe	No contesta
**A. Accidente de tráfico**	☐ (1)	☐ (2)	☐ (8)	☐ (9)
**B. Accidente en casa**	☐ (1)	☐ (2)	☐ (8)	☐ (9)
**C. Accidente durante su tiempo libre**	☐ (1)	☐ (2)	☐ (8)	☐ (9)

Si P26.A=1 o P26.B=1 o P26.C=1 → Ir a P.27
En otro caso ir a Filtro P30.

27. ¿Recibió atención médica como consecuencia de este/os accidente/s?

Nota P.27: No se admite respuesta múltiple. En caso de que el informante haya tenido más de un accidente, la respuesta debe reflejar la atención médica recibida por el accidente más grave.

Ingresó en un hospital________________________ ☐ 1
Acudió a un centro de urgencias________________ ☐ 2
Consultó a un médico o enfermero/a____________ ☐ 3
No hizo ninguna consulta ni intervención_________ ☐ 4
No sabe___________________________________ ☐ 8
No contesta________________________________ ☐ 9

Filtro P30.
Si P.11 (Cuestionario de Hogar) = 1 para el adulto seleccionado → Ir a P.30
Si P.11 (Cuestionario de Hogar) <>1 para el adulto seleccionado → Ir a Introducción P.32

J.- AUSENCIA DEL TRABAJO POR PROBLEMAS DE SALUD

30. En los últimos 12 meses, ¿ha faltado al trabajo por problemas de salud? Tenga en cuenta todas las clases de enfermedad, problemas de salud o lesiones que usted padeció y por los que tuvo que faltar a su trabajo.

Sí____________________ ☐ 1
No____________________ ☐ 2
No sabe_______________ ☐ 8
No contesta____________ ☐ 9

Si P.30 <>1 → Ir a Introducción P.32

31. ¿Y cuántos días faltó en total?

|__|__|__| días

No sabe________________ ☐ 998
No contesta_____________ ☐ 999

K.- LIMITACIONES FÍSICAS Y SENSORIALES

Introducción P.32: Entrevistador/a, léale al informante: **"A continuación le voy a preguntar sobre situaciones en las que podría encontrarse en su vida diaria. Por favor, no tenga en cuenta los problemas temporales."**

32. ¿Utiliza gafas o lentillas?

Sí______________________________________ ☐ 1
No______________________________________ ☐ 2
Soy ciego/a o no puedo ver en absoluto________ ☐ 3
No sabe__________________________________ ☐ 8
No contesta_______________________________ ☐ 9

Si P.32=3,8,9 → Ir a P.34
Si P.32=1 mostrar P33.a
Si P.32=2 mostrar P33.b

33a. ¿Tiene dificultad para ver utilizando sus gafas o lentillas?

33b. ¿Tiene dificultad para ver?

No, ninguna dificultad ☐ 1
Sí, alguna dificultad ☐ 2
Sí, mucha dificultad ☐ 3
No puedo ver en absoluto ☐ 4
No sabe ☐ 8
No contesta ☐ 9

34. ¿Utiliza audífono?

Sí ☐ 1
No ☐ 2
Soy sordo/a profundo/a ☐ 3
No sabe ☐ 8
No contesta ☐ 9

Si P.34=3,8,9 → Ir a P.37
Si P.34=1 mostrar P35.a
Si P.34=2 mostrar P35.b

35a. ¿Tiene dificultad para oír lo que se dice en una conversación con otra persona en un sitio tranquilo utilizando su audífono?

35b. ¿Tiene dificultad para oír lo que se dice en una conversación con otra persona en un sitio tranquilo?

No, ninguna dificultad ☐ 1
Sí, alguna dificultad ☐ 2
Sí, mucha dificultad ☐ 3
No puedo hacerlo en absoluto ☐ 4
No sabe ☐ 8
No contesta ☐ 9

Si P.35 = 4, 8, 9 → Ir a P.37
Si P.34=1 mostrar P36.a
Si P.34=2 mostrar P36.b

36a. ¿Tiene dificultad para oír lo que se dice en una conversación con otra persona en un sitio más ruidoso aunque utilice su audífono?

36b. ¿Tiene dificultad para oír lo que se dice en una conversación con otra persona en un sitio más ruidoso?

No, ninguna dificultad ☐ 1
Sí, alguna dificultad ☐ 2
Sí, mucha dificultad ☐ 3
No puedo hacerlo en absoluto ☐ 4
No sabe ☐ 8
No contesta ☐ 9

37. ¿Tiene dificultad para caminar 500 metros sobre un terreno llano sin ningún tipo de ayuda para andar?

No, ninguna dificultad ☐ 1
Sí, alguna dificultad ☐ 2
Sí, mucha dificultad ☐ 3
No puedo hacerlo en absoluto ☐ 4
No sabe ☐ 8
No contesta ☐ 9

38. ¿Tiene dificultad para subir o bajar 12 escalones?

No, ninguna dificultad ☐ 1
Sí, alguna dificultad ☐ 2
Sí, mucha dificultad ☐ 3
No puedo hacerlo en absoluto ☐ 4
No sabe ☐ 8
No contesta ☐ 9

38.a ¿Tiene dificultad para recordar o para concentrarse?

No, ninguna dificultad ☐ 1
Sí, alguna dificultad ☐ 2
Sí, mucha dificultad ☐ 3
No puedo hacerlo en absoluto ☐ 4
No sabe ☐ 8
No contesta ☐ 9

Si la persona seleccionada tiene menos de 55 años → Ir a Introducción P.45

38.b ¿Tiene dificultad para morder o masticar alimentos duros?

No, ninguna dificultad ________ ▢ 1
Sí, alguna dificultad ________ ▢ 2
Sí, mucha dificultad ________ ▢ 3
No puedo hacerlo en absoluto ________ ▢ 4
No sabe ________ ▢ 8
No contesta ________ ▢ 9

L.- LIMITACIONES PARA LA REALIZACIÓN DE LAS ACTIVIDADES DE LA VIDA COTIDIANA

Introducción P.39: Entrevistador/a, léale al informante: **"Piense ahora en sus actividades básicas diarias. No tenga en cuenta los problemas temporales."**

39. De las actividades que le voy a leer, ¿habitualmente tiene dificultad para hacerlas sin ayuda?

**Actividades**	**No, ninguna dificultad**	**Sí, alguna dificultad**	**Sí, mucha dificultad**	**No puedo hacerlo por mí mismo/a**		**No contesta**
**A. Alimentarse**	▢ (1)	▢ (2)	▢ (3)	▢ (4)	▢ (8)	▢ (9)
**B. Sentarse, levantarse de una silla o de una cama, acostarse**	▢ (1)	▢ (2)	▢ (3)	▢ (4)	▢ (8)	▢ (9)
**C Vestirse y desvestirse**	▢ (1)	▢ (2)	▢ (3)	▢ (4)	▢ (8)	▢ (9)
**D. Ir al servicio**	▢ (1)	▢ (2)	▢ (3)	▢ (4)	▢ (8)	▢ (9)
**E. Ducharse o bañarse**	▢ (1)	▢ (2)	▢ (3)	▢ (4)	▢ (8)	▢ (9)

Si P39.A=2,3,4 o P39.B=2,3,4 o P39.C=2,3,4 o P39.D=2,3,4 o P39.E=2,3,4 → Ir a Introducción P.40

En otro caso → ir a Introducción P.42

Introducción P.40: Entrevistador/a, léale al informante: **"Considerando esta actividad o actividades en las que usted encuentra dificultades para hacerlas".**

40. ¿Dispone habitualmente de ayuda para realizarlas?

Sí, al menos para una actividad______ ▢ 1
No______________________________ ▢ 2
No sabe__________________________ ▢ 8
No contesta______________________ ▢ 9

Si P.40 = 8,9 → Ir a Introducción P.42
Si P.40 =1 mostrar P41.a
Si P.40 =2 mostrar P41.b

P41a. ¿Necesitaría más ayuda de la que dispone?
P41b. ¿Considera que necesita ayuda?

Sí, al menos para una actividad______ ▢ 1
No______________________________ ▢ 2
No sabe__________________________ ▢ 8
No contesta______________________ ▢ 9

Introducción P.42: Entrevistador/a, léale al informante: **"A continuación, le voy a leer una serie de actividades relacionadas con el hogar. De nuevo, no incluya problemas temporales".**

P42. ¿Habitualmente tiene dificultad para hacer por sí mismo/a y sin ayuda alguna de estas actividades?

**Actividades**	**No, ninguna dificultad**	**Sí, alguna dificultad**	**Sí, mucha dificultad**	**No puedo hacerlo en absoluto**	**No aplicable (nunca lo ha intentado o necesitado hacerlo)**	**No Sabe**	**No Contest a**
**A. Preparar las comidas**	▢ (1)	▢ (2)	▢ (3)	▢ (4)	▢ (5)	▢ (8)	▢ (9)
**B. Utilizar el teléfono (buscar el número, marcar…)**	▢ (1)	▢ (2)	▢ (3)	▢ (4)	▢ (5)	▢ (8)	▢ (9)

**C. Realizar compras (comprar la comida, la ropa…)**	▢ (1)	▢ (2)	▢ (3)	▢ (4)	▢ (5)	▢ (8)	▢ (9)
**D. Tomar sus medicamentos, incluyendo acordarse de la cantidad y el momento en que los debe tomar**	▢ (1)	▢ (2)	▢ (3)	▢ (4)	▢ (5)	▢ (8)	▢ (9)
**E. Realizar tareas domésticas ligeras como hacer la colada, hacer la cama, limpiar la casa…**	▢ (1)	▢ (2)	▢ (3)	▢ (4)	▢ (5)	▢ (8)	▢ (9)
**F. Realizar ocasionalmente tareas domésticas que requieren un gran esfuerzo como mover muebles, limpiar las ventanas, transportar la compra…**	▢ (1)	▢ (2)	▢ (3)	▢ (4)	▢ (5)	▢ (8)	▢ (9)
**G. Administrar su propio dinero (pagar recibos, tratar con el banco, firmar cheques…)**	▢ (1)	▢ (2)	▢ (3)	▢ (4)	▢ (5)	▢ (8)	▢ (9)

Si P42.A=2,3,4 o P42.B=2,3,4 o P42.C=2,3,4 o P42.D=2,3,4 o P42.E=2,3,4 o P42.F=2,3,4 o P42.G=2,3,4 → Ir a Introducción P.43

En otro caso → Ir a Introducción P.45

Introducción P.43: Entrevistador/a, léale al informante: **"Considerando esta actividad o actividades en las que usted encuentra dificultades para hacerlas."**

43. ¿Dispone habitualmente de ayuda para realizarlas?

Sí, al menos para una actividad______ ▢ 1
No______________________________ ▢ 2
No sabe__________________________ ▢ 8
No contesta______________________ ▢ 9

Si P.43 = 8,9 → Ir a Introducción P.45
Si P.43 = 1 mostrar P44.a
Si P.43 = 2 mostrar P44.b

P44a. ¿Necesitaría más ayuda de la que dispone?

P44b. ¿Considera que necesita ayuda?

Sí, al menos para una actividad______ ▢ 1
No________________________________ ▢ 2
No sabe____________________________ ▢ 8
No contesta________________________ ▢ 9

Introducción P.45: Entrevistador/a, léale al informante: **"Las siguientes preguntas se refieren al dolor físico que ha podido tener en las últimas 4 semanas".**

P45. Durante las 4 últimas semanas, ¿qué grado de dolor ha padecido?

Ninguno___________________ ▢ 1
Muy leve__________________ ▢ 2
Leve______________________ ▢ 3
Moderado__________________ ▢ 4
Severo____________________ ▢ 5
Extremo___________________ ▢ 6
No sabe___________________ ▢ 8
No contesta_______________ ▢ 9

P46. Durante las 4 últimas semanas, ¿hasta qué punto el dolor afectó a sus actividades cotidianas?

Nada______________________ ▢ 1
Un poco___________________ ▢ 2
Moderadamente_____________ ▢ 3
Bastante__________________ ▢ 4
Mucho_____________________ ▢ 5
No sabe___________________ ▢ 8
No contesta_______________ ▢ 9

M.- SALUD MENTAL

47. Durante las últimas 2 semanas, ¿con qué frecuencia ha tenido alguno de los siguientes problemas?

	Nunca	Varios días	Más de la mitad de los días	Casi todos los días	No sabe	No contesta
**A. Poco interés o alegría por hacer cosas**	☐ (1)	☐ (2)	☐ (3)	☐ (4)	☐ (8)	☐ (9)
**B. Sensación de estar decaído/a, deprimido/a o desesperanzado/a**	☐ (1)	☐ (2)	☐ (3)	☐ (4)	☐ (8)	☐ (9)
**C. Problemas para quedarse dormido/a, para seguir durmiendo o dormir demasiado**	☐ (1)	☐ (2)	☐ (3)	☐ (4)	☐ (8)	☐ (9)
**D. Sensación de cansancio o de tener poca energía**	☐ (1)	☐ (2)	☐ (3)	☐ (4)	☐ (8)	☐ (9)
**E. Poco apetito o comer demasiado**	☐ (1)	☐ (2)	☐ (3)	☐ (4)	☐ (8)	☐ (9)
**F. Sentirse mal consigo mismo/a, sentirse que es un fracasado/a o que ha decepcionado a su familia o a sí mismo/a**	☐ (1)	☐ (2)	☐ (3)	☐ (4)	☐ (8)	☐ (9)
**G. Problemas para concentrarse en algo, como leer el periódico o ver la televisión**	☐ (1)	☐ (2)	☐ (3)	☐ (4)	☐ (8)	☐ (9)
**H. Moverse o hablar tan despacio que los demás pueden haberlo notado. O lo contrario: estar tan inquieto/a o agitado/a que se ha estado moviendo de un lado a otro más de lo habitual**	☐ (1)	☐ (2)	☐ (3)	☐ (4)	☐ (8)	☐ (9)

MÓDULO DE ASISTENCIA SANITARIA

N.- CONSULTAS MÉDICAS Y OTROS SERVICIOS AMBULATORIOS

Introducción P.48: Entrevistador/a, léale al informante: **"El siguiente grupo de preguntas hace referencia a las consultas con su médico general o médico de familia. Por favor, incluya tanto las visitas a la consulta de su médico, como las visitas domiciliarias y las consultas telefónicas."**

48. ¿Cuándo fue la última vez que consultó al médico general o médico de familia para usted mismo/a?

En las últimas 4 semanas____________ ▢ 1
Entre 4 semanas y 12 meses_________ ▢ 2
Hace 12 meses o más_______________ ▢ 3
Nunca_____________________________ ▢ 4

Si P.48= 2 → Imputar P.49=0 e ir a Introducción P.50
Si P.48= 3,4 → Ir a Introducción P.50

49. Durante las últimas 4 semanas contando desde ayer, es decir, desde el _______(fecha del día anterior a la entrevista menos 28 días), ¿cuántas veces ha consultado con su médico general o médico de familia para usted mismo/a?

|__|__| Nº de veces NS ▢ 98 NC ▢99

Introducción P.50: Entrevistador/a, léale al informante: **"Las siguientes preguntas hacen referencia a consultas a especialistas. Se incluyen sólo las visitas a médicos en consultas externas o ambulatorias, pero no las visitas realizadas en el hospital como paciente ingresado o atendido en un hospital de día. No deben incluirse las visitas al dentista, aunque sí al cirujano maxilofacial."**

50. ¿Cuándo fue la última vez que consultó a un especialista para usted mismo/a?

En las últimas 4 semanas______________ ▢ 1
Entre 4 semanas y 12 meses____________ ▢ 2
Hace 12 meses o más_________________ ▢ 3
Nunca______________________________ ▢ 4
No sabe____________________________ ▢ 8
No contesta_________________________ ▢ 9

Si P.50= 2 → Imputar P.51=0 e ir a Filtro P.53

Si P.50= 3, 4, 8, 9 → Ir a Filtro P.53

51. Durante las últimas 4 semanas contando desde ayer, es decir, desde el _______ (fecha del día anterior a la entrevista menos 28 días), ¿cuántas veces consultó a un especialista para usted mismo/a?

|__|__| Nº de veces NS ▢ 98 NC ▢99

FILTRO P.52:
Si P.48=1 y P.50=1 → Ir a P.52
En otro caso → Ir a Filtro P.53

52. El último médico que consultó, ¿era médico de familia/general o era especialista?

Médico de familia o médico general__________ ▢ 1
Especialista_______________________________ ▢ 2
No sabe__________________________________ ▢ 8
No contesta_______________________________ ▢ 9

Filtro P.53
Si P.48=1 o P.50=1 → Ir a P.53
En otro caso → Ir a P.58

53. ¿Dónde tuvo lugar la última consulta realizada durante las últimas cuatro semanas?

Centro de Salud/Consultorio ________________ ▢ 01
Ambulatorio/Centro de especialidades ________ ▢ 02
Consulta externa de un hospital ____________ ▢ 03
Servicio de Urgencias no hospitalario ________ ▢ 04
Servicio de Urgencias de un hospital_________ ▢ 05
Consulta de médico de una sociedad ________ ▢ 06
Consulta de médico particular ______________ ▢ 07
Empresa o lugar de trabajo ________________ ▢ 08
Domicilio del entrevistado _________________ ▢ 09
Consulta telefónica ______________________ ▢ 10
Otro lugar_______________________________ ▢ 11
No sabe _________________________________ ▢ 98
No contesta ______________________________ ▢ 99

Si P.53 = 04 ó 05 → Ir a P.57

54. ¿Cuál fue el motivo principal de esta última consulta?

Diagnóstico de una enfermedad o problema de salud ____ ▢ 1
Un accidente o agresión ____________________________ ▢ 2
Revisión____ ▢ 3
Sólo dispensación de recetas ________________________ ▢ 4
Parte de baja, confirmación o alta ____________________ ▢ 5
Otros motivos_____________________________________ ▢ 6
No sabe ____ ▢ 8
No contesta ______________________________________ ▢ 9

Filtro P.57
Si P.53 = 01 ó 02 → Ir a P.57. Disponibles todas las categorías de respuesta.
Si P.53 = 06 ó 07 → Disponibles en P.57 solo respuestas 2, 3, 8 y 9
Si P.53 = 08 → Marcar en P.57 = 4 e Ir a P.58

57. El médico al que acudió en esta última consulta realizada era de:

Sanidad Pública (Seguridad Social)__________________ ▢ 1
Sociedad médica __________________________________ ▢ 2
Consulta privada __________________________________ ▢ 3
Otros (médico de empresa, etc.) ____________________ ▢ 4
No sabe ____ ▢ 8
No contesta ______________________________________ ▢ 9

58. En los últimos 12 meses, es decir, desde _______ (fecha de entrevista menos un año), ¿ha visitado para usted mismo/a un...?

	Sí	No	No sabe	No contesta
**Fisioterapeuta**	▢ (1)	▢ (2)	▢ (8)	▢ (9)
**Psicólogo, psicoterapeuta o psiquiatra**	▢ (1)	▢ (2)	▢ (8)	▢ (9)
**Enfermero/a o matrona**	▢ (1)	▢ (2)	▢ (8)	▢ (9)

59. Durante los últimos 12 meses, es decir, desde ______ (fecha de entrevista menos un año), ¿se ha realizado algún análisis?

Sí___________________ ▢ 1
No __________________ ▢ 2
No sabe______________ ▢ 8
No contesta___________ ▢ 9

Nota P.59: **Entrevistador/a,** se incluye análisis de sangre, orina, heces, etc.

60. Durante los últimos 12 meses, es decir, desde ______ (fecha de entrevista menos un año), ¿se ha realizado alguna de las siguientes pruebas?

	Sí	No	No sabe	No contesta
**Radiografía**	▢ (1)	▢ (2)	▢ (8)	▢ (9)
**TAC o escáner**	▢ (1)	▢ (2)	▢ (8)	▢ (9)
**Ecografía**	▢ (1)	▢ (2)	▢ (8)	▢ (9)
**Resonancia magnética**	▢ (1)	▢ (2)	▢ (8)	▢ (9)

60.a- Durante los últimos 12 meses, es decir, desde ______ (fecha de entrevista menos un año), ¿ha visitado para usted @mismo/a a un…?

	Sí	No	No sabe	No contesta
**Homeópata**	▢ (1)	▢ (2)	▢ (8)	▢ (9)
**Acupuntor**	▢ (1)	▢ (2)	▢ (8)	▢ (9)
**Naturista**	▢ (1)	▢ (2)	▢ (8)	▢ (9)
**Otro profesional de medina alternativa (osteópata, quiropráctico…)**	▢ (1)	▢ (2)	▢ (8)	▢ (9)

61. Durante los últimos 12 meses, es decir, desde ______ (fecha de entrevista menos un año), ¿ha utilizado personalmente alguno de los siguientes servicios asistenciales?

	Sí	No	No sabe	No contesta
**Atención domiciliaria por una enfermera o matrona**	▢ (1)	▢ (2)	▢ (8)	▢ (9)
**Ayuda domiciliaria para las tareas domésticas o para las personas mayores**	▢ (1)	▢ (2)	▢ (8)	▢ (9)
**Comidas a domicilio para personas mayores**	▢ (1)	▢ (2)	▢ (8)	▢ (9)
**Servicios especiales de transporte a domicilio para acudir a un servicio médico, a un hospital de día, actividades recreativas…**	▢ (1)	▢ (2)	▢ (8)	▢ (9)
**Otros servicios de asistencia domiciliaria**	▢ (1)	▢ (2)	▢ (8)	▢ (9)

La tercera opción: *“Comidas a domicilio para personas mayores*” solo aparece si la persona adulta seleccionada es mayor de 65 años.

Introducción P.62: Entrevistador/a, léale al informante: **"A continuación voy a hacerle unas preguntas sobre su salud dental."**

62. ¿Cuándo fue la última vez que visitó al dentista, estomatólogo o higienista dental para usted mismo/a (es decir, no sólo como acompañante de un niño, de su pareja, etc.)?

Hace 3 meses o menos___________________ ☐ 1 62.b ¿Cuántas veces en los últimos 3 meses? └─┴─┘ NS ☐ 98 NC ☐ 99
Hace más de 3 meses y menos de 6_________ ☐ 2
Hace 6 meses o más pero menos de 12______ ☐ 3
Hace 12 meses o más____________________ ☐ 4
Nunca_________________________________ ☐ 5

Si P.62 = 1 se activa P.62b
Si P.62= 5 → Ir a P.65

63. La última vez que acudió, ¿cuál o cuáles de los siguientes tipos de asistencia recibió?

	Sí	No	No sabe	No contesta
**Revisión o chequeo**	☐ (1)	☐ (2)	☐ (8)	☐ (9)
**Limpieza de boca**	☐ (1)	☐ (2)	☐ (8)	☐ (9)
**Empastes (obturaciones), endodoncias**	☐ (1)	☐ (2)	☐ (8)	☐ (9)
**Extracción de algún diente/muela**	☐ (1)	☐ (2)	☐ (8)	☐ (9)
**Fundas, puentes u otro tipo de prótesis**	☐ (1)	☐ (2)	☐ (8)	☐ (9)
**Tratamiento de las enfermedades de las encías**	☐ (1)	☐ (2)	☐ (8)	☐ (9)
**Ortodoncia**	☐ (1)	☐ (2)	☐ (8)	☐ (9)
**Aplicación de flúor**	☐ (1)	☐ (2)	☐ (8)	☐ (9)
**Implantes**	☐ (1)	☐ (2)	☐ (8)	☐ (9)
**Otro tipo de asistencia**	☐ (1)	☐ (2)	☐ (8)	☐ (9)

64. El dentista, estomatólogo o higienista dental al que consultó esta última vez era de:

Sanidad Pública (Seguridad Social, ayuntamiento, consulta privada financiada por el gobierno autónomo) ________________ ▢ 1
Sociedad médica ________________ ▢ 2
Consulta privada ________________ ▢ 3
Otros ________________ ▢ 4
No sabe ________________ ▢ 8
No contesta ________________ ▢ 9

65. ¿Cuál es el estado de sus dientes y muelas?

	Sí	No	No sabe	No contesta
**Tiene caries**	▢ (1)	▢ (2)	▢ (8)	▢ (9)
**Le han extraído dientes/muelas**	▢ (1)	▢ (2)	▢ (8)	▢ (9)
**Tiene dientes/muelas empastados (obturados)**	▢ (1)	▢ (2)	▢ (8)	▢ (9)
**Le sangran las encías al cepillarse o espontáneamente**	▢ (1)	▢ (2)	▢ (8)	▢ (9)
**Se le mueven los dientes/muelas**	▢ (1)	▢ (2)	▢ (8)	▢ (9)
**Lleva fundas (coronas), puentes, otro tipo de prótesis o dentadura postiza**	▢ (1)	▢ (2)	▢ (8)	▢ (9)
**Le faltan dientes/muelas que no han sido sustituidos por prótesis**	▢ (1)	▢ (2)	▢ (8)	▢ (9)
**Tiene o conserva todos sus dientes/muelas naturales**	▢ (1)	▢ (2)	▢ (8)	▢ (9)

O.- HOSPITALIZACIONES, URGENCIAS Y SEGURO SANITARIO

Introducción P.66: Entrevistador/a, léale al informante: **"El siguiente grupo de preguntas hace referencia al tiempo pasado en un hospital. Se incluyen todos los tipos de hospital".**

Si la persona seleccionada es: (hombre) o (mujer de 52 años o más), mostrar P66.a
Si la persona seleccionada es mujer menor de 52 años, mostrar P66.b

66a. Durante los últimos doce meses, ¿ha tenido que ingresar como paciente al menos durante una noche?

66b. Durante los últimos doce meses, ¿ha tenido que ingresar como paciente al menos durante una noche excluyendo partos o cesáreas?

Sí___________________ ▢ 1
No __________________ ▢ 2

Si P.66 = 1 → Ir a P.67

Si P.66 = 2 y es una mujer menor de 52 años → Ir a P.69

Resto de los casos → Ir a P.75

Si la persona seleccionada es: (hombre) o (mujer de 52 años o más), mostrar P67.a
Si la persona seleccionada es mujer menor de 52 años, mostrar P67.b

67a. ¿Cuántas veces ha estado hospitalizado/a en estos últimos doce meses?

67b. ¿Cuántas veces ha estado hospitalizada en estos últimos doce meses? **Nuevamente, no considere los partos o cesáreas.**

Nº de veces |_|_| NS ▢ 98 NC ▢99

68. Considerando estos ingresos, ¿cuántas noches en total ha pasado ingresado/a en un hospital en estos últimos 12 meses?

|_|_|_| Noches NS ▢ 998 NC ▢ 999

Si la persona seleccionada es mujer menor de 52 años ---> Ir a P.69
Si la persona seleccionada es (hombre) o (mujer de 52 años o más) ---> Ir a P.71

69. Durante los últimos doce meses, ¿ha tenido que ingresar en un hospital por parto o cesárea?

Sí___________________ ▢ 1
No __________________ ▢ 2
No sabe______________ ▢ 8
No contesta___________ ▢ 9

Nota P.69: No deben incluirse las visitas a urgencias ni a hospitales de día.

Si P.69= 2, 8, 9 y P.66 = 2 → Ir a P.75

71. En relación al último ingreso hospitalario, ¿cuál fue el motivo de su ingreso en el hospital?

Intervención quirúrgica________________ ▢ 1
Estudio médico para diagnóstico________________ ▢ 2
Tratamiento médico sin intervención quirúrgica________________ ▢ 3
Parto (incluye cesárea)________________ ▢ 4
Otros motivos________________ ▢ 5
No sabe ________________ ▢ 8
No contesta ________________ ▢ 9

74. ¿A cargo de quién corrieron los gastos de su hospitalización?

Sanidad Pública (Seguridad Social)________________ ▢ 1
Mutualidad obligatoria (MUFACE, ISFAS, etc.) ________________ ▢ 2
Sociedad médica privada________________ ▢ 3
A su propio cargo o de su hogar ________________ ▢ 4
A cargo de otras personas, organismos o instituciones________________ ▢ 5
No sabe ________________ ▢ 8
No contesta ________________ ▢ 9

<u>Hospital de día</u>

75. Durante los últimos doce meses, desde el _______ (fecha de entrevista menos un año), ¿ha sido usted atendido/a en un Hospital de día para una intervención, tratamiento o hacerse alguna prueba, es decir, permaneciendo durante todo o parte del día pero sin tener que pasar allí la noche?
(Incluya ingresos en cama o sillón cama, no incluya estancias en urgencias ni en observación)

Sí________________ ▢ 1
No ________________ ▢ 2
No sabe________________ ▢ 8
No contesta________________ ▢ 9

Si P.75 <>1 → Ir a P.78

76. ¿Cuántos días ha tenido que acudir a un hospital de día, sin tener que pasar la noche?

|__|__|__| Días NS ▢ 998 NC ▢999

Servicios de urgencia

Introducción P.78: Entrevistador/a, léale al informante: "**Para terminar este apartado, voy a preguntarle ahora por su posible utilización de los servicios de urgencias".**

78. En estos últimos doce meses, ¿ha tenido que utilizar algún servicio de urgencias por algún problema o enfermedad?

Sí________________ ▢ 1
No ________________ ▢ 2

Si P.78= 2 → Ir a P.84

79. Y en total, ¿cuántas veces tuvo que utilizar un servicio de urgencias en estos doce meses?

Nº de veces |__|__|__| NS ▢ 998 NC ▢ 999

80. ¿Dónde fue atendido la última vez?

Nota P.80: se admite respuesta múltiple.

En el lugar donde se encontraba (domicilio, lugar de trabajo, etc.)____▢
En una unidad móvil____▢
En un centro o servicio de urgencias____________________________▢
No sabe____▢
No contesta____▢

81. La última vez que utilizó un servicio de urgencias, ¿cuánto tiempo pasó desde que empezó a notarse enfermo/a o sintió que tenía algún problema de salud hasta que pidió asistencia?

Días ____________ ⌴⌴
Horas ___________ ⌴⌴
Minutos__________ ⌴⌴
No sabe ________________ ▢ 98
No contesta ____________ ▢ 99

82. Y también esta última vez que utilizó un servicio de urgencias, ¿cuánto tiempo pasó desde que pidió asistencia hasta que fue @atendido/a?

Horas ___________ ⌴⌴
Minutos__________ ⌴⌴
No sabe ________________ ▢ 98
No contesta ____________ ▢ 99

83. Y también esta última vez que utilizó un servicio de urgencias en estos doce últimos meses, ¿qué tipo de servicio utilizó?

Hospital de la Sanidad Pública (Seguridad Social) ____________________ ▢ 1
Centro o servicio de urgencias no hospitalario de la Sanidad Pública (Seguridad Social). Por ejemplo, centro de salud, ambulatorio etc. _______ ▢ 2
Sanatorio, hospital o clínica privada__________________________________ ▢ 3
Servicio privado de urgencias ______________________________________ ▢ 4
Casa de socorro o servicio de urgencias del ayuntamiento _____________ ▢ 5
Otro tipo de servicio ____ ▢ 6
No sabe ____ ▢ 8
No contesta ____ ▢ 9

84. ¿Podría decirme ahora, de cuál o cuáles de las siguientes modalidades de Seguro Sanitario (público y/o privado) es usted titular o beneficiario/a?

- Sanidad pública (Seguridad Social) ☐
- Mutualidades del Estado (MUFACE, ISFAS, MUGEJU) acogidas a la Seguridad Social ☐
- Mutualidades del Estado (MUFACE, ISFAS, MUGEJU) acogidas a un seguro privado ☐
- Seguro médico privado, concertado individualmente (sociedades médicas, Colegios Profesionales, etc.) ☐
- Seguro médico concertado por la empresa ☐
- No tengo seguro médico ☐
- Otras situaciones ☐
- No sabe ☐
- No contesta ☐

P.- CONSUMO DE MEDICAMENTOS

Introducción P.85: Entrevistador/a, léale al informante: **"A continuación, le voy a preguntar sobre el consumo de medicamentos en las últimas 2 semanas."**

85. Durante las dos últimas semanas, ¿ha consumido algún medicamento que le fuera recetado por un médico?

- Sí ☐ 1
- No ☐ 2

86. Durante las últimas dos semanas, ¿ha consumido algún medicamento, incluyendo medicamentos a base de plantas o vitaminas que no le fueran recetados por un médico?

Sí__________________ ☐ 1
No _________________ ☐ 2
No sabe_____________ ☐ 8
No contesta__________ ☐ 9

Si P.85=1 o P.86=1 → Ir a P.87
En otro caso → Ir a P.88

87. A continuación voy a leerle una lista de tipos de medicamentos, por favor dígame ¿cuál o cuáles de ellos ha consumido en las últimas dos semanas y cuáles le fueron recetados por el médico?

	Consumido				Recetado			
	Sí	No	NS	NC	Sí	No	NS	NC
1. Medicinas para el catarro, gripe, garganta, bronquios	☐ (1)	☐ (2)	☐ (8)	☐ (9)	☐ (1)	☐ (2)	☐ (8)	☐ (9)
2. Medicinas para el dolor	☐ (1)	☐ (2)	☐ (8)	☐ (9)	☐ (1)	☐ (2)	☐ (8)	☐ (9)
3. Medicinas para bajar la fiebre	☐ (1)	☐ (2)	☐ (8)	☐ (9)	☐ (1)	☐ (2)	☐ (8)	☐ (9)
4. Reconstituyentes como vitaminas, minerales, tónicos	☐ (1)	☐ (2)	☐ (8)	☐ (9)	☐ (1)	☐ (2)	☐ (8)	☐ (9)
5. Laxantes	☐ (1)	☐ (2)	☐ (8)	☐ (9)	☐ (1)	☐ (2)	☐ (8)	☐ (9)
6. Antibióticos	☐ (1)	☐ (2)	☐ (8)	☐ (9)	☐ (1)	☐ (2)	☐ (8)	☐ (9)
7. Tranquilizantes, relajantes, pastillas para dormir	☐ (1)	☐ (2)	☐ (8)	☐ (9)	☐ (1)	☐ (2)	☐ (8)	☐ (9)
8. Medicamentos para la alergia	☐ (1)	☐ (2)	☐ (8)	☐ (9)	☐ (1)	☐ (2)	☐ (8)	☐ (9)
9. Medicamentos para la diarrea	☐ (1)	☐ (2)	☐ (8)	☐ (9)	☐ (1)	☐ (2)	☐ (8)	☐ (9)
10. Medicinas para el reuma	☐ (1)	☐ (2)	☐ (8)	☐ (9)	☐ (1)	☐ (2)	☐ (8)	☐ (9)
11. Medicinas para el corazón	☐ (1)	☐ (2)	☐ (8)	☐ (9)	☐ (1)	☐ (2)	☐ (8)	☐ (9)
12. Medicinas para la tensión arterial	☐ (1)	☐ (2)	☐ (8)	☐ (9)	☐ (1)	☐ (2)	☐ (8)	☐ (9)
13. Medicinas para el estómago y/o las alteraciones digestivas	☐ (1)	☐ (2)	☐ (8)	☐ (9)	☐ (1)	☐ (2)	☐ (8)	☐ (9)
14. Antidepresivos, estimulantes	☐ (1)	☐ (2)	☐ (8)	☐ (9)	☐ (1)	☐ (2)	☐ (8)	☐ (9)
15. Píldoras para no quedar embarazada (solo para mujeres)	☐ (1)	☐ (2)	☐ (8)	☐ (9)	☐ (1)	☐ (2)	☐ (8)	☐ (9)
16. Hormonas para la menopausia (solo para mujeres)	☐ (1)	☐ (2)	☐ (8)	☐ (9)	☐ (1)	☐ (2)	☐ (8)	☐ (9)
17. Medicamentos para adelgazar	☐ (1)	☐ (2)	☐ (8)	☐ (9)	☐ (1)	☐ (2)	☐ (8)	☐ (9)
18. Medicamentos para bajar el colesterol	☐ (1)	☐ (2)	☐ (8)	☐ (9)	☐ (1)	☐ (2)	☐ (8)	☐ (9)
19. Medicamentos para la diabetes	☐ (1)	☐ (2)	☐ (8)	☐ (9)	☐ (1)	☐ (2)	☐ (8)	☐ (9)
20. Medicamentos para el tiroides	☐ (1)	☐ (2)	☐ (8)	☐ (9)	☐ (1)	☐ (2)	☐ (8)	☐ (9)
21. Productos homeopáticos	☐ (1)	☐ (2)	☐ (8)	☐ (9)	☐ (1)	☐ (2)	☐ (8)	☐ (9)
22. Productos naturistas	☐ (1)	☐ (2)	☐ (8)	☐ (9)	☐ (1)	☐ (2)	☐ (8)	☐ (9)
23. Otros medicamentos	☐ (1)	☐ (2)	☐ (8)	☐ (9)	☐ (1)	☐ (2)	☐ (8)	☐ (9)

Q.- PRÁCTICAS PREVENTIVAS

Introducción P.88: Entrevistador/a, léale al informante: **"A continuación, le voy a preguntar sobre la vacunación de la gripe."**

88. ¿Se ha vacunado de la gripe en la última campaña?

Sí__________________ ▢ 1
No _________________ ▢ 2

Si P.88 = 2 → Ir a P.88c

88b. ¿Cuándo se vacunó?

Año ______________________ └┴┴┴┘
Mes ______________________ └┴┘

Ir a Introducción P.89

88c. ¿Cuándo fue la última vez que se vacunó?

Año 2019 __________________ ▢ 1 → **88c2**: Mes└┴┘
Año 2018 __________________ ▢ 2 → **88c3**: Mes└┴┘
Antes del año 2018 __________ ▢ 3
No me he vacunado nunca ____ ▢ 4
No sabe ____________________ ▢ 8
No contesta ________________ ▢ 9

Introducción P.89: Entrevistador/a, léale al informante: **"Ahora le voy a preguntar sobre su tensión arterial, su nivel de colesterol y su nivel de azúcar".**

89. ¿Le ha tomado la tensión alguna vez un profesional sanitario?

Sí___________ ▢ 1
No__________ ▢ 2
No sabe______ ▢ 8
No contesta___ ▢ 9

Si P.89= 2, 8, 9 → Ir a P.91

90. La última vez que le tomaron la tensión fue:

En los últimos 12 meses____________________☐ 1
Hace 1 año o más pero menos de 2 años_____☐ 2
Hace 2 años o más pero menos de 3 años____☐ 3
Hace 3 años o más pero menos de 5 años____☐ 4
Hace 5 años o más________________________☐ 5
No sabe___________________________________☐ 8
No contesta_______________________________☐ 9

91. ¿Le han medido alguna vez su nivel de colesterol en sangre?

Sí___________☐ 1
No__________☐ 2
No sabe______☐ 8
No contesta___☐ 9

Si P.91 = 2, 8, 9 → Ir a P.93

92. ¿Cuándo fue la última vez que le midieron su nivel de colesterol en sangre?

En los últimos 12 meses____________________☐ 1
Hace 1 año o más pero menos de 3 años_____☐ 2
Hace 3 años o más pero menos de 5 años____☐ 3
Hace 5 años o más________________________☐ 4
No sabe___________________________________☐ 8
No contesta_______________________________☐ 9

93. ¿Le ha medido alguna vez su nivel de azúcar en sangre un profesional sanitario?

Sí___________☐ 1
No__________☐ 2
No sabe______☐ 8
No contesta___☐ 9

Si P.93 = 2, 8, 9 → Ir a Introducción P.95

94. ¿Cuándo fue la última vez que un profesional sanitario le midió su nivel de azúcar en sangre?

En los últimos 12 meses__________________☐ 1
Hace 1 año o más pero menos de 3 años ___☐ 2
Hace 3 años o más pero menos de 5 años___☐ 3
Hace 5 años o más______________________☐ 4
No sabe_______________________________☐ 8
No contesta____________________________☐ 9

Introducción P.95: Entrevistador/a, léale al informante: **"Las siguientes preguntas hacen referencia a la prueba de sangre oculta en heces y a la colonoscopia."**

95. ¿Alguna vez le han hecho una prueba de sangre oculta en heces?

Sí___________☐ 1
No__________☐ 2
No sabe______☐ 8
No contesta___☐ 9

Si P.95 = 2, 8, 9 → Ir a P.98

96. ¿Cuándo fue la última vez que le hicieron una prueba de sangre oculta en heces?

En los últimos 12 meses________________☐ 1
Hace 1 año o más pero menos de 2 años__☐ 2
Hace 2 años o más pero menos de 3 años_☐ 3
Hace 3 años o más pero menos de 5 años_☐ 4
Hace 5 años o más____________________☐ 5
No sabe_____________________________☐ 8
No contesta__________________________☐ 9

98. ¿Le han realizado alguna vez una colonoscopia?

Sí___________☐ 1
No__________☐ 2
No sabe______☐ 8
No contesta___☐ 9

Si P.98 = 2, 8, 9 y el adulto seleccionado es mujer → Ir a Introducción P.100
Si P.98 = 2, 8, 9 y el adulto seleccionado es hombre → Ir a Introducción P.106

99. ¿Cuándo fue la última vez que le hicieron una colonoscopia?

En los últimos 12 meses ☐ 1
Hace 1 año o más pero menos de 5 años ☐ 2
Hace 5 años o más pero menos de 10 años ☐ 3
Hace 10 años o más ☐ 4
No sabe ☐ 8
No contesta ☐ 9

Si el adulto seleccionado es mujer → Ir a Introducción P.100
SI el adulto seleccionado es hombre → Ir a Introducción P.106

Introducción P.100: Entrevistador/a, léale al informante: **"Las siguientes preguntas hacen referencia a las pruebas de mamografía y de citología vaginal".**

100. ¿Le han hecho alguna vez una mamografía?

Sí ☐ 1
No ☐ 2
No sabe ☐ 8
No contesta ☐ 9

Si P.100 = 2, 8, 9 → Ir a P.103

101. ¿Cuándo fue la última vez que le hicieron una mamografía?

En los últimos 12 meses ☐ 1
Hace 1 año o más pero menos de 2 años ☐ 2
Hace 2 años o más pero menos de 3 años ☐ 3
Hace 3 años o más ☐ 4
No sabe ☐ 8
No contesta ☐ 9

103. ¿Le han hecho alguna vez una citología vaginal?

Sí ______________________________ ▢ 1
No _____________________________ ▢ 2
No sabe _________________________ ▢ 8
No contesta ______________________ ▢ 9

Si P.103 = 2, 8, 9 → Ir a Introducción P.106

104. ¿Cuándo fue la última vez que le hicieron una citología vaginal?

En los últimos 12 meses____________________1
Hace 1 año o más pero menos de 2 años______2
Hace 2 años o más pero menos de 3 años_____3
Hace 3 años o más pero menos de 5 años_____4
Hace 5 años o más________________________5
No sabe__________________________________8
No contesta_______________________________9

R.- NECESIDADES DE ATENCIÓN MÉDICA NO CUBIERTAS

Introducción P.106: Entrevistador/a, leále al informante: **"Por diferentes motivos puede tener que esperar para recibir atención médica e incluso puede no llegar a recibirla."**

106. En los últimos 12 meses, ¿alguna vez ha tardado en recibir o ha carecido de asistencia médica cuando lo necesitaba por una lista de espera demasiado larga?

Sí ☐ 1
No ☐ 2
No he necesitado asistencia médica ☐ 3
No sabe ☐ 8
No contesta ☐ 9

107. En los últimos 12 meses, ¿alguna vez ha tardado en recibir o ha carecido de asistencia médica cuando lo necesitaba por dificultades relacionadas con el transporte o la distancia?

Sí ☐ 1
No ☐ 2
No he necesitado asistencia médica ☐ 3
No sabe ☐ 8
No contesta ☐ 9

108. En los últimos 12 meses, ¿ha necesitado alguno de los siguientes tipos de atención sanitaria y no se lo pudo permitir por motivos económicos?

	Sí	No	No lo he necesitado	No sabe	No contesta
A. Atención médica	☐ (1)	☐ (2)	☐ (3)	☐ (8)	☐ (9)
B. Atención dental	☐ (1)	☐ (2)	☐ (3)	☐ (8)	☐ (9)
C. Algún medicamento que le habían recetado	☐ (1)	☐ (2)	☐ (3)	☐ (8)	☐ (9)
D. Atención de salud mental (Consulta al psicólogo, psiquiatra o psicoterapeuta, por ejemplo)	☐ (1)	☐ (2)	☐ (3)	☐ (8)	☐ (9)

MÓDULO DE DETERMINANTES DE LA SALUD

S.- CARACTERÍSTICAS FÍSICAS

Introducción P.109: Entrevistador/a, léale al informante: **"Ahora le voy a hacer unas preguntas sobre su talla y su peso."**

109. ¿Podría decirme cuánto mide, aproximadamente, sin zapatos?

|__|__|__| cm NS ☐ 998 NC ☐ 999

110. ¿Y cuánto pesa, aproximadamente, sin zapatos ni ropa?

|__|__|__| kg NS ☐ 998 NC ☐ 999

T.- ACTIVIDAD FÍSICA

Introducción P.111: Entrevistador/a, léale al informante: **"Ahora voy a hacerle unas preguntas sobre la actividad física que realiza."**

111. ¿Cuál de estas posibilidades describe mejor su actividad principal en el centro de trabajo, centro de enseñanza, hogar (labores domésticas)…?

Sentado/a la mayor parte de la jornada ☐ 1
De pie la mayor parte de la jornada sin efectuar grandes desplazamientos o esfuerzos ☐ 2
Caminando, llevando algún peso, efectuando desplazamientos frecuentes ☐ 3
Realizando tareas que requieren gran esfuerzo físico ☐ 4
No aplicable ☐ 5
No sabe ☐ 8
No contesta ☐ 9

112. ¿Cuál de estas posibilidades describe mejor la frecuencia con la que realiza alguna actividad física en su tiempo libre?

No hago ejercicio. El tiempo libre lo ocupo de forma casi completamente sedentaria (leer, ver la televisión, ir al cine, etc.) ______ ▢ 1
Hago alguna actividad física o deportiva ocasional (caminar o pasear en bicicleta, jardinería, gimnasia suave, actividades recreativas que requieren un ligero esfuerzo, etc.) ______ ▢ 2
Hago actividad física varias veces al mes (deportes, gimnasia, correr, natación, ciclismo, juegos de equipo, etc.) ______ ▢ 3
Hago entrenamiento deportivo o físico varias veces a la semana ______ ▢ 4
No sabe ______ ▢ 8
No contesta ______ ▢ 9

Introducción P.113: Entrevistador/a, léale al informante: **"Ahora me gustaría preguntarle por la forma de ir a los sitios a los que se desplaza habitualmente, por ejemplo, para ir a trabajar, a clase, para hacer la compra, etc."**

113. En una semana de actividad normal, ¿cuántos días camina al menos 10 minutos seguidos para desplazarse?

Número de días: └─┘

NS ▢ 8 NC ▢ 9

Si P.113 = 0, 8, 9 → Ir a P.115

114. Habitualmente, en uno de esos días, ¿cuánto tiempo camina para desplazarse?

Nota P.114: **Entrevistador/a, no se leen las opciones**. Marque la que más se ajuste a la respuesta del entrevistado.

De 10 a 29 minutos ______ ▢ 1
De 30 a 59 minutos ______ ▢ 2
Una hora o más, pero menos de 2 horas ______ ▢ 3
Dos horas o más, pero menos de 3 horas ______ ▢ 4
Tres horas o más ______ ▢ 5
No sabe ______ ▢ 8
No contesta ______ ▢ 9

115. En una semana de actividad normal, ¿cuántos días utiliza la bicicleta al menos durante 10 minutos para desplazarse?

Número de días: └─┘

NS ▢ 8 NC ▢ 9

Si P.115 = 0, 8, 9 → Ir a P.117

116. En uno de esos días, ¿cuánto tiempo utiliza habitualmente la bicicleta para desplazarse?

Nota P.116: **Entrevistador/a, no se leen las opciones**. Marque la que más se ajuste a la respuesta del entrevistado.

De 10 a 29 minutos________________________▢ 1
De 30 a 59 minutos________________________▢ 2
Una hora o más, pero menos de 2 horas__________▢ 3
Dos horas o más, pero menos de 3 horas_________▢ 4
Tres horas o más__________________________▢ 5
No sabe_______________________________▢ 8
No contesta____________________________▢ 9

Introducción P.117: **Entrevistador/a,** léale al informante: **"Las siguientes preguntas se refieren al ejercicio físico que realiza en su tiempo de ocio. No incluya el ejercicio declarado en las preguntas anteriores."**

117. En una semana de actividad normal, ¿cuántos días practica deporte, gimnasia, ciclismo, camina deprisa, etc., al menos 10 minutos seguidos?

Número de días: └─┘

NS ▢ 8 NC ▢ 9

Si P.117 = 0, 8, 9 → Ir a P.119

118. Y en total, ¿cuánto tiempo dedica a estas actividades en una semana normal?

└─┴─┘ **:** └─┴─┘ horas y minutos por semana

NS ▢ 98 NC ▢ 99

119. En una semana de actividad normal, ¿cuántos días realiza actividades específicamente destinadas a fortalecer sus músculos? Considere todas las actividades de este tipo aunque ya las haya tenido en cuenta en la pregunta anterior.

Número de días: |__|

NS ☐ 8   NC ☐ 9

119b. La última pregunta se refiere al tiempo que permanece sentado/a en un día normal. Incluya el tiempo @sentado/a en el trabajo, la casa, en clase, estudiando, leyendo y en el transporte, tiempo libre o viendo la Televisión. ¿Cuánto tiempo permaneció sentado/a en un día normal?

- Horas por día ________________ |_ _|
- Minutos por día________________ |_ _|
- No sabe ____________________ ☐ 98
- No contesta __________________ ☐ 99

U.- ALIMENTACIÓN

Introducción P.120: Entrevistador/a, léale al informante: **"Le voy a hacer unas preguntas sobre alimentación."**

120. ¿Con qué frecuencia consume los siguientes alimentos?

	Frecuencias de consumo							
**Alimentos**	Una o más veces al día	De 4 a 6 veces a la semana	Tres veces a la semana	Una o dos veces a la semana	Menos de 1 vez a la semana	Nunca	No sabe	No contesta
Fruta fresca (excluyendo zumos)__________	☐ 1 N° ☐	☐ 2	☐ 3	☐ 4	☐ 5	☐ 6	☐ 8	☐ 9
Carne (pollo, ternera, cerdo, cordero...)__________	☐ 1	☐ 2	☐ 3	☐ 4	☐ 5	☐ 6	☐ 8	☐ 9
Huevos__________	☐ 1	☐ 2	☐ 3	☐ 4	☐ 5	☐ 6	☐ 8	☐ 9
Pescado__________	☐ 1	☐ 2	☐ 3	☐ 4	☐ 5	☐ 6	☐ 8	☐ 9
Pasta, arroz, patatas__________	☐ 1	☐ 2	☐ 3	☐ 4	☐ 5	☐ 6	☐ 8	☐ 9
Pan, cereales__________	☐ 1	☐ 2	☐ 3	☐ 4	☐ 5	☐ 6	☐ 8	☐ 9
Verduras, ensaladas y hortalizas__________	☐ 1 N° ☐	☐ 2	☐ 3	☐ 4	☐ 5	☐ 6	☐ 8	☐ 9
Legumbres__________	☐ 1	☐ 2	☐ 3	☐ 4	☐ 5	☐ 6	☐ 8	☐ 9
Embutidos y fiambres__________	☐ 1	☐ 2	☐ 3	☐ 4	☐ 5	☐ 6	☐ 8	☐ 9
Productos lácteos (leche, queso, yogur)__________	☐ 1	☐ 2	☐ 3	☐ 4	☐ 5	☐ 6	☐ 8	☐ 9
Dulces (galletas, bollería, mermeladas, cereales con azúcar, caramelos)__________	☐ 1	☐ 2	☐ 3	☐ 4	☐ 5	☐ 6	☐ 8	☐ 9
Refrescos con azúcar __________	☐ 1	☐ 2	☐ 3	☐ 4	☐ 5	☐ 6	☐ 8	☐ 9
Comida rápida (pollo frito, bocadillos, pizzas, hamburguesas)__________	☐ 1	☐ 2	☐ 3	☐ 4	☐ 5	☐ 6	☐ 8	☐ 9
Aperitivos o comidas saladas de picar (patatas fritas, ganchitos, galletitas saladas)__________	☐ 1	☐ 2	☐ 3	☐ 4	☐ 5	☐ 6	☐ 8	☐ 9
Zumo natural de frutas o verduras__________	☐ 1 N° ☐	☐ 2	☐ 3	☐ 4	☐ 5	☐ 6	☐ 8	☐ 9

*Solo si P120.1 (Fruta fresca) = 2 y P120.15 (Zumo natural) = 2 → se pasa a la pregunta 120.D
*En otro caso → Ir a Introducción P.121

120.D Considerando ahora de manera conjunta su consumo de fruta fresca y zumos naturales a la semana, ¿toma a diario al menos uno de ellos?

Sí ☐ 1
No ☐ 2
No sabe ☐ 8
No contesta ☐ 9

Si P120.D = 2, 8, 9 → Ir a Introducción P.121

120.E ¿Cuántas piezas de fruta o vasos de zumo natural consume a diario?

*

NS ☐ 98 NC ☐ 99

V.- CONSUMO DE TABACO Y EXPOSICIÓN AL HUMO DE TABACO

Introducción P.121: Entrevistador/a, léale al informante: **"Las siguientes preguntas se refieren al consumo de tabaco y a la exposición al humo de tabaco."**

121. ¿Podría decirme si fuma? No tenga en cuenta los cigarrillos electrónicos u otros dispositivos electrónicos similares.

Sí, fumo a diario ☐ 1
Sí fumo, pero no a diario ☐ 2
No fumo actualmente pero he fumado antes ☐ 3
No fumo ni he fumado nunca de manera habitual ☐ 4
No sabe ☐ 8
No contesta ☐ 9

Si P.121= 2,3,8,9 → Ir a P.124a

Si P.121= 4 → Ir a P124a

123. Por término medio, ¿cuántos cigarrillos fuma usted al día?

Número de cigarrillos: |__|__| NS ▢ 98 NC ▢ 99

124. ¿A qué edad empezó a fumar?

Edad en años: |_ _| NS ▢ 98 NC ▢ 99

Si P.121= 1 → Ir a P.124b

124a. Aunque ahora no fume a diario, ¿Alguna vez ha fumado a diario durante al menos un año?

Sí________________________________ ▢ 1
No________________________________ ▢ 2
No sabe___________________________ ▢ 8
No contesta_______________________ ▢ 9

Si P.124a= 2 ,8 ,9 → Ir a P.126

Si el adulto seleccionado ha contestado P.121= 1, presentar enunciado P124.B1

Si el adulto seleccionado ha contestado P.124a=1, presentar enunciado P124.B2

124b1. ¿Cuántos años lleva fumando a diario?

124b2. ¿Durante cuántos años fumó a diario?

Años: |_ _| NS ▢ 98 NC ▢ 99

126. ¿Con qué frecuencia está expuesto/a al humo del tabaco en lugares cerrados? Considere solo aquellas situaciones en las que son otras personas las que están fumando.

Todos los días ☐ 1 → **Desplegar 126a**

→ **126a:** Menos de una hora al día ☐ 1
Entre 1 y 5 horas al día ☐ 2
Más de 5 horas al día ☐ 3

Al menos una vez a la semana
(pero no todos los días) ☐ 2
Menos de una vez por semana ☐ 3
Nunca o casi nunca ☐ 4
No sabe ☐ 8
No contesta ☐ 9

126b. ¿Emplea cigarrillos electrónicos o aparatos electrónicos similares (ej: shisha electrónica, pipa electrónica…)?

Sí ☐ 1
No actualmente, pero sí lo he hecho con anterioridad ☐ 2
No, nunca ☐ 3
No sabe ☐ 8
No contesta ☐ 9

Si P.126b=2,3,8,9 → Ir a P.127

126c. ¿Con qué frecuencia emplea cigarrillos electrónicos o aparatos electrónicos similares (ej: shisha electrónica, pipa electrónica…)?

Diariamente ☐ 1
Ocasionalmente ☐ 2
No sabe ☐ 8
No constesta ☐ 9

W.- CONSUMO DE ALCOHOL

Introducción P.127: Entrevistador/a, léale al informante: **"Las siguientes preguntas se refieren al consumo de bebidas alcohólicas durante los últimos 12 meses."**

127. Durante los últimos 12 meses, ¿con qué frecuencia ha tomado bebidas alcohólicas de cualquier tipo (es decir, cerveza, vino, licores, bebidas destiladas y combinados u otras bebidas alcohólicas)?

A diario o casi a diario.. ☐ 01
5-6 días por semana.. ☐ 02
3-4 días por semana.. ☐ 03
1-2 días por semana.. ☐ 04
2-3 días en un mes... ☐ 05
Una vez al mes.. ☐ 06
Menos de una vez al mes.. ☐ 07
No en los últimos 12 meses, he dejado de tomar alcohol.............................. ☐ 08
Nunca o solamente unos sorbos para probarlo a lo largo de toda la vida...... ☐ 09
No sabe.. ☐ 98
No contesta.. ☐ 99

Si P.127 = 05, 06, 07 → Ir a Introducción P.129

Si P.127 = 08, 09, 98, 99 → Ir a Introducción P.130

128. Durante los últimos 12 meses, en una semana en que desarrolló usted su actividad habitual, ¿cuántas bebidas que contengan alcohol consumió a lo largo de la semana?

	Cervezas con alcohol Sí ☑ NS ☐ No ☐ NC ☐	Vinos, Cava Sí ☐ NS ☐ No ☑ NC ☐	Vermuts, fino, jerez Sí ☐ NS ☐ No ☑ NC ☐	Licores, anís, pacharán Sí ☑ NS ☐ No ☐ NC ☐	Whisky, coñac, combinados ... Sí ☐ NS ☐ No ☑ NC ☐	Bebidas 'locales' sidra, carajillo Sí ☐ NS ☐ No ☐ NC ☑
Lunes	0 Cervezas	Vasos,copas	Copas	0 Copas	Copas	Vasos, copas
Martes	0 Cervezas	Vasos,copas	Copas	0 Copas	Copas	Vasos, copas
Miércoles	0 Cervezas	Vasos,copas	Copas	0 Copas	Copas	Vasos, copas
Jueves	0 Cervezas	Vasos,copas	Copas	0 Copas	Copas	Vasos, copas
Viernes	0 Cervezas	Vasos,copas	Copas	0 Copas	Copas	Vasos, copas
Sábado	0 Cervezas	Vasos,copas	Copas	0 Copas	Copas	Vasos, copas
Domingo	0 Cervezas	Vasos,copas	Copas	0 Copas	Copas	Vasos, copas

Si el adulto seleccionado es hombre, presentar enunciado P129.A
Si el adulto seleccionado es mujer, presentar enunciado P129.B

129.A Durante los últimos 12 meses, ¿con qué frecuencia ha tomado 6 o más bebidas estándar en una misma ocasión? (Por "ocasión" entendemos tomar las bebidas en un intervalo aproximado de cuatro a seis horas)

129.B Durante los últimos 12 meses, ¿con qué frecuencia ha tomado 5 o más bebidas estándar en una misma ocasión? (Por "ocasión" entendemos tomar las bebidas en un intervalo aproximado de cuatro a seis horas)

- A diario o casi a diario........................ ☐ 01
- De 5 a 6 días por semana.................... ☐ 02
- De 3 a 4 días por semana.................... ☐ 03
- De 1 a 2 días por semana.................... ☐ 04
- De 2 a 3 días en un mes...................... ☐ 05
- Una vez al mes.................................... ☐ 06
- Menos de una vez al mes.................... ☐ 07
- No en los últimos 12 meses................ ☐ 08
- Nunca en toda mi vida......................... ☐ 09
- No sabe.. ☐ 98
- No contesta.. ☐ 99

X.- APOYO SOCIAL

Introducción P.130: Entrevistador/a, léale al informante: **"Las siguientes preguntas hacen referencia a sus relaciones sociales."**

130. En caso de tener un problema personal grave de cualquier tipo, ¿con cuántas personas cercanas a usted podría contar?

- Ninguna ________________ ☐ 1
- 1 o 2 personas ____________ ☐ 2
- De 3 a 5 personas _________ ☐ 3
- Más de 5 personas ________ ☐ 4
- No sabe ________________ ☐ 8
- No contesta ______________ ☐ 9

131. ¿En qué medida se interesan otras personas por lo que a usted le pasa?

Mucho________________ ▢ 1
Algo__________________ ▢ 2
Ni mucho ni poco________ ▢ 3
Poco__________________ ▢ 4
Nada__________________ ▢ 5
No sabe_______________ ▢ 8
No contesta____________ ▢ 9

132. ¿En qué medida le resultaría fácil obtener ayuda de los vecinos en caso de necesidad?

Muy fácil___________ ▢ 1
Fácil______________ ▢ 2
Es posible__________ ▢ 3
Difícil______________ ▢ 4
Muy difícil__________ ▢ 5
No sabe___________ ▢ 8
No contesta________ ▢ 9

Y.- CUIDADO A OTRAS PERSONAS CON PROBLEMAS DE SALUD

Introducción P.133: Entrevistador/a, léale al informante: **"Las siguientes preguntas tratan de conocer si usted dedica tiempo al cuidado de alguna persona con problemas de salud."**

133. ¿Cuida, al menos una vez a la semana, de alguna persona mayor o de alguien que tenga una dolencia crónica? No lo considere si forma parte de su trabajo.

Sí______________________ ▢ 1
No_____________________ ▢ 2
No sabe_________________ ▢ 8
No contesta______________ ▢ 9

Si P.133 <> 1 → Ir a INGRESOS

134. La persona o personas a las que cuida son:

Familiares____________ ▢ 1
Otras personas________ ▢ 2
No sabe______________ ▢ 8
No contesta___________ ▢ 9

135. En total, ¿cuántas horas a la semana dedica al cuidado de esta/s persona/s?

Menos de 10 horas a la semana________________ ▢ 1
10 horas o más a la semana pero menos de 20____ ▢ 2
20 horas a la semana o más___________________ ▢ 3
No sabe__________________________________ ▢ 8
No contesta________________________________ ▢ 9

Z.- INGRESOS

Introducción P.136: Entrevistador/a, léale al informante: **"Para poder hacer comparaciones entre los distintos tipos de problemas de salud de las familias, es necesario conocer unos datos muy básicos sobre el nivel de ingresos de la familia.**

"A continuación le voy a preguntar por los ingresos regulares del hogar. No incluya los ingresos de las personas residentes empleadas en el hogar ni de los huéspedes fijos."

136. De los siguientes tipos de ingresos que le voy a leer, ¿podría decirme cuáles de ellos reciben usted y los demás miembros de su hogar?
Debe considerar las fuentes de cada miembro del hogar y las conjuntas.

Ingresos del trabajo (por cuenta propia o ajena) ______________ ▢
Prestación y subsidios por desempleo ______________________ ▢
Pensión por jubilación o viudedad _________________________ ▢
Pensión por invalidez o incapacidad ________________________ ▢
Prestaciones económicas por hijo a cargo u otras prestaciones económicas como ayudas a la familia... ____________________ ▢
Prestaciones o subvenciones relacionadas con la vivienda_______ ▢
Prestaciones o subvenciones relacionadas con la educación _____ ▢
Otros ingresos regulares / Otro subsidio o prestación social regular ____ ▢
Ninguna fuente de ingresos ______________________________ ▢
No sabe ____ ▢
No contesta____ ▢

Si entre las respuestas 1 y 8 hay una única respuesta marcada → Ir a **P.138B**
Si entre las respuestas 1 y 8 hay más de una respuesta marcada→ Ir a **P.137**
Si P.136 = 98 (“No sabe”) → Ir a **P138.A**
Si P.136 = 09, 99 (“Ninguna fuente de ingresos” o “No contesta”) → **Idioma**

137. De estas fuentes de ingresos monetarios del hogar citadas, ¿cuál diría que es la principal?

Ir a **P.138C**

138A. Aunque no pueda en este momento especificar las fuentes de ingresos, ¿podría decir cuál es aproximadamente el ingreso mensual neto de todo el hogar (es decir, sumando todas las fuentes y descontando las retenciones por impuestos, Seguridad Social, etc.)?

138B. Pensando en la fuente de ingresos que ha mencionado, ¿podría decir cuál es aproximadamente el ingreso mensual neto de todo el hogar (es decir, sumando todas las fuentes y descontando las retenciones por impuestos, Seguridad Social, etc.)?

138C. Pensando en las fuentes de ingresos que ha mencionado, ¿podría decir cuál es aproximadamente el ingreso mensual neto de todo el hogar (es decir, sumando todas las fuentes y descontando las retenciones por impuestos, Seguridad Social, etc.)?

138D. ¿Podría decir cuál es el importe mensual aproximado de los ingresos del hogar, (es decir, sumando todas las fuentes y descontando las retenciones por impuestos, Seguridad Social, etc.)?

Cantidad |___|___|___|___|___|___| euros

No sabe__________________________ ▢ 999998

No contesta_______________________ ▢ 999999

139. Si desconoce el valor exacto de los ingresos del hogar, ¿podría decirme cuál de los intervalos siguientes representa mejor el ingreso mensual neto de todo su hogar, tras las deducciones por los impuestos, Seguridad Social, etc.?

- Menos de 600 euros ☐ 01
- De 600 a menos de 830 euros ☐ 02
- De 830 a menos de 1100 euros ☐ 03
- De 1100 a menos de 1.360 euros ☐ 04
- De 1.360 a menos de 1.650 euros ☐ 05
- De 1.650 a menos de 1.950 euros ☐ 06
- De 1.950 a menos de 2.300 euros ☐ 07
- De 2.300 a menos de 2.900 euros ☐ 08
- De 2.900 a menos de 3.800 euros ☐ 09
- De 3.800 a menos de 4.700 euros ☐ 10
- De 4.700 a menos de 6.300 euros ☐ 11
- De 6.300 euros en adelante ☐ 12
- No sabe ☐ 98
- No contesta ☐ 99

INFORMACIÓN SOBRE LA ENTREVISTA

141. Idioma

Entrevistador/a, anote el idioma empleado principalmente por el informante para responder al Cuestionario de Adulto:

- Castellano ☐ 1
- Catalán ☐ 2
- Valenciano ☐ 3
- Euskera ☐ 4
- Gallego ☐ 5
- Inglés ☐ 6
- Otro ☐ 7
