# Supplementary material for: Temporal Evolution and Associated Factors of Adherence to Mammography Screening among Women in Spain: Results from Two National Health Surveys (2017–2020)
Source: Healthcare (Basel). 2023 Nov 9;11(22):2934. doi: 10.3390/healthcare11222934 (PMC10671473; doi:10.3390/healthcare11222934)
Supplement: Supplementary file 1 [file healthcare-11-02934-s001.zip › Questionnaire Spanish National Health Survey 2017.pdf]

# ENCUESTA NACIONAL DE SALUD 2017

## *CUESTIONARIO DE ADULTOS*

**Entrevistador/a:** Recuerde que la persona entrevistada debe ser la persona adulta seleccionada salvo en los casos excepcionales recogidos en el manual.

**Entrevistador/a,** léale al informante: **"Las siguientes preguntas conciernen únicamente a la persona adulta seleccionada, es decir, a:**

**Nombre Apellido1 Apellido2 (del adulto seleccionado)**

**Sexo:__________**

**Edad: _ _ _ años"**

**Número de orden: _ _**

**Proxy_0. ¿El informante es la persona seleccionada?**

- Sí ___________ ▢ 1 → Ir a Introducción P.1
- No___________ ▢ 2

**Proxy_1. ¿Cuál es el motivo por el que la persona seleccionada no puede facilitar personalmente sus datos?**

- La persona seleccionada está ingresada en un sanatorio, hospital, etc., a causa de una enfermedad____________________________ ▢ 1
- La persona seleccionada está incapacitada para contestar por discapacidad, enfermedad grave, etc.________________________ ▢ 2
- La persona seleccionada está incapacitada para contestar por causa del idioma___________________________________________ ▢ 3

**Proxy_2. ¿Es miembro del hogar el informante?**

- Sí__________ ▢ 1 → Listado de miembros del hogar: | _ | (proxy_2b) → Ir a flujo antes de Proxy_5
- No _________ ▢ 2

**Proxy_3. Nombre del informante: ____________________________**

**Proxy_3b. Sexo del informante:** ▢ 1 Hombre ▢ 2 Mujer

**Proxy_4. Edad del informante:** └┴┘

**Proxy_5. ¿Cuál es la relación del informante con la persona seleccionada?**

- Cónyuge o pareja ____________________ ▢ 1
- Hijo/Hija ____________________ ▢ 2
- Padre/madre ____________________ ▢ 3
- Hermano/hermana ____________________ ▢ 4
- Otros familiares ____________________ ▢ 5
- Servicios sociales ____________________ ▢ 6
- Voluntarios ____________________ ▢ 7
- Otra relación ____________________ ▢ 8

## E.- CARACTERÍSTICAS DEMOGRÁFICAS DE LA PERSONA ADULTA SELECCIONADA

**Introducción P.1: Entrevistador/a**, léale al informante: **"A continuación voy a hacerle unas preguntas sobre su nacionalidad y su estado civil".**

**1. ¿Cuál es su país de nacimiento?**

- España ____________________ ▢ 1
- Extranjero ____________________ ▢ 2 → **1b-** Seleccionar país
- No sabe ____________________ ▢ 8
- No contesta ____________________ ▢ 9

Si P.1=2 ➔ Se activa la correspondiente 1b con un listado de países para seleccionar.

**2. ¿Y cuál es su nacionalidad?**

- Española ____________________ ▢
- Extranjera ____________________ ▢ → **2b-** Seleccionar país
- No sabe ____________________ ▢
- No contesta ____________________ ▢

Si P.1 = 1 → Ir a flujo previo a P.4

**3. ¿Cuántos años lleva residiendo en España?**

Nº de años └┴┘ NS ▢ 98 NC ▢ 99

**4. De las siguientes opciones, ¿cuál describe su situación actual?**

- Conviviendo con su cónyuge __________ ▢ 1
- Conviviendo con una pareja de hecho __ ▢ 2
- No conviviendo en pareja _____________ ▢ 3
- No sabe______________________________ ▢ 8
- No contesta__________________________ ▢ 9

**4b. ¿Cuál es su estado civil legal?**

- @Soltero/a___________________ ▢ 1
- @Casado/a __________________ ▢ 2
- @Viudo/a____________________ ▢ 3
- @Separado/a legalmente ______ ▢ 4
- @Divorciado/a________________ ▢ 5
- No sabe _____________________ ▢ 8
- No contesta __________________ ▢ 9

**5. Por favor, detalle a continuación cuál es el mayor nivel de estudios que ha obtenido: ____________________________**

**Entrevistador/a, codifique el nivel de estudios declarado por el adulto seleccionado en el literal anterior:**

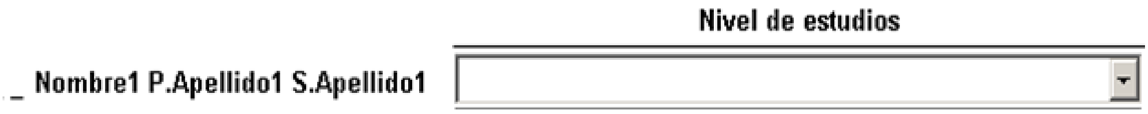


**Las opciones que aparecen en el desplegable son:**

**No sabe leer o escribir (analfabetos)**____ 01
**Estudios primarios incompletos (ha asistido menos de 5 años a la escuela)**__ 02
**Educación primaria completa**____ 10
**Primera etapa de educación secundaria sin título de graduado en ESO y equivalentes (EGB, Bachillerato elemental)**____ 21
**Primera etapa de educación secundaria con título de graduado en ESO y similares (EGB, Bachillerato elemental)**____ 22
**Certificados de profesionalidad de nivel 1 y similares**____ 23
**Certificados de profesionalidad de nivel 2 y similares**____ 24
**Bachillerato y similares (BUP, COU, PREU)**____ 32
**Enseñanzas de formación profesional de grado medio (incluye artes plásticas, diseño, deportivas y similares)**____33
**Enseñanzas profesionales de música y danza y similares**____ 34
**Certificado de las escuelas oficiales de idiomas de nivel avanzado y similares**____ 35
**Formación profesional básica**____ 38
**Certificados de profesionalidad de nivel 3; programas cortos que requieren segunda etapa de secundaria y similares**____ 41
**Enseñanzas de formación profesional de grado superior (incluye artes plásticas, diseño, deportivas y equivalentes)**____ 51
**Títulos propios universitarios que precisan del título de bachiller, de duración igual o superior a 2 años**____ 52
**Grados universitarios de 240 créditos ECTS (plan Bolonia) y equivalentes**___ 61
**Diplomados universitarios y equivalentes**____ 62
**Títulos propios universitarios de experto o especialista de menos de 60 créditos ECTS (plan Bolonia) cuyo acceso requiera ser titulado universitario**_ 63
**Grados universitarios de más de 240 créditos ECTS (plan Bolonia) y equivalentes** ____ 71
**Licenciados universitarios y equivalentes**____ 72
**Másteres oficiales universitarios y equivalentes**____ 73
**Especialidades en Ciencias de la Salud por el sistema de residencia o similares (MIR y equivalentes)**____ 74
**Títulos propios universitarios de máster (maestrías) de 60 o más créditos ECTS (plan Bolonia) cuyo acceso requiera ser titulado universitario**____ 75
**Doctorado universitario**____ 81
**No contesta**____ 99

# F.- RELACIÓN DE LA PERSONA ADULTA SELECCIONADA CON LA ACTIVIDAD ECONÓMICA

**Introducción P.6**: **Entrevistador/a,** léale al informante: **"Ahora voy a hacerle unas preguntas sobre su relación con la actividad económica".**

Los flujos de este módulo están condicionados a la respuesta de la pregunta 11 del Cuestionario de Hogar para el adulto seleccionado.

Si P.11 (C. Hogar) = 1 → Ir a P.13
Si P.11 (C. Hogar) = 2 → Ir a P.11
Si P.11 (C. Hogar) = 4, 6 ó 7 → Ir a P.12
Si P.11 (C. Hogar) = 3 ó 5 → Ir a P.6
Si P.11 (C. Hogar) = 8 → Ir a Módulo G

**6. ¿Percibe alguna pensión contributiva?**

- Sí, por cotización propia ________________ ☐ 1 → Ir a P.18b
- Sí, por cotización de otra persona ________ ☐ 2 → Ir a P.8
  (pensiones de viudedad, orfandad, etc.)
- Sí, por ambos tipos de cotización________ ☐ 3 → Ir a P.18b
- No __________________________________ ☐ 4
- No sabe _____________________________ ☐ 8
- No contesta __________________________ ☐ 9

**7. ¿Ha trabajado antes?**

- Sí ___________ ☐ 1
- No___________ ☐ 2
- No sabe _______ ☐ 8
- No contesta ___ ☐ 9

Si P.7 = 1 y P.6 = 4, 8 ó 9 → Ir a P.18b
Si P.7 = 2, 8 ó 9 y P.6 = 4, 8 ó 9 → Ir a Módulo G

**8. ¿Cuál era la actividad de la empresa u organización en la que trabajó en su último empleo la persona que generó la pensión?**

**Actividad:** ____________________

**Código de actividad** |_|_|_|

**9. ¿Cuál era la ocupación, profesión u oficio que desempeñó en su último empleo la persona que generó la pensión?**

**Profesión:** ____________________

**Código de ocupación** └─┴─┴─┘

**10. ¿Cuál era la situación profesional, en su último empleo, de la persona que generó la pensión?**

- Asalariado/a (a sueldo, comisión, jornal...) __________ ☐ 1
- Empresario/a o profesional con asalariados __________ ☐ 2
- Empresario/a sin asalariados o trabajador/a independiente ____ ☐ 3
- Ayuda familiar (sin remuneración reglamentada en la empresa o negocio de un familiar) __________ ☐ 4
- Miembro de una cooperativa __________ ☐ 5
- Otra situación __________ ☐ 6
- No sabe __________ ☐ 8
- No contesta __________ ☐ 9

Ir a Módulo G

**11. ¿Cuánto tiempo lleva en desempleo?**

- No ha trabajado nunca __________ ☐ 1 → Ir a Módulo G
- Menos de 6 meses __________ ☐ 2 → Ir a P.18b
- De 6 meses a menos de 1 año __________ ☐ 3 → Ir a P.18b
- De 1 año a menos de 2 años __________ ☐ 4 → Ir a P.18b
- Más de 2 años __________ ☐ 5 → Ir a P.18b
- No sabe __________ ☐ 8 → Ir a P.18b
- No contesta __________ ☐ 9 → Ir a P.18b

**12. Aunque actualmente no esté trabajando, ¿ha trabajado alguna vez anteriormente?**

- Sí __________ ☐ 1 → Ir a P.18b
- No __________ ☐ 2 → Ir a Módulo G
- No sabe __________ ☐ 8 → Ir a Módulo G
- No contesta ____ ☐ 9 → Ir a Módulo G

**13. ¿Qué tipo de contrato o relación laboral tiene?**

- @Asalariado/a (a sueldo, comisión, jornal...)_________ ▢

  Señale el tipo de contrato:
  - @Funcionario/a _________ ▢ 01 → Ir a P.15
  - Duración indefinida_______ ▢ 02 → Ir a P.15
  - Temporal _____________ ▢ 03 → Ir a P.14a
  - Verbal o sin contrato ____ ▢ 04 → Ir a P.14b

- @Empresario/a o profesional con asalariados _______ ▢ 05 → Ir a P.15
- @Empresario/a sin asalariados o @trabajador/a independiente __________________________________ ▢ 06 → Imputar P.15=1 e ir a P.16
- Ayuda familiar (sin remuneración reglamentada en la empresa o negocio de un familiar) ___________ ▢ 07 → Ir a P.15
- Miembro de una cooperativa ______________________ ▢ 08 → Ir a P.15
- Otra situación___________________________________ ▢ 09 → Ir a P.15
- No sabe _______________________________________ ▢ 98 → Ir a P.15
- No contesta ___________________________________ ▢ 99 → Ir a P.15

La subpregunta “Señale el tipo de contrato” está por defecto deshabilitada y se habilita al seleccionar la opción “Asalariado/a (a sueldo, comisión, jornal...)”.

**14a. ¿Cuál es la duración de su contrato?**

- Menor de 6 meses______________________ ▢ 1
- De 6 meses a menos de 1 año ___________ ▢ 2
- De 1 año a menos de 2 años_____________ ▢ 3
- De 2 años o más _______________________ ▢ 4
- Sin duración definida____________________ ▢ 5
- No sabe _______________________________ ▢ 8
- No contesta ____________________________ ▢ 9

**14b. ¿Cuál es la duración de su relación laboral?**

- Menor de 6 meses______________________ ▢ 1
- De 6 meses a menos de 1 año ___________ ▢ 2
- De 1 año a menos de 2 años_____________ ▢ 3
- De 2 años o más _______________________ ▢ 4
- Sin duración definida____________________ ▢ 5
- No sabe _______________________________ ▢ 8
- No contesta ____________________________ ▢ 9

**15. ¿Tiene trabajadores a su cargo?**

- No ________________________________ ▢ 1
- Sí, de 1 a 4 personas ________________ ▢ 2
- Sí, de 5 a 10 personas _______________ ▢ 3
- Sí, de 11 a 20 personas ______________ ▢ 4
- Sí, más de 20 personas ______________ ▢ 5
- No sabe____________________________ ▢ 8
- No contesta_________________________ ▢ 9

**16. Su trabajo principal actual, ¿es a tiempo completo o a tiempo parcial?**

Nota P.16: No se leen las opciones.

- A tiempo completo ___________________ ▢ 1
- A tiempo parcial _____________________ ▢ 2
- No sabe____________________________ ▢ 8
- No contesta_________________________ ▢ 9

**17. ¿Qué tipo de jornada tiene habitualmente?**

- Jornada partida ______________________ ▢ 01
- Jornada continua_____________________ ▢
    - Mañana _______________ ▢ 02
    - Tarde_________________ ▢ 03
    - Noche ________________ ▢ 04
- Jornada reducida_____________________ ▢ 05
- Turnos ____________________________ ▢ 06
- Jornada irregular o variable según los días_ ▢ 07
- Otro tipo___________________________ ▢ 08
- No sabe ___________________________ ▢ 98
- No contesta ________________________ ▢ 99

Las opciones de “Mañana”, “Tarde” y “Noche” solo deben aparecer cuando se marque la opción “Jornada continua”.

**18a. ¿Cuál es la actividad de la empresa u organización en la que trabaja actualmente?**

**Actividad:** ____________________

**Código de actividad** |_|_|_|_|

**18b. ¿Cuál era la actividad de la empresa u organización en la que trabajó en su último empleo?**

**Actividad:** ____________________

**Código de actividad** |_|_|_|_|

Ir a P.19b

**19a. ¿Cuál es la ocupación, profesión u oficio que desempeña en su empleo?**

**Profesión:** ____________________

**Código de ocupación** |_|_|_|_| → Ir a Módulo G

**19b. ¿Cuál era la ocupación, profesión u oficio que desempeñó en su último empleo?**

**Profesión:** ____________________

**Código de ocupación** |_|_|_|_|

**20. ¿Cuál era la situación profesional en la ocupación que desempeñó en su último empleo?**

- @Asalariado/a (a sueldo, comisión, jornal...) __________________ ▢ 1
- @Empresario/a o profesional con asalariados _________________ ▢ 2
- @Empresario/a sin asalariados o @trabajador/a independiente __ ▢ 3
- Ayuda familiar (sin remuneración reglamentada en la empresa o negocio de un familiar) ____________________________________ ▢ 4
- Miembro de una cooperativa_____________________________ ▢ 5
- Otra situación ____________________________________________ ▢ 6
- No sabe_________________________________________________ ▢ 8
- No contesta______________________________________________ ▢ 9

*MÓDULO DE ESTADO DE SALUD*

## G.- ESTADO DE SALUD

**Introducción P.21: Entrevistador/a,** léale al informante: "**A continuación le voy a preguntar sobre su salud".**

**21. En los últimos doce meses, ¿diría que su estado de salud ha sido muy bueno, bueno, regular, malo, muy malo?**

Nota P.21: No se leen las opciones.

- Muy bueno______________________________ ▢ 1
- Bueno _________________________________ ▢ 2
- Regular ________________________________ ▢ 3
- Malo___________________________________ ▢ 4
- Muy malo_______________________________ ▢ 5

**22. ¿Tiene alguna enfermedad o problema de salud crónicos o de larga duración? (Entendemos por larga duración si el problema de salud o enfermedad ha durado o se espera que dure 6 meses o más)**

1. Sí ▢ 1
2. No ▢ 2
3. No sabe ▢ 8
4. No contesta ▢ 9

**23. Durante al menos los últimos 6 meses, ¿en qué medida se ha visto @limitado/a debido a un problema de salud para realizar las actividades que la gente habitualmente hace?**

- Gravemente @limitado/a ______________________ ▢ 1
- @Limitado/a, pero no gravemente ______________ ▢ 2
- Nada @limitado/a ____________________________ ▢ 3
- No sabe ____________________________________ ▢ 8
- No contesta _________________________________ ▢ 9

Si P.23 = 3, 8 ó 9 → Ir a P25

**24. ¿Qué tipo de problema es la causa de su dificultad para realizar las actividades que la gente habitualmente hace?**

- Físico ____________ ▢ 1
- Mental ___________ ▢ 2
- Ambos ___________ ▢ 3
- No sabe __________ ▢ 8
- No contesta ______ ▢ 9

**25. A continuación le voy a leer una lista con una serie de enfermedades o problemas de salud. ¿Padece o ha padecido alguna vez alguna de ellas?**

Nota P.25: **Entrevistador/a**, léale al informante las enfermedades que se relacionan una a una anotando la respuesta que proceda.

| | 25.a ¿Alguna vez ha padecido ...? | 25.b ¿La ha padecido en los últimos 12 meses? | 25.c ¿Le ha dicho un médico que la padece? |
|---|---|---|---|
| Tensión alta | ○ SI (1) ○ NS(8)<br>○ NO (2) ○ NC (9) | ○ SI (1) ○ NS(8)<br>○ NO (2) ○ NC (9) | ○ SI (1) ○ NS(8)<br>○ NO (2) ○ NC (9) |
| Infarto de miocardio | ○ SI (1) ○ NS(8)<br>○ NO (2) ○ NC (9) | ○ SI (1) ○ NS(8)<br>○ NO (2) ○ NC (9) | ○ SI (1) ○ NS(8)<br>○ NO (2) ○ NC (9) |
| Angina de pecho, enfermedad coronaria | ○ SI (1) ○ NS(8)<br>○ NO (2) ○ NC (9) | ○ SI (1) ○ NS(8)<br>○ NO (2) ○ NC (9) | ○ SI (1) ○ NS(8)<br>○ NO (2) ○ NC (9) |
| Otras enfermedades del corazón | ○ SI (1) ○ NS(8)<br>○ NO (2) ○ NC (9) | ○ SI (1) ○ NS(8)<br>○ NO (2) ○ NC (9) | ○ SI (1) ○ NS(8)<br>○ NO (6) ○ NC (9) |

Lista de Enfermedades que deben aparecer en la pregunta:

| |
|---|
| 1. Tensión alta |
| 2. Infarto de miocardio |
| 3. Angina de pecho, enfermedad coronaria |
| 4. Otras enfermedades del corazón |
| 5. Varices en las piernas |
| 6. Artrosis (excluyendo artritis) |
| 7. Dolor de espalda crónico (cervical) |
| 8. Dolor de espalda crónico (lumbar) |
| 9. Alergia crónica, como rinitis, conjuntivitis o dermatitis alérgica, alergia alimentaria o de otro tipo (asma alérgica excluida) |
| 10. Asma (incluida asma alérgica) |
| 11. Bronquitis crónica, enfisema, enfermedad pulmonar obstructiva crónica (EPOC) |
| 12. Diabetes |
| 13. Úlcera de estómago o duodeno |
| 14. Incontinencia urinaria o problemas de control de la orina |
| 15. Colesterol alto |
| 16. Cataratas |
| 17. Problemas crónicos de piel |
| 18. Estreñimiento crónico |
| 19. Cirrosis, disfunción hepática |
| 20. Depresión |
| 21. Ansiedad crónica |
| 22. Otros problemas mentales |
| 23. Ictus (embolia, infarto cerebral, hemorragia cerebral) |
| 24. Migraña o dolor de cabeza frecuente |
| 25. Hemorroides |
| 26. Tumores malignos |
| 27. Osteoporosis |
| 28. Problemas de tiroides |
| 29. Problemas de riñón |
| 30. Problemas de próstata (solo hombres) |
| 31. Problemas del periodo menopáusico (solo mujeres) |
| 32. Lesiones o defectos permanentes causados por un accidente |

| 33. ¿Ha padecido alguna otra enfermedad crónica? ↓ |
|---|
| 33.1........................................................................................ |
| 33.2....................................................................................... |
| 33.3........................................................................................ |

## H.- ACCIDENTALIDAD

**26. Durante los últimos 12 meses, ¿ha tenido alguno de los siguientes tipos de accidente en el que haya resultado @herido/a o @lesionado/a?**

**Nota P.26:** Se incluyen los casos de intoxicación, picaduras de insectos y las heridas provocadas por animales. Se excluyen los actos intencionados por parte de otra persona.

| | Sí | No | No sabe | No contesta |
|---|---|---|---|---|
| **A. Accidente de tráfico** | ☐ (1) | ☐ (2) | ☐ (8) | ☐ (9) |
| **B. Accidente en casa** | ☐ (1) | ☐ (2) | ☐ (8) | ☐ (9) |
| **C. Accidente durante su tiempo libre** | ☐ (1) | ☐ (2) | ☐ (8) | ☐ (9) |

Si P26.A=1 o P26.B=1 o P26.C=1 → Ir a P.27
En otro caso, ir a Introducción P.28

**27. ¿Recibió atención médica como consecuencia de este/os accidente/s?**

**Nota P.27:** No se admite respuesta múltiple. En caso de que el informante haya tenido más de un accidente, la respuesta debe reflejar la atención médica recibida por el accidente más grave.

- Ingresó en un hospital________________________☐ 1
- Acudió a un centro de urgencias_______________☐ 2
- Consultó a un médico o enfermera_____________☐ 3
- No hizo ninguna consulta ni intervención________☐ 4
- No sabe____________________________________☐ 8
- No contesta_________________________________☐ 9

## I.- RESTRICCIÓN DE LA ACTIVIDAD (últimas 2 semanas)

**Introducción P.28: Entrevistador/a,** léale al informante: **"A continuación le voy a preguntar sobre restricciones en sus actividades habituales en las 2 últimas semanas, es decir, desde _______ (día que corresponde con dos semanas menos) hasta ayer."**

**28. Durante las últimas 2 semanas, ¿ha tenido que reducir o limitar sus actividades habituales al menos la mitad de un día, por alguno o varios dolores o síntomas?**

- Sí____________ ▢ 1 → **28b- ¿Cuántos días?** └┴┘ NS ▢ 98 NC ▢ 99
- No_____________ ▢ 2

Si P.28 = 1 se activa P.28b

**29. Durante las últimas 2 semanas, ¿se ha visto @obligado/a a quedarse más de la mitad de un día en cama por motivos de salud? (Si ha estado @hospitalizado/a considere también los días pasados en el hospital)**

- Sí___________ ▢ 1 → **29b- ¿Cuántos días?** └┴┘ NS ▢ 98 NC ▢99
- No__________ ▢ 2
- No sabe______ ▢ 8
- No contesta___ ▢ 9

Si P.29 = 1 se activa P.29b

## K.- LIMITACIONES FÍSICAS, SENSORIALES Y COGNITIVAS

**Introducción P.32: Entrevistador/a,** léale al informante: **"A continuación le voy a preguntar sobre situaciones en las que podría encontrarse en su vida diaria. Por favor, no tenga en cuenta los problemas temporales."**

**32. ¿Utiliza gafas o lentillas?**

Nota P.32: **Entrevistador/a,** si el informante es ciego marque la opción "Soy ciego o no puedo ver en absoluto".

- Sí________________________________________ ▢ 1
- No_______________________________________ ▢ 2
- Soy @ciego/a o no puedo ver en absoluto_________ ▢ 3
- No sabe___________________________________ ▢ 8
- No contesta_______________________________ ▢ 9

Si P.32=3,8,9 → Ir a P.34
Si P.32=1 mostrar P33.a
Si P.32=2 mostrar P33.b

**33a. ¿Tiene dificultad para ver utilizando sus gafas o lentillas?**

**33b. ¿Tiene dificultad para ver?**

- No, ninguna dificultad__________________________ ▢ 1
- Sí, alguna dificultad____________________________ ▢ 2
- Sí, mucha dificultad____________________________ ▢ 3
- No puedo ver en absoluto_______________________ ▢ 4
- No sabe_____________________________________ ▢ 8
- No contesta__________________________________ ▢ 9

**34. ¿Utiliza audífono?**

Nota P.34: **Entrevistador/a,** si el informante es sordo marque la opción "Soy sordo profundo".

- Sí____________________________ ▢ 1
- No___________________________ ▢ 2
- Soy @sordo/a @profundo/a________ ▢ 3
- No sabe________________________ ▢ 8
- No contesta_____________________ ▢ 9

Si P.34=3,8,9 → Ir a P.37
Si P.34=1 mostrar P35.a
Si P.34=2 mostrar P35.b

**35a. ¿Tiene dificultad para oír lo que se dice en una conversación con otra persona en un sitio tranquilo utilizando su audífono?**

**35b. ¿Tiene dificultad para oír lo que se dice en una conversación con otra persona en un sitio tranquilo?**

- No, ninguna dificultad________________________ ▢ 1
- Sí, alguna dificultad__________________________ ▢ 2
- Sí, mucha dificultad__________________________ ▢ 3
- No puedo hacerlo en absoluto__________________ ▢ 4
- No sabe__________________________________ ▢ 8
- No contesta_______________________________ ▢ 9

Si P.35 = 4, 8, 9 → Ir a P.37
Si P.34=1 mostrar P36.a
Si P.34=2 mostrar P36.b

**36a. ¿Tiene dificultad para oír lo que se dice en una conversación con otra persona en un sitio más ruidoso aunque utilice su audífono?**

**36b. ¿Tiene dificultad para oír lo que se dice en una conversación con otra persona en un sitio más ruidoso?**

- No, ninguna dificultad________________________ ▢ 1
- Sí, alguna dificultad__________________________ ▢ 2
- Sí, mucha dificultad__________________________ ▢ 3
- No puedo hacerlo en absoluto__________________ ▢ 4
- No sabe__________________________________ ▢ 8
- No contesta_______________________________ ▢ 9

**37. ¿Tiene dificultad para caminar 500 metros sobre un terreno llano sin ningún tipo de ayuda para andar?**

Nota P.37: **Entrevistador/a**, si cree que la pregunta puede ofender al entrevistado dada la situación física evidente (silla de ruedas, amputaciones, etc.) marque la opción "No puedo hacerlo en absoluto".

- No, ninguna dificultad ☐ 1
- Sí, alguna dificultad ☐ 2
- Sí, mucha dificultad ☐ 3
- No puedo hacerlo en absoluto ☐ 4
- No sabe ☐ 8
- No contesta ☐ 9

**38. ¿Tiene dificultad para subir o bajar 12 escalones?**

Nota P.38: **Entrevistador/a**, si cree que la pregunta puede ofender al entrevistado dada la situación física evidente (silla de ruedas, amputaciones, etc.) marque la opción "No puedo hacerlo en absoluto".

- No, ninguna dificultad ☐ 1
- Sí, alguna dificultad ☐ 2
- Sí, mucha dificultad ☐ 3
- No puedo hacerlo en absoluto ☐ 4
- No sabe ☐ 8
- No contesta ☐ 9

Si la persona seleccionada tiene menos de 45 años → Ir a Introducción P.45

**38.a ¿Tiene dificultad para recordar o para concentrarse?**

- No, ninguna dificultad ☐ 1
- Sí, alguna dificultad ☐ 2
- Sí, mucha dificultad ☐ 3
- No puedo hacerlo en absoluto ☐ 4
- No sabe ☐ 8
- No contesta ☐ 9

Si la persona seleccionada tiene menos de 65 años → Ir a Introducción P.45

## L.- LIMITACIONES PARA LA REALIZACIÓN DE LAS ACTIVIDADES DE LA VIDA COTIDIANA

**Introducción P.39: Entrevistador/a,** léale al informante: **"Piense ahora en sus actividades básicas diarias. No tenga en cuenta los problemas temporales."**

**39. De las actividades que le voy a leer, ¿habitualmente tiene dificultad para hacerlas sin ayuda?**

| Actividades | No, ninguna dificultad | Sí, alguna dificultad | Sí, mucha dificultad | No puedo hacerlo por mí @mismo/a | No sabe | No contesta |
|---|---|---|---|---|---|---|
| A. Alimentarse | ▢ (1) | ▢ (2) | ▢ (3) | ▢ (4) | ▢ (8) | ▢ (9) |
| B. Sentarse, levantarse de una silla o de una cama, acostarse | ▢ (1) | ▢ (2) | ▢ (3) | ▢ (4) | ▢ (8) | ▢ (9) |
| C. Vestirse y desvestirse | ▢ (1) | ▢ (2) | ▢ (3) | ▢ (4) | ▢ (8) | ▢ (9) |
| D. Ir al servicio | ▢ (1) | ▢ (2) | ▢ (3) | ▢ (4) | ▢ (8) | ▢ (9) |
| E. Ducharse o bañarse | ▢ (1) | ▢ (2) | ▢ (3) | ▢ (4) | ▢ (8) | ▢ (9) |

Si P39.A=2,3,4 o P39.B=2,3,4 o P39.C=2,3,4 o P39.D=2,3,4 o P39.E=2,3,4 → Ir a Introducción P.40

En otro caso → ir a Introducción P.42

**Introducción P.40: Entrevistador/a,** léale al informante: **"Considerando esta actividad o actividades en las que usted encuentra dificultades para hacerlas".**

**40. ¿Dispone habitualmente de ayuda para realizarlas?**

Sí, al menos para una actividad______ ▢ 1
No______________________________ ▢ 2
No sabe__________________________ ▢ 8
No contesta_______________________ ▢ 9

Si P.40 = 8,9 → Ir a Introducción P.42

Si P.40 =1 mostrar P41.a

Si P.40 =2 mostrar P41.b

**P41a. ¿Necesitaría más ayuda de la que dispone?**
**P41b. ¿Considera que necesita ayuda?**

Sí, al menos para una actividad______ ▢ 1
No____________________________ ▢ 2
No sabe________________________ ▢ 8
No contesta_____________________ ▢ 9

**Introducción P.42: Entrevistador/a,** léale al informante: **"A continuación, le voy a leer una serie de actividades relacionadas con el hogar. De nuevo, no incluya problemas temporales".**

**P42. ¿Habitualmente tiene dificultad para hacer por sí @mismo/a y sin ayuda alguna de estas actividades?**

| Actividades | No, ninguna dificultad | Sí, alguna dificultad | Sí, mucha dificultad | No puedo hacerlo en absoluto | No aplicable (nunca lo ha intentado o necesitado hacerlo) | No Sabe | No Contesta |
|---|---|---|---|---|---|---|---|
| A. Preparar las comidas | ▢ (1) | ▢ (2) | ▢ (3) | ▢ (4) | ▢ (5) | ▢ (8) | ▢ (9) |
| B. Utilizar el teléfono (buscar el número, marcar…) | ▢ (1) | ▢ (2) | ▢ (3) | ▢ (4) | ▢ (5) | ▢ (8) | ▢ (9) |
| C. Realizar compras (comprar la comida, la ropa…) | ▢ (1) | ▢ (2) | ▢ (3) | ▢ (4) | ▢ (5) | ▢ (8) | ▢ (9) |
| D. Tomar sus medicamentos, incluyendo acordarse de la cantidad y el momento en que los debe tomar | ▢ (1) | ▢ (2) | ▢ (3) | ▢ (4) | ▢ (5) | ▢ (8) | ▢ (9) |
| E. Realizar tareas domésticas ligeras como hacer la colada, hacer la cama, limpiar la casa… | ▢ (1) | ▢ (2) | ▢ (3) | ▢ (4) | ▢ (5) | ▢ (8) | ▢ (9) |
| F. Realizar ocasionalmente tareas domésticas que requieren un gran esfuerzo como mover muebles, limpiar las ventanas, transportar la compra… | ▢ (1) | ▢ (2) | ▢ (3) | ▢ (4) | ▢ (5) | ▢ (8) | ▢ (9) |

| | | | | | | | |
|---|---|---|---|---|---|---|---|
| **G. Administrar su propio dinero (pagar recibos, tratar con el banco, firmar cheques…)** | ▢ (1) | ▢ (2) | ▢ (3) | ▢ (4) | ▢ (5) | ▢ (8) | ▢ (9) |

Si P42.A=2,3,4 o P42.B=2,3,4 o P42.C=2,3,4 o P42.D=2,3,4 o P42.E=2,3,4 o P42.F=2,3,4 o P42.G=2,3,4 → Ir a Introducción P.43

En otro caso → Ir a Introducción P.45

**Introducción P.43:** **Entrevistador/a,** léale al informante: **"Considerando esta actividad o actividades en las que usted encuentra dificultades para hacerlas."**

**43. ¿Dispone habitualmente de ayuda para realizarlas?**

Sí, al menos para una actividad______ ▢ 1
No________________________________ ▢ 2
No sabe____________________________ ▢ 8
No contesta_________________________ ▢ 9

Si P.43 = 8,9 → Ir a Introducción P.45

Si P.43 = 1 mostrar P44.a

Si P.43 = 2 mostrar P44.b

**P44a. ¿Necesitaría más ayuda de la que dispone?**

**P44b. ¿Considera que necesita ayuda?**

Sí, al menos para una actividad______ ▢ 1
No________________________________ ▢ 2
No sabe____________________________ ▢ 8
No contesta_________________________ ▢ 9

**Introducción P.45:** **Entrevistador/a,** léale al informante: **"Las siguientes preguntas se refieren al dolor físico que ha podido tener en las últimas 4 semanas".**

**P45. Durante las 4 últimas semanas, ¿qué grado de dolor ha padecido?**

Ninguno__________________ ▢ 1
Muy leve_________________ ▢ 2
Leve______________________ ▢ 3
Moderado_________________ ▢ 4
Severo____________________ ▢ 5
Extremo__________________ ▢ 6
No sabe__________________ ▢ 8
No contesta_______________ ▢ 9

**P46. Durante las 4 últimas semanas, ¿hasta qué punto el dolor afectó a sus actividades cotidianas?**

Nada_____________________ ▢ 1
Un poco__________________ ▢ 2
Moderadamente____________ ▢ 3
Bastante_________________ ▢ 4
Mucho___________________ ▢ 5
No sabe__________________ ▢ 8
No contesta_______________ ▢ 9

## M.- SALUD MENTAL Y ESTRÉS LABORAL

**Introducción P.47:**

**Ahora nos gustaría saber cómo se ha sentido, en general, durante las últimas semanas. Por favor, conteste a todas las preguntas indicando la respuesta que, a su juicio, mejor puede aplicarse a usted. Recuerde que sólo debe responder sobre los problemas recientes y los que tiene ahora, no sobre los que tuvo en el pasado.**

**Es importante que intente contestar todas las preguntas.**

**Entrevistador/a,** léale al informante: **"Voy a entregarle una tarjeta con las preguntas sobre la frecuencia con la que se ha sentido o encontrado como dice cada pregunta. Cuando le pregunte, dígame sólo el número de su respuesta. Tenga en cuenta que las respuestas no son iguales para todas las preguntas".**

Nota P.47: **Entrevistador/a,** el concepto "últimas semanas" lo debe interpretar el informante como desee.

| | 0 | 1 | 2 | 3 | 8 | 9 |
|---|---|---|---|---|---|---|
| 1.- ¿Ha podido concentrarse bien en lo que hacía? | Mejor que lo habitual | Igual que lo habitual | Menos que lo habitual | Mucho menos que lo habitual | No sabe | No contesta |
| 2.- ¿Sus preocupaciones le han hecho perder mucho sueño? | No, en absoluto | No más que lo habitual | Algo más que lo habitual | Mucho más que lo habitual | No sabe | No contesta |
| 3.- ¿Ha sentido que está desempeñando un papel útil en la vida? | Más útil que lo habitual | Igual que lo habitual | Menos útil que lo habitual | Mucho menos útil que lo habitual | No sabe | No contesta |
| 4.- ¿Se ha sentido capaz de tomar decisiones? | Más que lo habitual | Igual que lo habitual | Menos que lo habitual | Mucho menos que lo habitual | No sabe | No contesta |
| 5.- ¿Se ha notado constantemente agobiado y en tensión? | No, en absoluto | No más que lo habitual | Algo más que lo habitual | Mucho más que lo habitual | No sabe | No contesta |
| 6.- ¿Ha tenido la sensación de que no puede superar sus dificultades? | No, en absoluto | No más que lo habitual | Algo más que lo habitual | Mucho más que lo habitual | No sabe | No contesta |
| 7.- ¿Ha sido capaz de disfrutar de sus actividades normales de cada día? | Más que lo habitual | Igual que lo habitual | Menos que lo habitual | Mucho menos que lo habitual | No sabe | No contesta |
| 8 ¿Ha sido capaz de hacer frente adecuadamente a sus problemas? | Más capaz que lo habitual | Igual que lo habitual | Menos capaz que lo habitual | Mucho menos capaz que lo habitual | No sabe | No contesta |
| 9.- ¿Se ha sentido poco feliz o deprimido? | No, en absoluto | No más que lo habitual | Algo más que lo habitual | Mucho más que lo habitual | No sabe | No contesta |
| 10.- ¿Ha perdido confianza en sí mismo? | No, en absoluto | No más que lo habitual | Algo más que lo habitual | Mucho más que lo habitual | No sabe | No contesta |
| 11.- ¿Ha pensado que usted es una persona que no vale para nada? | No, en absoluto | No más que lo habitual | Algo más que lo habitual | Mucho más que lo habitual | No sabe | No contesta |
| 12.- ¿Se siente razonablemente feliz considerando todas las circunstancias? | Más que lo habitual | Igual que lo habitual | Menos que lo habitual | Mucho menos que lo habitual | No sabe | No contesta |

Si P.11 (Cuestionario del Hogar) = 1 para el adulto seleccionado → Ir a P.47.a

Si P.11 (Cuestionario del Hogar) <> 1 para el adulto seleccionado → Ir a Introducción P48

**Introducción P. 47.a: Entrevistador/a**, léale al informante: **"Las siguientes preguntas van dirigidas a conocer cuáles son las condiciones en que realiza el trabajo".**

**47.a- Globalmente y teniendo en cuenta las condiciones en que realiza su trabajo, indique cómo considera Ud. el nivel de estrés de su trabajo según una escala de 1 (nada estresante) a 7 (muy estresante):**

| 1 | 2 | 3 | 4 | 5 | 6 | 7 |
|---|---|---|---|---|---|---|

- No sabe ________________________ ▢ 8
- No contesta ________________________ ▢ 9

**47.b- Teniendo en cuenta las características de su trabajo, indique en qué medida considera su trabajo satisfactorio según una escala de 1 (nada satisfactorio) a 7 (muy satisfactorio):**

| 1 | 2 | 3 | 4 | 5 | 6 | 7 |
|---|---|---|---|---|---|---|

- No sabe ________________________________ ▢ 8
- No contesta ____________________________ ▢ 9

*MÓDULO DE ASISTENCIA SANITARIA*

## N.- CONSULTAS MÉDICAS Y OTROS SERVICIOS AMBULATORIOS

**Introducción P.48: Entrevistador/a**, léale al informante: **"El siguiente grupo de preguntas hace referencia a las consultas con su médico general o médico de familia. Por favor, incluya tanto las visitas a la consulta de su médico, como las visitas domiciliarias y las consultas telefónicas."**

**48. ¿Cuándo fue la última vez que consultó al médico general o médico de familia para usted @mismo/a?**

En las últimas 4 semanas__________________ ▢ 1
Entre 4 semanas y 12 meses_______________ ▢ 2
Hace 12 meses o más_____________________ ▢ 3
Nunca__________________________________ ▢ 4

Si P.48= 2 → Imputar P.49=00 e ir a Introducción P.50
Si P.48= 3,4 → Ir a Introducción P.50

**49. Durante las últimas 4 semanas contando desde ayer, es decir, desde el ________(fecha del día anterior a la entrevista menos 28 días), ¿cuántas veces ha consultado con su médico general o médico de familia para usted @mismo/a?**

|__|__| Nº de veces NS ▢ 98 NC ▢99

**Introducción P.50: Entrevistador/a,** léale al informante: **“Las siguientes preguntas hacen referencia a consultas a especialistas. Se incluyen sólo las visitas a médicos en consultas externas o ambulatorias, pero no las visitas realizadas en el hospital como paciente ingresado o atendido en un hospital de día. No deben incluirse las visitas al dentista, aunque sí al cirujano maxilofacial.”**

**50. ¿Cuándo fue la última vez que consultó a un especialista para usted @mismo/a?**

En las últimas 4 semanas____________________ ▢ 1
Entre 4 semanas y 12 meses________________ ▢ 2
Hace 12 meses o más______________________ ▢ 3
Nunca_____________________________________ ▢ 4
No sabe____________________________________ ▢ 8
No contesta_________________________________ ▢ 9

Si P.50= 2 → Imputar P.51=00 e ir a Filtro P.53

Si P.50= 3, 4, 8, 9 → Ir a Filtro P.53

**51. Durante las últimas 4 semanas contando desde ayer, es decir, desde el ________ (fecha del día anterior a la entrevista menos 28 días), ¿cuántas veces consultó a un especialista para usted @mismo/a?**

|__|__| Nº de veces NS ▢ 98 NC ▢99

Si P.48=1 y P.50=1 → Ir a P.52
En otro caso → Ir a Filtro P.53

**52. El último médico que consultó, ¿era médico de familia/general o era especialista?**

Médico de familia o médico general_______________ ▢ 1
Especialista_____________________________________ ▢ 2
No sabe_________________________________________ ▢ 8
No contesta______________________________________ ▢ 9

Si P.48=1 o P.50=1 → Ir a P.53
En otro caso → Ir a P.58

**53. ¿Dónde tuvo lugar la última consulta realizada durante las últimas 4 semanas?**

Centro de Salud/Consultorio ______ ▢ 01
Ambulatorio/Centro de especialidades ______ ▢ 02
Consulta externa de un hospital ______ ▢ 03
Servicio de Urgencias no hospitalario ______ ▢ 04
Servicio de Urgencias de un hospital ______ ▢ 05
Consulta de médico de una sociedad ______ ▢ 06
Consulta de médico particular ______ ▢ 07
Empresa o lugar de trabajo ______ ▢ 08
Domicilio del entrevistado ______ ▢ 09
Consulta telefónica ______ ▢ 10
Otro lugar ______ ▢ 11
No sabe ______ ▢ 98
No contesta ______ ▢ 99

Si P.53 = 04 ó 05 → Ir a P.57

**54. ¿Cuál fue el motivo principal de esta última consulta?**

Diagnóstico de una enfermedad o problema de salud ______ ▢ 1
Un accidente o agresión ______ ▢ 2
Revisión ______ ▢ 3
Sólo dispensación de recetas ______ ▢ 4
Parte de baja, confirmación o alta ______ ▢ 5
Otros motivos ______ ▢ 6
No sabe ______ ▢ 8
No contesta ______ ▢ 9

Si P.54 <> 1 → Ir a P.56

**55. Y esta última vez, ¿cuánto tiempo pasó desde que empezó a notarse @enfermo/a o sintió que tenía algún problema de salud hasta que pidió cita para consulta?**

Meses ______ └┴┘
Días ______ └┴┘
Horas ______ └┴┘
No sabe ______ ▢ 98
No contesta ______ ▢ 99

Nota P.55: **Entrevistador/a**, puede rellenar las tres casillas (Meses, Días y Horas) o cualquiera de las tres casillas por separado.

**56. Y también esta última vez, ¿cuánto tiempo pasó desde que pidió la cita hasta que le vio el médico?**

Meses ________________________ └┴┘
Días __________________________ └┴┘
Horas _________________________ └┴┘
No sabe ___________________________ ▢ 98
No contesta ________________________ ▢ 99

Nota P.56: **Entrevistador/a**, puede rellenar las tres casillas (Meses, Días y Horas) o cualquiera de las tres casillas por separado.

Si P.53 = 01 ó 02 → Ir a P.57. Disponibles todas las categorías de respuesta.
Si P.53 = 06 ó 07 → Disponibles en P.57 solo respuestas 2, 3, 8 y 9
Si P.53 = 08 → Marcar en P.57 = 4 e Ir a P.58

**57. El médico al que acudió en esta última consulta realizada era de:**

Sanidad Pública (Seguridad Social) ____________________ ▢ 1
Sociedad médica ________________________________ ▢ 2
Consulta privada ________________________________ ▢ 3
Otros (médico de empresa, etc.) ____________________ ▢ 4
No sabe __________________________________ ▢ 8
No contesta _______________________________ ▢ 9

**58. En los últimos 12 meses, es decir, desde _______ (fecha de entrevista menos un año), ¿ha visitado para usted @mismo/a un...?**

| | Sí | No | No sabe | No contesta |
|---|---|---|---|---|
| **Fisioterapeuta** | ▢ (1) | ▢ (2) | ▢ (8) | ▢ (9) |
| **Psicólogo, psicoterapeuta o psiquiatra** | ▢ (1) | ▢ (2) | ▢ (8) | ▢ (9) |
| **Enfermero/a o matrona** | ▢ (1) | ▢ (2) | ▢ (8) | ▢ (9) |

**59. Durante los últimos 12 meses, es decir, desde ______ (fecha de entrevista menos un año), ¿se ha realizado algún análisis?**

Sí__________________ ☐ 1
No __________________ ☐ 2
No sabe______________ ☐ 8
No contesta___________ ☐ 9

Nota P.59: **Entrevistador/a,** se incluye análisis de sangre, orina, heces, etc.

**60. Durante los últimos 12 meses, es decir, desde ______ (fecha de entrevista menos un año), ¿se ha realizado alguna de las siguientes pruebas?**

| | Sí | No | No sabe | No contesta |
|---|---|---|---|---|
| **Radiografía** | ☐ (1) | ☐ (2) | ☐ (8) | ☐ (9) |
| **TAC o escáner** | ☐ (1) | ☐ (2) | ☐ (8) | ☐ (9) |
| **Ecografía** | ☐ (1) | ☐ (2) | ☐ (8) | ☐ (9) |
| **Resonancia magnética** | ☐ (1) | ☐ (2) | ☐ (8) | ☐ (9) |

**60.a- Durante los últimos 12 meses, es decir, desde______ (fecha de hace un año), ¿ha visitado para usted @mismo/a a un…?**

| | Sí | No | No sabe | No contesta |
|---|---|---|---|---|
| Homeópata | ☐ 1 | ☐ 2 | ☐ 8 | ☐ 9 |
| Acupuntor | ☐ 1 | ☐ 2 | ☐ 8 | ☐ 9 |
| Naturista | ☐ 1 | ☐ 2 | ☐ 8 | ☐ 9 |
| Otro profesional de medicina alternativa (osteópata, quiropráctico…) | ☐ 1 | ☐ 2 | ☐ 8 | ☐ 9 |

**61. Durante los últimos 12 meses, es decir, desde ______ (fecha de entrevista menos un año), ¿ha utilizado personalmente alguno de los siguientes servicios asistenciales?**

| | Sí | No | No sabe | No contesta |
|---|---|---|---|---|
| **Atención domiciliaria por una enfermera o matrona** | ☐ (1) | ☐ (2) | ☐ (8) | ☐ (9) |
| **Ayuda domiciliaria para las tareas domésticas o para las personas mayores** | ☐ (1) | ☐ (2) | ☐ (8) | ☐ (9) |
| **Comidas a domicilio para personas mayores** | ☐ (1) | ☐ (2) | ☐ (8) | ☐ (9) |
| **Servicios especiales de transporte a domicilio para acudir a un servicio médico, a un hospital de día, actividades recreativas…** | ☐ (1) | ☐ (2) | ☐ (8) | ☐ (9) |
| **Otros servicios de asistencia domiciliaria** | ☐ (1) | ☐ (2) | ☐ (8) | ☐ (9) |

La tercera opción: *"Comidas a domicilio para personas mayores"* solo aparece si la persona adulta seleccionada es mayor de 65 años.

**Introducción P.62: Entrevistador/a,** léale al informante: **"A continuación voy a hacerle unas preguntas sobre su salud dental."**

**62. ¿Cuándo fue la última vez que visitó al dentista, estomatólogo o higienista dental para usted @mismo/a (es decir, no sólo como acompañante de un niño, de su pareja, etc.)?**

Hace 3 meses o menos____________________ ☐ 1 62.b ¿Cuántas veces en los últimos 3 meses?
└┴┘ NS ☐ 98 NC ☐ 99

Hace más de 3 meses y menos de 6_________ ☐ 2
Hace 6 meses o más pero menos de 12 ______ ☐ 2
Hace 12 meses o más_____________________ ☐ 4
Nunca__________________________________ ☐ 5

Si P.62= 5 → Ir a P.65

**63. La última vez que acudió, ¿cuál o cuáles de los siguientes tipos de asistencia recibió?**

| | Sí | No | No sabe | No contesta |
|---|---|---|---|---|
| **Revisión o chequeo** | ▢ (1) | ▢ (2) | ▢ (8) | ▢ (9) |
| **Limpieza de boca** | ▢ (1) | ▢ (2) | ▢ (8) | ▢ (9) |
| **Empastes (obturaciones), endodoncias** | ▢ (1) | ▢ (2) | ▢ (8) | ▢ (9) |
| **Extracción de algún diente/muela** | ▢ (1) | ▢ (2) | ▢ (8) | ▢ (9) |
| **Fundas, puentes u otro tipo de prótesis** | ▢ (1) | ▢ (2) | ▢ (8) | ▢ (9) |
| **Tratamiento de las enfermedades de las encías** | ▢ (1) | ▢ (2) | ▢ (8) | ▢ (9) |
| **Ortodoncia** | ▢ (1) | ▢ (2) | ▢ (8) | ▢ (9) |
| **Aplicación de flúor** | ▢ (1) | ▢ (2) | ▢ (8) | ▢ (9) |
| **Implantes** | ▢ (1) | ▢ (2) | ▢ (8) | ▢ (9) |
| **Otro tipo de asistencia** | ▢ (1) | ▢ (2) | ▢ (8) | ▢ (9) |

**64. El dentista, estomatólogo o higienista dental al que consultó esta última vez era de:**

- Sanidad Pública (Seguridad Social, ayuntamiento, consulta privada financiada por el gobierno autónomo) ▢ 1
- Sociedad médica ▢ 2
- Consulta privada ▢ 3
- Otros ▢ 4
- No sabe ▢ 8
- No contesta ▢ 9

**65. ¿Cuál es el estado de sus dientes y muelas?**

| | Sí | No | No sabe | No contesta |
|---|---|---|---|---|
| **Tiene caries** | ▢ (1) | ▢ (2) | ▢ (8) | ▢ (9) |
| **Le han extraído dientes/muelas** | ▢ (1) | ▢ (2) | ▢ (8) | ▢ (9) |
| **Tiene dientes/muelas empastados (obturados)** | ▢ (1) | ▢ (2) | ▢ (8) | ▢ (9) |
| **Le sangran las encías al cepillarse o espontáneamente** | ▢ (1) | ▢ (2) | ▢ (8) | ▢ (9) |
| **Se le mueven los dientes/muelas** | ▢ (1) | ▢ (2) | ▢ (8) | ▢ (9) |
| **Lleva fundas (coronas), puentes, otro tipo de prótesis o dentadura postiza** | ▢ (1) | ▢ (2) | ▢ (8) | ▢ (9) |
| **Le faltan dientes/muelas que no han sido sustituidos por prótesis** | ▢ (1) | ▢ (2) | ▢ (8) | ▢ (9) |
| **Tiene o conserva todos sus dientes/muelas naturales** | ▢ (1) | ▢ (2) | ▢ (8) | ▢ (9) |

## O.- HOSPITALIZACIONES, URGENCIAS Y SEGURO SANITARIO

**Introducción P.66: Entrevistador/a,** léale al informante: **“El siguiente grupo de preguntas hace referencia al tiempo pasado en un hospital. Se incluyen todos los tipos de hospital“.**

Si la persona seleccionada es: (hombre) o (mujer de 52 años o más), mostrar P66.a
Si la persona seleccionada es mujer menor de 52 años, mostrar P66.b

**66a. Durante los últimos 12 meses, ¿ha tenido que ingresar como paciente al menos durante una noche?**

**66b. Durante los últimos 12 meses, ¿ha tenido que ingresar como paciente al menos durante una noche excluyendo partos o cesáreas?**

Sí ____________________ ▢ 1
No ____________________ ▢ 2

Nota P.66: **Entrevistador/a**, no deben incluirse las visitas a urgencias ni a hospitales de día. Para mujeres menores de 52 años sí se consideran los ingresos por complicaciones anteriores y posteriores al parto.

Si P.66 = 1 → Ir a P.67

Si P.66 = 2 y es una mujer menor de 52 años → Ir a P.69

Resto de los casos → Ir a P.75

Si la persona seleccionada es: (hombre) o (mujer de 52 años o más), mostrar P67.a
Si la persona seleccionada es mujer menor de 52 años, mostrar P67.b

**67a. ¿Cuántas veces ha estado @hospitalizado/a en estos últimos 12 meses**?

**67b. ¿Cuántas veces ha estado hospitalizada en estos últimos 12 meses**? **Nuevamente, no considere los partos o cesáreas.**

**Nº de veces** |_|_| NS ▢ 98 NC ▢99

*Nota P.67: **Entrevistador/a:** anote 00 si el adulto seleccionado está actualmente hospitalizado en el momento de la entrevista (responde un proxy) y ésta es la única vez que ha estado hospitalizado en los últimos 12 meses.

Si la persona seleccionada es mujer menor de 52 años ---> Ir a P.69
Si la persona seleccionada es (hombre) o (mujer de 52 años o más) --> Ir a FILTRO P.70

**69. Durante los últimos 12 meses, ¿ha tenido que ingresar en un hospital por parto o cesárea?**

Sí ____________________ ▢ 1
No ____________________ ▢ 2
No sabe_______________ ▢ 8
No contesta____________ ▢ 9

Nota P.69: No deben incluirse las visitas a urgencias ni a hospitales de día.

FILTRO P.70
Si P.69= 2, 8, 9 y P.66 = 2 → Ir a P.75
Si P.69= blanco, 2, 8, 9 y P.67 = 1 → Ir a P.70

**70. En relación al último ingreso hospitalario que haya tenido por cualquier motivo, ¿cuántos días estuvo @ingresado/a en el hospital?**

Nº de días |_|_|_| NS ▢ 998 NC ▢ 999

*Nota P.70: **Entrevistador/a:** anote 000 si el adulto seleccionado está actualmente

hospitalizado en el momento de la entrevista (responde un proxy) y ésta es la única vez que ha estado hospitalizado en los últimos 12 meses.

**71. En relación también a este último ingreso hospitalario, ¿cuál fue el motivo de su ingreso en el hospital?**

Intervención quirúrgica ▢ 1
Estudio médico para diagnóstico ▢ 2
Tratamiento médico sin intervención quirúrgica ▢ 3
Parto (incluye cesárea) ▢ 4
Otros motivos ▢ 5
No sabe ▢ 8
No contesta ▢ 9

Si P.71 = 4 → Ir a P.74

**72. ¿Estuvo en lista de espera por este motivo?**

Sí ▢ 1
No ▢ 2
No sabe ▢ 8
No contesta ▢ 9

Si P.72 = 2, 8 ó 9 → Ir a P.74

**73. ¿Cuánto tiempo en meses estuvo en lista de espera desde que le dijeron que tenía que ingresar?**

Nº de meses └┴┘ NS ▢ 98 NC ▢ 99

**74. ¿A cargo de quién corrieron los gastos de su hospitalización?**

Sanidad Pública (Seguridad Social) ▢ 1
Mutualidad obligatoria (MUFACE, ISFAS, etc.) ▢ 2
Sociedad médica privada ▢ 3
A su propio cargo o de su hogar ▢ 4
A cargo de otras personas, organismos o instituciones ▢ 5
No sabe ▢ 8
No contesta ▢ 9

## Hospital de día

**75. Durante los últimos 12 meses, desde el _______ (fecha de entrevista menos un año), ¿ha sido @atendido/a en un Hospital de día para una intervención, tratamiento o hacerse alguna prueba, es decir, permaneciendo durante todo o parte del día pero sin tener que pasar allí la noche?**
**(Incluya ingresos en cama o sillón cama, no incluya estancias en urgencias ni en observación)**

Sí__________________ ☐ 1
No __________________ ☐ 2
No sabe______________ ☐ 8
No contesta___________ ☐ 9

Si P.75 <>1 → Ir a P.78

**76. ¿Cuántos días ha tenido que acudir a un hospital de día, sin tener que pasar la noche?**

|__|__|__| Días NS ☐ 998 NC ☐999

**Nota P.76:** Mínimo un día
Obligatorio: P.76 ≥ 001

**77. ¿Cual fue la razón del último ingreso que haya tenido en el Hospital de día?**

Un tratamiento_______________________________ ☐ 1
Una intervención quirúrgica___________________ ☐ 2
Otros motivos________________________________ ☐ 3
No sabe_____________________________________ ☐ 8
No contesta__________________________________ ☐ 9

## Servicios de urgencia

**Introducción P.78: Entrevistador/a,** léale al informante: "**Para terminar este apartado, voy a preguntarle ahora por su posible utilización de los servicios de urgencias".**

**78. En estos últimos 12 meses, ¿ha tenido que utilizar algún servicio de urgencias por algún problema o enfermedad?**

Sí ________________ ▢ 1
No ________________ ▢ 2

Si P.78= 2 → Ir a P.84

**79. Y en total, ¿cuántas veces tuvo que utilizar un servicio de urgencias en estos 12 meses?**

Nº de veces └┴┴┘ NS ▢ 998 NC ▢ 999

**80. ¿Dónde fue @atendido/a la última vez?**

Nota P.80: se admite respuesta múltiple.

En el lugar donde se encontraba (domicilio, lugar de trabajo, etc.)___▢
En una unidad móvil________________________________▢
En un centro o servicio de urgencias________________________▢
No sabe______________________________________▢
No contesta___________________________________▢

**81. La última vez que utilizó un servicio de urgencias, ¿cuánto tiempo pasó desde que empezó a notarse @enfermo/a o sintió que tenía algún problema de salud hasta que pidió asistencia?**

Nota P.81: **Entrevistador/a**, puede rellenar las tres casillas (Días, Horas y Minutos) o cualquiera de las tres casillas por separado.

Días ____________ └┴┘
Horas ___________ └┴┘
Minutos__________ └┴┘
No sabe ________________ ▢ 98
No contesta _____________ ▢ 99

**82. Y también esta última vez que utilizó un servicio de urgencias, ¿cuánto tiempo pasó desde que pidió asistencia hasta que fue @atendido/a?**

Nota P.82: **Entrevistador/a**, puede rellenar las dos casillas (Minutos y Horas) o cualquiera de las dos casillas por separado.

Horas __________ └┴┘
Minutos_________ └┴┘
No sabe _______________ ▢ 98
No contesta ____________ ▢ 99

**83. Y también esta última vez que utilizó un servicio de urgencias en estos 12 últimos meses, ¿qué tipo de servicio utilizó?**

Hospital de la Sanidad Pública (Seguridad Social) __________________ ▢ 1
Centro o servicio de urgencias no hospitalario de la Sanidad Pública (Seguridad Social). Por ejemplo, centro de salud, ambulatorio etc. ______ ▢ 2
Sanatorio, hospital o clínica privada________________________________ ▢ 3
Servicio privado de urgencias ____________________________________ ▢ 4
Casa de socorro o servicio de urgencias del ayuntamiento _____________ ▢ 5
Otro tipo de servicio ______________________________________________ ▢ 6
No sabe________________________________________________________ ▢ 8
No contesta_____________________________________________________ ▢ 9

**84. ¿Podría decirme ahora, de cuál o cuáles de las siguientes modalidades de Seguro Sanitario (público y/o privado) es titular o @beneficiario/a?**

Sanidad pública (Seguridad Social)_____________________________ ▢
Mutualidades del Estado (MUFACE, ISFAS, MUGEJU) acogidas a la Seguridad Social ____________________________________________ ▢
Mutualidades del Estado (MUFACE, ISFAS, MUGEJU) acogidas a un seguro privado _____________________________________________ ▢
Seguro médico privado, concertado individualmente (sociedades médicas, Colegios Profesionales, etc.) __________________________ ▢
Seguro médico concertado por la empresa _______________________ ▢
No tengo seguro médico ______________________________________ ▢
Otras situaciones ___________________________________________ ▢
No sabe __________________________________________________ ▢
No contesta _______________________________________________ ▢

Nota P.84: **Entrevistador/a**, anote como máximo dos respuestas, las que el informante considere prioritarias.

# P.- CONSUMO DE MEDICAMENTOS

**Introducción P.85: Entrevistador/a,** léale al informante: **"A continuación, le voy a preguntar sobre el consumo de medicamentos en las últimas 2 semanas."**

**85. Durante las 2 últimas semanas, ¿ha consumido algún medicamento que le fuera recetado por un médico?**

Sí ________________ ▢ 1
No ________________ ▢ 2

**86. Durante las últimas 2 semanas, ¿ha consumido algún medicamento, incluyendo medicamentos a base de plantas o vitaminas, que no le fueran recetados por un médico?**

Sí___________________ ▢ 1
No __________________ ▢ 2
No sabe______________ ▢ 8
No contesta___________ ▢ 9

Si P.85=1 o P.86=1 → Ir a P.87
En otro caso → Ir a P.88

**87. A continuación voy a leerle una lista de tipos de medicamentos, por favor dígame ¿cuál o cuáles de ellos ha consumido en las últimas 2 semanas y cuáles le fueron recetados por el médico?**

Nota P.87: **Entrevistador/a,** léale al informante la lista de medicamentos y pregúntele, para cada uno de ellos, si los ha consumido o no en las últimas 2 semanas y, en caso afirmativo, si le fueron o no recetados por algún médico. Para cada medicamento que haya consumido debe cumplimentar la columna **Recetado**.

| | Consumido | | | | Recetado | | | |
|---|---|---|---|---|---|---|---|---|
| | Sí | No | NS | NC | Sí | No | NS | NC |
| **1. Medicinas para el catarro, gripe, garganta, bronquios** | ▢ (1) | ▢ (2) | ▢ (8) | ▢ (9) | ▢ (1) | ▢ (2) | ▢ (8) | ▢ (9) |
| **2. Medicinas para el dolor** | ▢ (1) | ▢ (2) | ▢ (8) | ▢ (9) | ▢ (1) | ▢ (2) | ▢ (8) | ▢ (9) |
| **3. Medicinas para bajar la fiebre** | ▢ (1) | ▢ (2) | ▢ (8) | ▢ (9) | ▢ (1) | ▢ (2) | ▢ (8) | ▢ (9) |
| **4. Reconstituyentes como vitaminas, minerales, tónicos** | ▢ (1) | ▢ (2) | ▢ (8) | ▢ (9) | ▢ (1) | ▢ (2) | ▢ (8) | ▢ (9) |
| **5. Laxantes** | ▢ (1) | ▢ (2) | ▢ (8) | ▢ (9) | ▢ (1) | ▢ (2) | ▢ (8) | ▢ (9) |
| **6. Antibióticos** | ▢ (1) | ▢ (2) | ▢ (8) | ▢ (9) | ▢ (1) | ▢ (2) | ▢ (8) | ▢ (9) |
| **7. Tranquilizantes, relajantes, pastillas para dormir** | ▢ (1) | ▢ (2) | ▢ (8) | ▢ (9) | ▢ (1) | ▢ (2) | ▢ (8) | ▢ (9) |
| **8. Medicamentos para la alergia** | ▢ (1) | ▢ (2) | ▢ (8) | ▢ (9) | ▢ (1) | ▢ (2) | ▢ (8) | ▢ (9) |
| **9. Medicamentos para la diarrea** | ▢ (1) | ▢ (2) | ▢ (8) | ▢ (9) | ▢ (1) | ▢ (2) | ▢ (8) | ▢ (9) |
| **10. Medicinas para el reuma** | ▢ (1) | ▢ (2) | ▢ (8) | ▢ (9) | ▢ (1) | ▢ (2) | ▢ (8) | ▢ (9) |
| **11. Medicinas para el corazón** | ▢ (1) | ▢ (2) | ▢ (8) | ▢ (9) | ▢ (1) | ▢ (2) | ▢ (8) | ▢ (9) |
| **12. Medicinas para la tensión arterial** | ▢ (1) | ▢ (2) | ▢ (8) | ▢ (9) | ▢ (1) | ▢ (2) | ▢ (8) | ▢ (9) |
| **13. Medicinas para el estómago y/o las alteraciones digestivas** | ▢ (1) | ▢ (2) | ▢ (8) | ▢ (9) | ▢ (1) | ▢ (2) | ▢ (8) | ▢ (9) |
| **14. Antidepresivos, estimulantes** | ▢ (1) | ▢ (2) | ▢ (8) | ▢ (9) | ▢ (1) | ▢ (2) | ▢ (8) | ▢ (9) |
| **15. Píldoras para no quedar embarazada (solo para mujeres)** | ▢ (1) | ▢ (2) | ▢ (8) | ▢ (9) | ▢ (1) | ▢ (2) | ▢ (8) | ▢ (9) |
| **16. Hormonas para la menopausia (solo para mujeres)** | ▢ (1) | ▢ (2) | ▢ (8) | ▢ (9) | ▢ (1) | ▢ (2) | ▢ (8) | ▢ (9) |
| **17. Medicamentos para adelgazar** | ▢ (1) | ▢ (2) | ▢ (8) | ▢ (9) | ▢ (1) | ▢ (2) | ▢ (8) | ▢ (9) |
| **18. Medicamentos para bajar el colesterol** | ▢ (1) | ▢ (2) | ▢ (8) | ▢ (9) | ▢ (1) | ▢ (2) | ▢ (8) | ▢ (9) |
| **19. Medicamentos para la diabetes** | ▢ (1) | ▢ (2) | ▢ (8) | ▢ (9) | ▢ (1) | ▢ (2) | ▢ (8) | ▢ (9) |
| **20. Medicamentos para el tiroides** | ▢ (1) | ▢ (2) | ▢ (8) | ▢ (9) | ▢ (1) | ▢ (2) | ▢ (8) | ▢ (9) |
| **21. Productos homeopáticos** | ▢ (1) | ▢ (2) | ▢ (8) | ▢ (9) | ▢ (1) | ▢ (2) | ▢ (8) | ▢ (9) |
| **22. Productos naturistas** | ▢ (1) | ▢ (2) | ▢ (8) | ▢ (9) | ▢ (1) | ▢ (2) | ▢ (8) | ▢ (9) |
| **23. Otros medicamentos** | ▢ (1) | ▢ (2) | ▢ (8) | ▢ (9) | ▢ (1) | ▢ (2) | ▢ (8) | ▢ (9) |

Sólo se activará la correspondiente P.87_Recetado para las opciones en las que P.87_Consumido = 1
Si el adulto seleccionado es hombre, NO aparecerán las opciones 15 y 16.

Si el adulto seleccionado es mujer de **65** años o más, NO aparecerá la opción 15.

## Q.- PRÁCTICAS PREVENTIVAS

**Introducción P.88: Entrevistador/a,** léale al informante: **"A continuación, le voy a preguntar sobre la vacunación de la gripe."**

**88. ¿Se ha vacunado de la gripe en la última campaña?**

Sí__________________ ▢ 1
No __________________ ▢ 2

Si P.88 = 2 → Ir a Introducción P.89

**88b. ¿Cuándo se vacunó?**

Año ________________________ └┴┴┴┘
Mes ________________________ └┴┘

**Introducción P.89:** **Entrevistador/a,** léale al informante: **"Ahora le voy a preguntar sobre su tensión arterial, su nivel de colesterol y su nivel de azúcar".**

**89. ¿Le ha tomado la tensión alguna vez un profesional sanitario?**

Sí__________________ ☐ 1
No_________________ ☐ 2
No sabe____________ ☐ 8
No contesta_________ ☐ 9

Nota P.89: **Entrevistador/a,** se incluye la toma de tensión en farmacias.

Si P.89= 2, 8, 9 → Ir a P.91

**90. La última vez que le tomaron la tensión fue:**

En los últimos 12 meses________________________ ☐ 1
Hace 1 año o más pero menos de 2 años________ ☐ 2
Hace 2 años o más pero menos de 3 años_______ ☐ 3
Hace 3 años o más pero menos de 5 años_______ ☐ 4
Hace 5 años o más___________________________ ☐ 5
No sabe_____________________________________ ☐ 8
No contesta_________________________________ ☐ 9

Nota P.90: **Entrevistador/a,** se incluye la toma de tensión en farmacias.

**91. ¿Le han medido alguna vez su nivel de colesterol en sangre?**

Sí__________________ ☐ 1
No_________________ ☐ 2
No sabe____________ ☐ 8
No contesta_________ ☐ 9

Si P.91 = 2, 8, 9 → Ir a P.93

**92. ¿Cuándo fue la última vez que le midieron su nivel de colesterol en sangre?**

En los últimos 12 meses____________________ ▢ 1
Hace 1 año o más pero menos de 3 años______ ▢ 2
Hace 3 años o más pero menos de 5 años_____ ▢ 3
Hace 5 años o más________________________ ▢ 4
No sabe_________________________________ ▢ 8
No contesta______________________________ ▢ 9

**93. ¿Le ha medido alguna vez su nivel de azúcar en sangre un profesional sanitario?**

Sí________________ ▢ 1
No_______________ ▢ 2
No sabe__________ ▢ 8
No contesta________ ▢ 9

Si P.93 = 2, 8, 9 → Ir a Introducción P.95

**94. ¿Cuándo fue la última vez que un profesional sanitario le midió su nivel de azúcar en sangre?**

En los últimos 12 meses____________________ ▢ 1
Hace 1 año o más pero menos de 3 años______ ▢ 2
Hace 3 años o más pero menos de 5 años_____ ▢ 3
Hace 5 años o más________________________ ▢ 4
No sabe_________________________________ ▢ 8
No contesta______________________________ ▢ 9

**Introducción P.95:** **Entrevistador/a**, léale al informante: **"Las siguientes preguntas hacen referencia a la prueba de sangre oculta en heces y a la colonoscopia."**

**95. ¿Alguna vez le han hecho una prueba de sangre oculta en heces?**

Sí________________ ▢ 1
No_______________ ▢ 2
No sabe__________ ▢ 8
No contesta________ ▢ 9

Si P.95 = 2, 8, 9 → Ir a P.98

**96. ¿Cuándo fue la última vez que le hicieron una prueba de sangre oculta en heces?**

En los últimos 12 meses ☐ 1
Hace 1 año o más pero menos de 2 años ☐ 2
Hace 2 años o más pero menos de 3 años ☐ 3
Hace 3 años o más pero menos de 5 años ☐ 4
Hace 5 años o más ☐ 5
No sabe ☐ 8
No contesta ☐ 9

**97. ¿Cuál de las siguientes fue la principal razón por la que se hizo esta última prueba de sangre oculta en heces?**

Por algún problema, síntoma o enfermedad ☐ 1
Por consejo de su médico de atención primaria o especialista, aunque no tenía ningún problema ☐ 2
Porque recibió una carta, le llamaron por teléfono o le dijeron en su centro de salud que si se quería hacer esta prueba ☐ 3
Otras razones ☐ 4
No sabe ☐ 8
No contesta ☐ 9

**98. ¿Le han realizado alguna vez una colonoscopia?**

Sí ☐ 1
No ☐ 2
No sabe ☐ 8
No contesta ☐ 9

Nota P.98: **Entrevistador/a,** si el informante tiene dudas sobre qué es una colonoscopia, puede comentarle que se trata de una: "Exploración del interior del colon a través de un aparato óptico".

Si P.98 = 2, 8, 9 y el adulto seleccionado es mujer → Ir a Introducción P.100
Si P.98 = 2, 8, 9 y el adulto seleccionado es hombre → Ir a Introducción P.106

**99. ¿Cuándo fue la última vez que le hicieron una colonoscopia?**

En los últimos 12 meses________________________1
Hace 1 año o más pero menos de 5 años_________2
Hace 5 años o más pero menos de 10 años_______3
Hace 10 años o más___________________________4
No sabe____________________________________8
No contesta_________________________________9

Nota P.99: **Entrevistador/a,** si el informante tiene dudas sobre qué es una colonoscopia, puede comentarle que se trata de una: "Exploración del interior del colon a través de un aparato óptico".

Si el adulto seleccionado es mujer → Ir a Introducción P.100
SI el adulto seleccionado es hombre → Ir a Introducción P.106

**Introducción P.100:** **Entrevistador/a**, léale al informante: **"Las siguientes preguntas hacen referencia a las pruebas de mamografía y de citología vaginal".**

**100. ¿Le han hecho alguna vez una mamografía?**

Sí ______________________ ☐ 1
No _____________________ ☐ 2
No sabe _________________ ☐ 8
No contesta ______________ ☐ 9

Nota P.100: **Entrevistador/a,** si la informante tiene dudas sobre la mamografía puede comentarle que se trata de una radiografía de mama.

Si P.100 = 2, 8, 9 → Ir a P.103

**101. ¿Cuándo fue la última vez que le hicieron una mamografía?**

En los últimos 12 meses________________________ ▢ 1
Hace 1 año o más pero menos de 2 años_________ ▢ 2
Hace 2 años o más pero menos de 3 años________ ▢ 3
Hace 3 años o más____________________________ ▢ 4
No sabe______________________________________ ▢ 8
No contesta___________________________________ ▢ 9

Nota P.101: **Entrevistador/a,** si la informante tiene dudas sobre la mamografía puede comentarle que se trata de una radiografía de mama.

**102. ¿Cuál de las siguientes fue la principal razón por la que se hizo esta última mamografía?**

Por algún problema, síntoma o enfermedad _________________________ ▢ 1
Por consejo de su médico de atención primaria o especialista, aunque no tenía ningún problema____________________________________ ▢ 2
Porque recibió una carta, le llamaron por teléfono o le dijeron en su centro de salud que si se quería hacer esta prueba_______________ ▢ 3
Otras razones__________________________________________________ ▢ 4
No sabe _______________________________________________________ ▢ 8
No contesta ____________________________________________________ ▢ 9

**103. ¿Le han hecho alguna vez una citología vaginal?**

Sí ______________________________ ▢ 1
No _____________________________ ▢ 2
No sabe _________________________ ▢ 8
No contesta ______________________ ▢ 9

Nota P.103: **Entrevistador/a,** si la informante tiene dudas sobre la citología vaginal, puede comentarle que se trata de una muestra de células.

Si P.103 = 2, 8, 9 → Ir a Introducción P.106

**104. ¿Cuándo fue la última vez que le hicieron una citología vaginal?**

En los últimos 12 meses__________________________1
Hace 1 año o más pero menos de 2 años____________2
Hace 2 años o más pero menos de 3 años___________3
Hace 3 años o más pero menos de 5 años___________4
Hace 5 años o más_______________________________5
No sabe_____________________________________8
No contesta__________________________________9

Nota P.104: **Entrevistador/a,** si la informante tiene dudas sobre la citología vaginal, puede comentarle que se trata de una muestra de células.

**105. ¿Cuál de las siguientes fue la principal razón por la que se hizo esta última citología?**

Por algún problema, síntoma o enfermedad ________________________ ▢ 1
Por consejo de su médico de atención primaria o especialista, aunque no tenía ningún problema________________________________________ ▢ 2
Porque recibió una carta, le llamaron por teléfono o le dijeron en su centro de salud que si se quería hacer esta prueba___________________ ▢ 3
Otras razones______________________________________________ ▢ 4
No sabe __________________________________________________ ▢ 8
No contesta ______________________________________________ ▢ 9

## R.- NECESIDADES DE ATENCIÓN MÉDICA NO CUBIERTAS

**Introducción P.106:** **Entrevistador/a,** léale al informante: **"Por diferentes motivos puede tener que esperar para recibir atención médica e incluso puede no llegar a recibirla."**

**106. En los últimos 12 meses, ¿alguna vez ha tardado demasiado en recibir asistencia médica por una lista de espera demasiado larga?**

Sí_______________________________________ ▢ 1
No______________________________________ ▢ 2
No he necesitado asistencia médica____________ ▢ 3
No sabe__________________________________ ▢ 8
No contesta_______________________________ ▢ 9

Nota P.106: **Entrevistador/a,** si la respuesta es NO, se debe indagar si se refiere a la categoría 2 (Necesité atención médica y la recibi o tardé en recibirla pero no por ese motivo) o a la categoría 3 (No necesité atención médica).

**107. En los últimos 12 meses, ¿alguna vez ha tardado demasiado en recibir o ha carecido de asistencia médica por dificultades relacionadas con el transporte o la distancia?**

Sí ☐ 1
No ☐ 2
No he necesitado asistencia médica ☐ 3
No sabe ☐ 8
No contesta ☐ 9

Nota P.107: **Entrevistador/a,** si la respuesta es NO, se debe indagar si se refiere a la categoría 2 (Necesité atención médica y tardé en recibirla pero no por ese motivo o necesité atención sanitaria y la he recibido) o a la categoría 3 (No necesité atención médica).

**108. En los últimos 12 meses, ¿ha necesitado alguno de los siguientes tipos de atención sanitaria y no se lo pudo permitir por motivos económicos?**

| | Sí | No | No lo he necesitado | No sabe | No contesta |
|---|---|---|---|---|---|
| **A. Atención médica** | ☐ (1) | ☐ (2) | ☐ (3) | ☐ (8) | ☐ (9) |
| **B. Atención dental** | ☐ (1) | ☐ (2) | ☐ (3) | ☐ (8) | ☐ (9) |
| **C. Algún medicamento que le habían recetado** | ☐ (1) | ☐ (2) | ☐ (3) | ☐ (8) | ☐ (9) |
| **D. Atención de salud mental (Consulta al psicólogo o psiquiatra, por ejemplo)** | ☐ (1) | ☐ (2) | ☐ (3) | ☐ (8) | ☐ (9) |

Nota P.108: **Entrevistador/a,** si la respuesta es NO, se debe indagar si se refiere a la categoría 2 (Necesité atención sanitaria pero no pude permitírmela por motivos económicos o necesité la atención sanitaria y la he recibido) o a la categoría 3 (No necesité atención sanitaria).

# *MÓDULO DE DETERMINANTES DE LA SALUD*

## S.- CARACTERÍSTICAS FÍSICAS

**Introducción P.109: Entrevistador/a,** léale al informante: **"Ahora le voy a hacer unas preguntas sobre su talla y su peso."**

**109. ¿Podría decirme cuánto mide, aproximadamente, sin zapatos?**

|__|__|__| cm   NS ▢ 998   NC ▢ 999

**110. ¿Y cuánto pesa, aproximadamente, sin zapatos ni ropa?**

|__|__|__| kg   NS ▢ 998   NC ▢ 999

## T.- ACTIVIDAD FÍSICA

**Introducción P.111: Entrevistador/a,** léale al informante: **"Ahora voy a hacerle unas preguntas sobre la actividad física que realiza."**

**111. ¿Cuál de estas posibilidades describe mejor su actividad principal en el centro de trabajo, centro de enseñanza, hogar (labores domésticas)…?**

@Sentado/a la mayor parte de la jornada ________ ▢ 1
De pie la mayor parte de la jornada sin efectuar grandes desplazamientos o esfuerzos ________ ▢ 2
Caminando, llevando algún peso, efectuando desplazamientos frecuentes ________ ▢ 3
Realizando tareas que requieren gran esfuerzo físico ________ ▢ 4
No aplicable ________ ▢ 5
No sabe________ ▢ 8
No contesta________ ▢ 9

**112. ¿Cuál de estas posibilidades describe mejor la frecuencia con la que realiza alguna actividad física en su tiempo libre?**

No hago ejercicio. El tiempo libre lo ocupo de forma casi completamente sedentaria (leer, ver la televisión, ir al cine, etc.) ______ ☐ 1
Hago alguna actividad física o deportiva ocasional (caminar o pasear en bicicleta, jardinería, gimnasia suave, actividades recreativas que requieren un ligero esfuerzo, etc.) ______ ☐ 2
Hago actividad física varias veces al mes (deportes, gimnasia, correr, natación, ciclismo, juegos de equipo, etc.) ______ ☐ 3
Hago entrenamiento deportivo o físico varias veces a la semana ______ ☐ 4
No sabe ______ ☐ 8
No contesta ______ ☐ 9

Si el adulto seleccionado tiene 70 o más años → Ir a P.120.

**Introducción P.113:** Entrevistador/a, léale al informante: **"Para concluir este apartado sobre el ejercicio físico y por su importancia para la salud, le pedimos que conteste también las siguientes preguntas acerca del tiempo que dedicó a actividades físicas en los <u>últimos 7 días</u>. Por favor, responda cada una de las preguntas aunque no se considere una persona activa. Piense en las actividades que hace como parte del trabajo, en la casa, para ir de un sitio a otro y también las que hace en su tiempo libre de descanso, ejercicio o deporte".**

**113- En primer lugar piense en las actividades <u>intensas</u> que realizó en los <u>últimos 7 días</u>. Actividades intensas son las que requieren gran esfuerzo físico y le hacen respirar mucho más fuerte de lo normal, como levantar objetos pesados, cavar, ejercicios aeróbicos o pedalear rápido en bicicleta. Piense solamente en aquellas que realizó por lo menos durante 10 minutos seguidos.**

**Durante los últimos 7 días, ¿cuántos días realizó actividades físicas intensas?**

- Días por semana ______ |_| → desplegable: 1, 2, ..., 7
- Ningún día con actividad física intensa ______ ☐ 0 → Ir a P.115
- No sabe ______ ☐ 8 → Ir a P.115
- No contesta ______ ☐ 9 → Ir a P.115

**114- En uno de esos días, ¿cuánto tiempo en total le dedicó a actividades físicas intensas?**

- Horas por día ________________ |_ _|
- Minutos por día________________ |_ _|
- No sabe ____________________ ▢ 98
- No contesta ________________ ▢ 99

**115- Ahora piense por favor en todas aquellas actividades moderadas que realizó en los últimos 7 días. Actividades moderadas son aquellas que requieren un esfuerzo físico moderado que le hace respirar algo más fuerte de lo normal, como llevar pesos ligeros, andar en bicicleta a velocidad regular o jugar dobles de tenis. Piense solamente en aquellas que realizó por lo menos durante 10 minutos seguidos.**

**Durante los últimos 7 días, ¿cuántos días realizó actividades físicas moderadas? Por favor, no incluya caminar.**

- Días por semana ______________________ |_| → desplegable: 1, 2, ..., 7
- Ningún día con actividad física moderada _ ▢ 0 → Ir a P.117
- No sabe______________________________ ▢ 8 → Ir a P.117
- No contesta___________________________ ▢ 9 → Ir a P.117

**116- En uno de esos días, ¿cuánto tiempo en total le dedicó a actividades físicas moderadas?**

- Horas por día ____________________ |_ _|
- Minutos por día____________________ |_ _|
- No sabe ________________________ ▢ 98
- No contesta ____________________ ▢ 99

Se admite respuesta múltiple, pueden ir cumplimentadas la primera y la segunda. Las opciones NS/NC son incompatibles con las demás.

**117- Ahora piense acerca del tiempo que dedicó a caminar en los últimos 7 días. Esto incluye caminar en el trabajo, en la casa, para ir de un sitio a otro, o lo que paseó por deporte, ejercicio o placer. Piense solamente en aquellas ocasiones en que caminó por lo menos durante 10 minutos seguidos.**

- Días por semana ____________________ | _ | → desplegable: 1, 2, ..., 7
- Ningún día más de 10 minutos seguidos __ ☐ 0 → Ir a P.119
- No sabe ____________________ ☐ 8 → Ir a P.119
- No contesta ____________________ ☐ 9 → Ir a P.119

**118- En los últimos 7 días, ¿cuánto tiempo en total dedicó a caminar en un día normal de la semana?**

- Horas por día ____________________ | _ _ |
- Minutos por día____________________ | _ _ |
- No sabe ____________________ ☐ 98
- No contesta ____________________ ☐ 99

**119- La última pregunta se refiere al tiempo que permaneció @sentado/a en los últimos 7 días. Incluya el tiempo @sentado/a en el trabajo, la casa, en clase, estudiando, leyendo y en el transporte, tiempo libre o viendo la televisión.**

**En los últimos 7 días, ¿cuánto tiempo permaneció @sentado/a en un día normal de la semana?**

- Horas por día ____________________ | _ _ |
- Minutos por día____________________ | _ _ |
- No sabe ____________________ ☐ 98
- No contesta ____________________ ☐ 99

# U.- ALIMENTACIÓN

**Introducción P.120:** Entrevistador/a, léale al informante: **“Le voy a hacer unas preguntas sobre alimentación.”**

**120. ¿Con qué frecuencia consume los siguientes alimentos?**

| Alimentos | Frecuencias de consumo | | | | | | | |
|---|---|---|---|---|---|---|---|---|
| | Una o más veces al día | De 4 a 6 veces a la semana | Tres veces a la semana | Una o dos veces a la semana | Menos de 1 vez a la semana | Nunca | No sabe | No contesta |
| Fruta fresca (excluyendo zumos) | ☐ 1 Nº ☐ | ☐ 2 | ☐ 3 | ☐ 4 | ☐ 5 | ☐ 6 | ☐ 8 | ☐ 9 |
| Carne (pollo, ternera, cerdo, cordero...) | ☐ 1 | ☐ 2 | ☐ 3 | ☐ 4 | ☐ 5 | ☐ 6 | ☐ 8 | ☐ 9 |
| Huevos | ☐ 1 | ☐ 2 | ☐ 3 | ☐ 4 | ☐ 5 | ☐ 6 | ☐ 8 | ☐ 9 |
| Pescado | ☐ 1 | ☐ 2 | ☐ 3 | ☐ 4 | ☐ 5 | ☐ 6 | ☐ 8 | ☐ 9 |
| Pasta, arroz, patatas | ☐ 1 | ☐ 2 | ☐ 3 | ☐ 4 | ☐ 5 | ☐ 6 | ☐ 8 | ☐ 9 |
| Pan, cereales | ☐ 1 | ☐ 2 | ☐ 3 | ☐ 4 | ☐ 5 | ☐ 6 | ☐ 8 | ☐ 9 |
| Verduras, ensaladas y hortalizas | ☐ 1 Nº ☐ | ☐ 2 | ☐ 3 | ☐ 4 | ☐ 5 | ☐ 6 | ☐ 8 | ☐ 9 |
| Legumbres | ☐ 1 | ☐ 2 | ☐ 3 | ☐ 4 | ☐ 5 | ☐ 6 | ☐ 8 | ☐ 9 |
| Embutidos y fiambres | ☐ 1 | ☐ 2 | ☐ 3 | ☐ 4 | ☐ 5 | ☐ 6 | ☐ 8 | ☐ 9 |
| Productos lácteos (leche, queso, yogur) | ☐ 1 | ☐ 2 | ☐ 3 | ☐ 4 | ☐ 5 | ☐ 6 | ☐ 8 | ☐ 9 |
| Dulces (galletas, bollería, mermeladas, cereales con azúcar, caramelos) | ☐ 1 | ☐ 2 | ☐ 3 | ☐ 4 | ☐ 5 | ☐ 6 | ☐ 8 | ☐ 9 |
| Refrescos con azúcar | ☐ 1 | ☐ 2 | ☐ 3 | ☐ 4 | ☐ 5 | ☐ 6 | ☐ 8 | ☐ 9 |
| Comida rápida (pollo frito, bocadillos, pizzas, hamburguesas) | ☐ 1 | ☐ 2 | ☐ 3 | ☐ 4 | ☐ 5 | ☐ 6 | ☐ 8 | ☐ 9 |
| Aperitivos o comidas saladas de picar (patatas fritas, ganchitos, galletitas saladas) | ☐ 1 | ☐ 2 | ☐ 3 | ☐ 4 | ☐ 5 | ☐ 6 | ☐ 8 | ☐ 9 |
| Zumo natural de frutas o verduras | ☐ 1 Nº ☐ | ☐ 2 | ☐ 3 | ☐ 4 | ☐ 5 | ☐ 6 | ☐ 8 | ☐ 9 |

Al marcar “Una o más veces al día” en la Opción 1 (Fruta fresca excluyendo zumos) debe aparecer la pregunta emergente **120.A ‘¿Cuántas piezas de fruta consume a diario?**’ con un desplegable para seleccionar valores desde 01 hasta 10, NS y NC.

Al marcar “Una o más veces al día” en la Opción 7 (Verduras, ensaladas y hortalizas) debe aparecer la pregunta emergente **120.B ‘¿Cuántas veces al día?**’ con un desplegable para seleccionar valores desde 01 hasta 10, NS y NC.

Al marcar “Una o más veces al día” en la Opción 15 (Zumo natural de frutas o verduras) debe aparecer la pregunta emergente **120.C ‘¿Cuántas veces al día?**’ con un desplegable para seleccionar valores desde 01 hasta 10, NS y NC.

*Solo si P120.1 (Fruta fresca) = 2 y P120.15 (Zumo natural) = 2 → se pasa a la pregunta 120.D
*En otro caso → Ir a P120F

**120.D Considerando ahora de manera conjunta su consumo de fruta fresca y zumos naturales a la semana, ¿toma a diario al menos uno de ellos?**

Sí ______________________________ ☐ 1
No ______________________________ ☐ 2
No sabe __________________________ ☐ 8
No contesta _______________________ ☐ 9

Si P120.D = 2, 8, 9 → Ir a P. 120F

**120.E ¿Cuántas piezas de fruta o vasos de zumo natural consume a diario?**

Se inserta un desplegable para seleccionar valores desde 01 hasta 10, NS y NC.

NS ☐ 98    NC ☐ 99

## U2. HIGIENE DENTAL

**120F.- Ahora le voy a preguntar sobre su higiene dental, ¿cuántas veces al día se cepilla los dientes habitualmente?**

- Nunca ______________________________ ☐ 1
- Ocasionalmente, no todos los días ________ ☐ 2
- Una vez al día _______________________ ☐ 3
- Dos veces al día ______________________ ☐ 4
- Tres o más veces al día ________________ ☐ 5
- No sabe ____________________________ ☐ 8
- No contesta __________________________ ☐ 9

## V.- CONSUMO DE TABACO Y EXPOSICIÓN AL HUMO DE TABACO

**Introducción P.121: Entrevistador/a,** léale al informante: **"Las siguientes preguntas se refieren al consumo de tabaco y a la exposición al humo de tabaco."**

**121. ¿Podría decirme si fuma?**

Sí, fumo a diario ........................................................ ▢ 1
Sí fumo, pero no a diario ........................................... ▢ 2
No fumo actualmente pero he fumado antes .................. ▢ 3
No fumo ni he fumado nunca de manera habitual ............ ▢ 4
No sabe ....................................................................... ▢ 8
No contesta .................................................................. ▢ 9

Si P.121= 3, 4, 8, 9 → Ir a P.126

**122. ¿Qué tipo de tabaco fuma con mayor frecuencia?**

Cigarrillos (incluido el tabaco de liar) ............................ ▢ 1
Puros ........................................................................... ▢ 2
Tabaco de pipa ............................................................. ▢ 3
Otros ............................................................................ ▢ 4
No sabe ........................................................................ ▢ 8
No contesta ................................................................... ▢ 9

Nota P.122: **Entrevistador/a,** solamente es posible una respuesta.

Si P.121=1 y P.122=1 → Ir a P.123
Si P.121=1 y P.122<>1 → Ir a P.124
En otro caso → Ir a P.126

**123. Por término medio, ¿cuántos cigarrillos fuma usted al día?**

Número de cigarrillos: |__|__| NS ▢ 98 NC ▢ 99

**124. ¿A qué edad empezó a fumar?**

Edad en años: |_ _| NS ▢ 98 NC ▢ 99

**125. Durante los últimos 12 meses, ¿cuántos intentos serios de dejar de fumar ha realizado en los que consiguiera estar al menos 24 horas sin fumar?**

NS ▢ 8 NC ▢ 9

Aparecerá un desplegable con los valores “0”, “1”, “2”, “3”, “4”, “5 ó más”.

**126. ¿Con qué frecuencia está @expuesto/a al humo del tabaco en lugares cerrados? Considere solo aquellas situaciones en las que son otras personas las que están fumando.**

Nunca o casi nunca........................................ ▢ 1
Menos de una hora al día................................ ▢ 2
Entre 1 y 5 horas al día .................................. ▢ 3
Más de 5 horas al día..................................... ▢ 4
No sabe........................................................... ▢ 8
No contesta ..................................................... ▢ 9

Nota P.126: **Entrevistador/a,** se refiere a la exposición al humo del tabaco en casa, en el trabajo, lugares públicos, restaurantes...

# W.- CONSUMO DE ALCOHOL

**Introducción P.127: Entrevistador/a,** léale al informante: **“Las siguientes preguntas se refieren al consumo de bebidas alcohólicas durante los últimos 12 meses.”**

**127. Durante los últimos 12 meses, ¿con qué frecuencia ha tomado bebidas alcohólicas de cualquier tipo (es decir, cerveza, vino, licores, bebidas destiladas y combinados u otras bebidas alcohólicas)?**

- A diario o casi a diario ☐ 01
- 5-6 días por semana ☐ 02
- 3-4 días por semana ☐ 03
- 1-2 días por semana ☐ 04
- 2-3 días en un mes ☐ 05
- Una vez al mes ☐ 06
- Menos de una vez al mes ☐ 07
- No en los últimos 12 meses, he dejado de tomar alcohol ☐ 08
- Nunca o solamente unos sorbos para probarlo a lo largo de toda la vida ☐ 09
- No sabe ☐ 98
- No contesta ☐ 99

Si P.127 = 05, 06, 07 → Ir a Introducción P.129

Si P.127 = 08, 09, 98, 99 → Ir a Introducción P.130

**128. Durante los últimos 12 meses, en una semana en que desarrolló su actividad habitual, ¿cuántas bebidas que contengan alcohol consumió a lo largo de la semana?**

| | Cervezas con alcohol Sí ☑ No ☐ NS ☐ NC ☐ | Vinos, Cava Sí ☐ No ☑ NS ☐ NC ☐ | Vermuts, fino, jerez Sí ☐ No ☑ NS ☐ NC ☐ | Licores, anís, pacharán Sí ☑ No ☐ NS ☐ NC ☐ | Whisky, coñac, combinados ... Sí ☐ No ☑ NS ☐ NC ☐ | Bebidas 'locales' sidra, carajillo Sí ☐ No ☐ NS ☐ NC ☑ |
|---|---|---|---|---|---|---|
| Lunes | 0 Cervezas | Vasos,copas | Copas | 0 Copas | Copas | Vasos, copas |
| Martes | 0 Cervezas | Vasos,copas | Copas | 0 Copas | Copas | Vasos, copas |
| Miércoles | 0 Cervezas | Vasos,copas | Copas | 0 Copas | Copas | Vasos, copas |
| Jueves | 0 Cervezas | Vasos,copas | Copas | 0 Copas | Copas | Vasos, copas |
| Viernes | 0 Cervezas | Vasos,copas | Copas | 0 Copas | Copas | Vasos, copas |
| Sábado | 0 Cervezas | Vasos,copas | Copas | 0 Copas | Copas | Vasos, copas |
| Domingo | 0 Cervezas | Vasos,copas | Copas | 0 Copas | Copas | Vasos, copas |

**Introducción P.129: Entrevistador/a,** entregue al informante la tarjeta Ad-2 y léale: **"En esta tarjeta puede observar la equivalencia de las bebidas alcohólicas más comunes con la llamada bebida estándar. De acuerdo a estas equivalencias, podría contestarme..."**

Si el adulto seleccionado es hombre, presentar enunciado P129.A
Si el adulto seleccionado es mujer, presentar enunciado P129.B

**129.A Durante los últimos 12 meses, ¿con qué frecuencia ha tomado 6 o más bebidas estándar en una misma ocasión? (Por "ocasión" entendemos tomar las bebidas en un intervalo aproximado de 4-6 horas).**

**129.B Durante los últimos 12 meses, ¿con qué frecuencia ha tomado 5 o más bebidas estándar en una misma ocasión? (Por "ocasión" entendemos tomar las bebidas en un intervalo aproximado de 4-6 horas).**

A diario o casi a diario.............................. ▢ 01
De 5 a 6 días por semana........................ ▢ 02
De 3 a 4 días por semana........................ ▢ 03
De 1 a 2 días por semana........................ ▢ 04
De 2 a 3 días en un mes.......................... ▢ 05
Una vez al mes......................................... ▢ 06
Menos de una vez al mes......................... ▢ 07
No en los últimos 12 meses..................... ▢ 08
Nunca en toda mi vida.............................. ▢ 09
No sabe................................................... ▢ 98
No contesta.............................................. ▢ 99

## X.- APOYO AFECTIVO Y PERSONAL

**Introducción P.130: Entrevistador/a**, léale al informante: **"Ahora le voy a hacer unas preguntas sobre el apoyo afectivo que recibe".**

**130.- Entrevistador/a,** léale al informante: **"Voy a entregarle una tarjeta con diferentes situaciones de apoyo afectivo y personal que suelen ocurrir en la vida cotidiana. Al lado de cada situación hay un conjunto de respuestas. Por favor, lea cada situación y dígame una por una el número de la respuesta que mejor refleje su situación".**

| | Mucho menos de lo que deseo | Menos de lo que deseo | Ni mucho ni poco | Casi como deseo | Tanto como deseo | No sabe | No contesta |
|---|---|---|---|---|---|---|---|
| 1.- Recibo visitas de mis amigos y familiares | ☐ 1 | ☐ 2 | ☐ 3 | ☐ 4 | ☐ 5 | ☐ 8 | ☐ 9 |
| 2.- Recibo ayuda en asuntos relacionados con mi casa | ☐ 1 | ☐ 2 | ☐ 3 | ☐ 4 | ☐ 5 | ☐ 8 | ☐ 9 |
| 3.- Recibo elogios y reconocimientos cuando hago bien mi trabajo | ☐ 1 | ☐ 2 | ☐ 3 | ☐ 4 | ☐ 5 | ☐ 8 | ☐ 9 |
| 4.- Cuento con personas que se preocupan de lo que me sucede | ☐ 1 | ☐ 2 | ☐ 3 | ☐ 4 | ☐ 5 | ☐ 8 | ☐ 9 |
| 5.- Recibo amor y afecto | ☐ 1 | ☐ 2 | ☐ 3 | ☐ 4 | ☐ 5 | ☐ 8 | ☐ 9 |
| 6.- Tengo la posibilidad de hablar con alguien de mis problemas en el trabajo o en la casa | ☐ 1 | ☐ 2 | ☐ 3 | ☐ 4 | ☐ 5 | ☐ 8 | ☐ 9 |
| 7.- Tengo la posibilidad de hablar con alguien de mis problemas personales y familiares | ☐ 1 | ☐ 2 | ☐ 3 | ☐ 4 | ☐ 5 | ☐ 8 | ☐ 9 |
| 8.- Tengo la posibilidad de hablar con alguien de mis problemas económicos | ☐ 1 | ☐ 2 | ☐ 3 | ☐ 4 | ☐ 5 | ☐ 8 | ☐ 9 |
| 9.- Recibo invitaciones para distraerme y salir con otras personas | ☐ 1 | ☐ 2 | ☐ 3 | ☐ 4 | ☐ 5 | ☐ 8 | ☐ 9 |
| 10.- Recibo consejos útiles cuando me ocurre algún acontecimiento importante en mi vida | ☐ 1 | ☐ 2 | ☐ 3 | ☐ 4 | ☐ 5 | ☐ 8 | ☐ 9 |
| 11.- Recibo ayuda cuando estoy enfermo en la cama | ☐ 1 | ☐ 2 | ☐ 3 | ☐ 4 | ☐ 5 | ☐ 8 | ☐ 9 |

## Y.- CUIDADO A OTRAS PERSONAS CON PROBLEMAS DE SALUD

**Introducción P.133: Entrevistador/a**, léale al informante: **“Las siguientes preguntas tratan de conocer si dedica tiempo al cuidado de alguna persona con problemas de salud.”**

**133. ¿Cuida, al menos una vez a la semana, de alguna persona mayor o de alguien que tenga una dolencia crónica? No lo considere si forma parte de su trabajo.**

Sí__________________________ ☐ 1
No_________________________ ☐ 2
No sabe____________________ ☐ 8
No contesta________________ ☐ 9

Si P.133 <> 1 → Ir a FILTRO INGRESOS

**134. La persona o personas a las que cuida son:**

Familiares________________________ ☐ 1
Otras personas____________________ ☐ 2
No sabe__________________________ ☐ 8
No contesta_______________________ ☐ 9

Nota P.134: **Entrevistador/a**, en caso de que las dos respuestas sean posibles, el informante debe contestar pensando en la persona a la que más tiempo dedica.

**135. En total, ¿cuántas horas a la semana dedica al cuidado de esta/s persona/s?**

Menos de 10 horas a la semana______________________ ☐ 1
10 horas o más a la semana pero menos de 20__________ ☐ 2
20 horas a la semana o más_________________________ ☐ 3
No sabe__________________________________________ ☐ 8
No contesta_______________________________________ ☐ 9

## Z.- INGRESOS

### FILTRO INGRESOS

**1-** Si **Informante C.Hogar = P. Seleccionada** en C.Adultos (Nº Orden INF = Nº Orden adulto seleccionado) entonces → **Ir a Introducción P.136 (Preguntas de ingresos).**

**2-** Si **Informante C.Hogar <> P. Seleccionada** en C.Adultos (Nº Orden INF <> Nº Orden adulto seleccionado) y:

- **a. P.25 (C.Hogar) = 1 (Sí) → Ir a Introducción P.136 (Preguntas de ingresos)**
- **b. P.25 (C.Hogar) = 2 (No)**, entonces:
  - i. Si P.26 (C.Hogar) <> 98 (“No sabe”) y P.28 (C.Hogar) <> 999998 y P.29 <> 98 → Ir a **Filtro P.140 (DNI)**
  - ii. Si P.26 (C.Hogar) = {01,…,08} y P.28 (C.Hogar) = 999998 y P.29 (C.Hogar) = 98 → Leer Introducción P.136 e **Ir a P138.D**
  - iii. Si P.26 (C.Hogar) = {01,…,08} y P.28 (C.Hogar) = 999998 y P.29 (C.Hogar) <> 98 → Ir a **Filtro P.140 (DNI)**
  - iv. Si P.26 (C.Hogar) = 98 (“No sabe’’) y P.28 (C. Hogar) = 999998 y P.29 (C. Hogar) = 98 → **Ir a Introducción P.136**

v. Si P.26 (C.Hogar) = 98 (“No sabe’’) y P.28 (C. Hogar) = 999998 y P.29 (C. Hogar) <> 98 → **Ir a Filtro P.140 (DNI)**

vi. Si P.26 (C.Hogar) = 98 (“No sabe’’) y P.28 (C. Hogar) <> 999998 → Ir a **Filtro P.140 (DNI)**

En el caso 2b.ii y 2b.iv, si además el Informante C.Hogar (P.9) es ‘Otra persona’ (P.9 de C.Hogar = ‘OTRA PERSONA’) y Proxy_2 = 2 (‘NO’) ➔ Debe aparecer la nota:

**Entrevistador/a,** si el informante es el mismo que respondió a las preguntas de ingresos en el cuestionario de hogar, responda “No contesta” y vaya a la siguiente pregunta.

**Introducción P.136:** **Entrevistador/a,** léale al informante: **“Para poder hacer comparaciones entre los distintos tipos de problemas de salud de las familias, es necesario conocer unos datos muy básicos sobre el nivel de ingresos de la familia.**

**“A continuación le voy a preguntar por los ingresos regulares del hogar. No incluya los ingresos de las personas residentes empleadas en el hogar ni de los huéspedes fijos.”**

**136. De los siguientes tipos de ingresos que le voy a leer, ¿podría decirme cuáles de ellos reciben usted y los demás miembros de su hogar?**
**Debe considerar las fuentes de cada miembro del hogar y las conjuntas.**

Nota P.136: Se admite respuesta múltiple.

- Ingresos del trabajo (por cuenta propia o ajena) ☐
- Prestación y subsidios por desempleo ☐
- Pensión por jubilación o viudedad ☐
- Pensión por invalidez o incapacidad ☐
- Prestaciones económicas por hijo a cargo u otras prestaciones económicas como ayudas a la familia... ☐
- Prestaciones o subvenciones relacionadas con la vivienda ☐
- Prestaciones o subvenciones relacionadas con la educación ☐
- Otros ingresos regulares / Otro subsidio o prestación social regular ☐
- Ninguna fuente de ingresos ☐
- No sabe ☐
- No contesta ☐

Las respuestas “Ninguna fuente de ingresos”, “No sabe” y “No contesta” son excluyentes, no permiten otra respuesta marcada.

Si entre las respuestas 01 y 08 hay una única respuesta marcada → Ir a **P.138B**
Si entre las respuestas 01 y 08 hay más de una respuesta marcada→ Ir a **P.137**

Si P.136 = 98 (“No sabe”) → Ir a **P138.A**
Si P.136 = 09, 99 (“Ninguna fuente de ingresos” o “No contesta”) → Ir a **Filtro P.140**

**137. De estas fuentes de ingresos monetarios del hogar citadas, ¿cuál diría que es la principal?**

Ir a **P.138C**

**138A. Aunque no pueda en este momento especificar las fuentes de ingresos, ¿podría decir cuál es aproximadamente el ingreso mensual neto de todo el hogar (es decir, sumando todas las fuentes y descontando las retenciones por impuestos, Seguridad Social, etc.)?**

**138B. Pensando en la fuente de ingresos que ha mencionado, ¿podría decir cuál es aproximadamente el ingreso mensual neto de todo el hogar (es decir, sumando todas las fuentes y descontando las retenciones por impuestos, Seguridad Social, etc.)?**

**138C. Pensando en las fuentes de ingresos que ha mencionado, ¿podría decir cuál es aproximadamente el ingreso mensual neto de todo el hogar (es decir, sumando todas las fuentes y descontando las retenciones por impuestos, Seguridad Social, etc.)?**

**138D. ¿Podría decir cuál es el importe mensual aproximado de los ingresos del hogar, (es decir, sumando todas las fuentes y descontando las retenciones por impuestos, Seguridad Social, etc.)?**

Cantidad |___|___|___|___|___|___| euros → Ir a **Filtro P.140**

No sabe__________________________ ☐ 999998

No contesta_______________________ ☐ 999999 → Ir a **Filtro P.140**

**139. Si desconoce el valor exacto de los ingresos del hogar, ¿podría decirme cuál de los intervalos siguientes representa mejor el ingreso mensual neto de todo su hogar, tras las deducciones por los impuestos, Seguridad Social, etc.?**

Menos de 570 euros ☐ 01
De 570 a menos de 800 euros ☐ 02
De 800 a menos de 1050 euros ☐ 03
De 1050 a menos de 1.300 euros ☐ 04
De 1.300 a menos de 1.550 euros ☐ 05
De 1.550 a menos de 1.800 euros ☐ 06
De 1.800 a menos de 2.200 euros ☐ 07
De 2.200 a menos de 2.700 euros ☐ 08
De 2.700 a menos de 3.600 euros ☐ 09
De 3.600 a menos de 4.500 euros ☐ 10
De 4.500 a menos de 6.000 euros ☐ 11
De 6.000 euros en adelante ☐ 12
No sabe ☐ 98
No contesta ☐ 99

**Filtro P.140:** Si la persona seleccionada tiene relleno el campo NIF por estar en la lista inicial de personas precargadas de Padrón → Ir a P.141.

**140. Para finalizar, ¿le importaría facilitarme su DNI, número de la tarjeta de residencia o pasaporte?**

**140.1-Tipo de Documento 140.2-Nº de Documento 140.3-Letra final del NIF o NIE**

NIF ☐ 1
Pasaporte ☐ 2
NIE (tarjeta de residente) ☐ 3
No sabe ☐ 8
No contesta ☐ 9

|_|_|_|_|_|_|_|_|_| - |_|

Si P140.1 = 1 ó 3 → activar P140.2 y P140.3
Si P140.1 = 2 → activar P140.2
Si P140.1 = 8 ó 9, entonces imputar P140.2=blanco, P140.3=blanco y saltar a P.141

# INFORMACIÓN SOBRE LA ENTREVISTA

**141. Idioma**

**Entrevistador/a,** anote el idioma empleado principalmente por el informante para responder al Cuestionario de Adulto:

- Castellano ________________ ▢ 1
- Catalán ________________ ▢ 2
- Valenciano ________________ ▢ 3
- Euskera ________________ ▢ 4
- Gallego________________ ▢ 5
- Inglés ________________ ▢ 6
- Otro ________________ ▢ 7

**142. Nota final**

**Entrevistador/a:** Anote las observaciones que considere oportunas al final de este cuestionario.

| **Observaciones** |
| --- |
| ........................................................................................................................................ |
| ........................................................................................................................................ |
| ........................................................................................................................................ |
| ........................................................................................................................................ |

**FIN DEL CUESTIONARIO DE ADULTOS PASAR AL PARTE DE TRABAJO**
